# Reactivity and Stability of (Hetero)Benzylic Alkenes via the Wittig Olefination Reaction

**DOI:** 10.3390/molecules29020501

**Published:** 2024-01-19

**Authors:** Ajmir Khan, Mohammed G. Sarwar, Sher Ali

**Affiliations:** 1 School of Packaging, Michigan State University, 448 Wilson Road, East Lansing, MI 48824, USA; 2 Scops Coating Technologies, 4942 Dawn Ave, East Lansing, MI 48823, USA; 3 Department of Food Engineering, Faculty of Animal Science and Food Engineering (FZEA), University of São Paulo (USP), Pirassununga 13635-900, SP, Brazil; alisher@usp.br

**Keywords:** Wittig olefination, electron-withdrawing groups, electron-donating groups, heterocycles, benzylic position

## Abstract

Wittig olefination at hetero-benzylic positions for electron-deficient and electron-rich heterocycles has been studied. The electronic effects of some commonly used protective groups associated with the *N*-heterocycles were also investigated for alkenes obtained in the context of the widely employed Wittig olefination reaction. It was observed that hetero-benzylic positions of the pyridine, thiophene and furan derivatives were stable after Wittig olefination. Similarly, electron-withdrawing groups (EWGs) attached to *N*-heterocycles (indole and pyrrole derivatives) directly enhanced the stability of the benzylic position during and after Wittig olefination, resulting in the formation of stable alkenes. Conversely, electron-donating group (EDG)-associated *N*-heterocycles boosted the reactivity of benzylic alkene, leading to lower yields or decomposition of the olefination products.

## 1. Introduction

Heterocyclic compounds represent the most diverse family of organic compounds [1]. The importance of heterocyclic compounds can be highlighted by their widespread presence and impact across various scientific fields [2,3]. They play vital roles in disciplines such as biochemistry, medicinal chemistry, natural products and other scientific domains [4]. This family has grown extensively, with an increasing number of heterocyclic compounds being identified or synthesized regularly. This expansion of heterocycles can be attributed to considerable synthetic research efforts along with their significant synthetic applications [5]. An endeavor aimed at expanding the versatility of these heterocycles or their derivatives involves the introduction of double bonds via Wittig olefination. The Wittig reaction was initially explored mainly for its effectiveness in the preparation of olefins. However, recent years have witnessed its multi-faceted applications in the synthesis of heterocycles or their derivatives [6,7,8]. 

Georg Wittig started exploring the Wittig reaction in 1954 [9]. The Wittig reaction stands as one of the most indispensable and essential named reactions in the realm of organic chemistry [10]. Wittig olefination represents a chemical process in which an aldehyde or ketone reacts with a triphenyl phosphonium ylide, commonly referred to as a Wittig reagent, resulting in the formation of an alkene and triphenylphosphine oxide [11,12]. Thus, Wittig olefination ranks as the most commonly employed technique for generating alkenes through synthesis [13]. Despite the existence of alternative methods such as Peterson olefination and Tebbe methylenation, the Wittig reaction continues to retain its status as the foremost and most widely utilized technique for generating carbon–carbon double bonds from carbonyl compounds [14,15]. Thus, the Wittig reaction holds a prominent role in the total synthesis of natural products as well as in the synthesis of pharmaceutical compounds [9,16].

Wittig reagents generally exhibit a high tolerance for carbonyl substrates containing various functional groups, such as epoxide, –OR, –OH, aromatic nitro and ester groups [12,17]. They can even accommodate nitrile groups and C=O if conjugated with the ylide, as in the case of stabilized ylides [11,18]. However, there can be challenges with sterically hindered ketones, leading to slow reactions and low yields, especially with stabilized ylides [19]. On such occasions, chemists often prefer the Horner–Wadsworth–Emmons (HWE) reaction, which utilizes phosphonate esters [20]. Another limitation worth noting is the inherent instability of aldehydes, which can undergo polymerization, oxidation or decomposition. In a process known as the tandem oxidation-Wittig reaction, aldehydes are formed in situ through the oxidation of the corresponding alcohols [21,22].

In this work, our investigation focused on the stability of (hetero)benzylic alkenes that come into being via a widely employed Wittig olefination reaction. Our findings demonstrated that heterocycles, such as thiophene, furan and pyridine derivatives, and electron-withdrawing groups (EWGs), such as tosyl, mesyl, boc and acyl attached to (hetero)aryls, significantly enhance the stability of the benzylic position during and/or after Wittig olefination, resulting in the formation of stable olefinic products. Conversely, electron-rich heterocycles or electron-donating groups (EDGs), such as alkyl, Silyl and benzyl associated with (hetero)aryls, amplify the reactivity at (hetero)benzylic positions, leading to reduced yields or the decomposition of olefination products during the isolation and work-up process. This study will give very thoughtful insights into the construction of heterocyclic derivatives, particularly where simple olefination needs a Wittig reaction.

## 2. Results and Discussion

**Figure 1:**
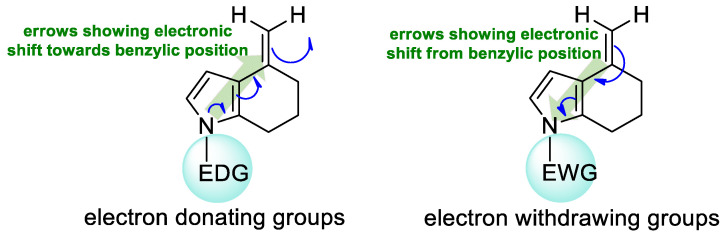
EWGs and EDGs decrease or enhance the reactivity of the corresponding alkene.

During our study of heterocyclic ring expansion reactions using hypervalent iodine, we observed that some of the substrates prepared via Wittig olefination were easily isolated. However, some ketones were decomposed during the work-up process or obtained in very low yields upon exposure to the Wittig reaction [23,24]. To investigate the reason, we decided to observe the decomposition behavior of the expected alkenes via a series of heterocyclic substrates. Several heterocyclic substrates containing N, O and S atoms in their cyclic structure were prepared. Some of the heterocyclic ketones (such as pyrroles) were prepared by introducing electron-withdrawing protective groups (EWGs) or electron-donating protective groups (EDGs). The aim of these EWGs and EDGs was to decrease or enhance the reactivity at the benzylic position for the expected alkenes after Wittig olefination of the heterocycles (Figure 1). EDGs associated with hetero(aryl) derivatives destabilizes the double bond of the alkene that forms during the reaction, making it more nucleophilic. On the other hand, EWGs attached to hetero(aryl) derivatives stabilizes the alkene during the reaction, making it less nucleophilic; thus, it can be easily isolated.

To investigate the electronic effects of different heterocycles in Wittig olefination, we prepared different substrates, specifically (hetero)benzylic ketones, describing them in the following text.

### 2.1. Synthesis of Furans

**Scheme 1:**
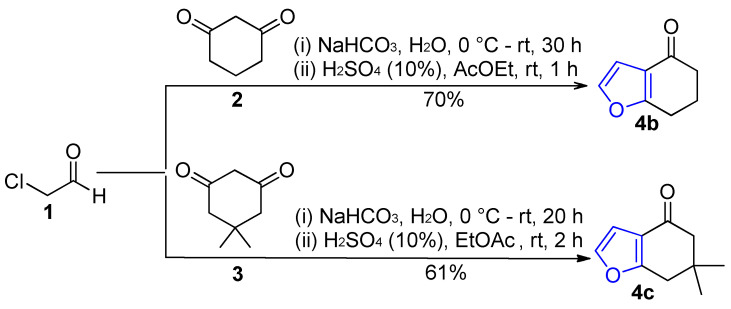
Synthesis of fused furans **4b** and **4c**.

Different heterocyclic substrates, including furan, indole and pyrrole derivatives, were prepared in order to study the electronic effect on Wittig olefination. Furan derivative **4b** was synthesized using commercially available chloroacetaldehyde (**1**) and 1,3-cyclohexanedione (**2**), in the presence of NaHCO_3_ as a base, with a 70% yield (Scheme 1) [25]. Similarly, the reaction of **1** with dimedone (**3**) in the presence of NaHCO_3_, provided the corresponding furan derivative **4c** with a 61% yield [26]. 

**Scheme 2:**
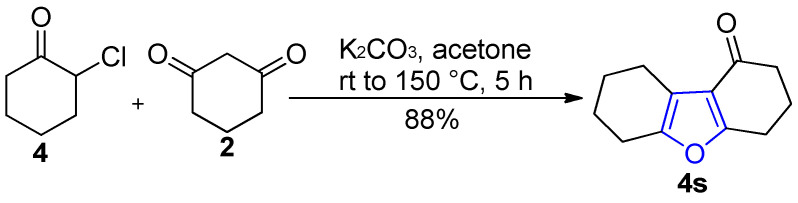
Feist–Benary furan synthesis of **4s**.

Similarly, furan **4s** was prepared from the treatment of **2** and *α*-halogenated carbonyl compound **4** using the same protocol as applied in the literature (Scheme 2) [27]. The spectral data of the compound was matched with previously reported literature data [28]. 

### 2.2. Synthesis of Indoles

**Scheme 3:**
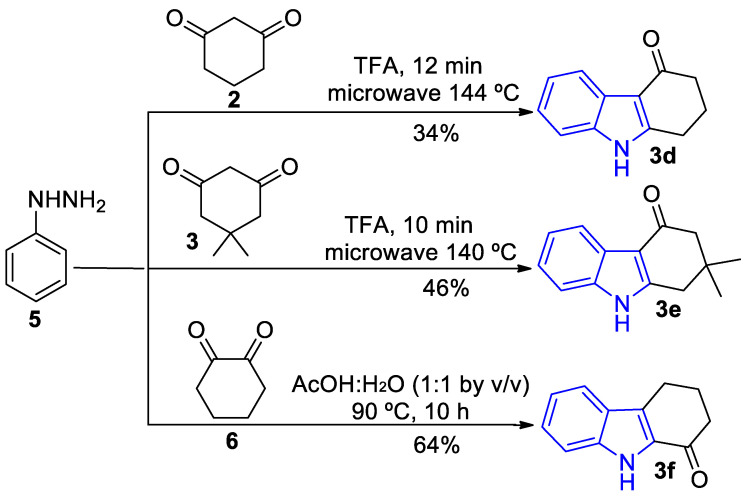
Synthesis of fused indoles **3d**–**3f**.

Indoles **3d** and **3e** were prepared by means of the Fischer reaction from commercially available phenylhydrazine (**5**) treatment with **2** and dimedone (**3**) in acidic conditions [29]. Similarly, indole **3f** was prepared from commercially available 1,2-cyclohexanedione (**6**) and **5**, using the same protocol as in the literature (Scheme 3) [30].

### 2.3. Synthesis of Fused Pyrroles

Some of the *N*-substituted pyrroles were prepared directly from the corresponding fused furan or pyrrole. For example, the Paal–Knorr pyrrole condensation of furan **4s** and **4b** with CH_3_NH_2_ using a catalytic amount of PTSA allowed the synthesis of *N*-substituted pyrroles **4t** and **4u** with 25% and 98% yields, respectively (entries 1 and 2, Table 1) [31]. Heating a mixture of furan **4b** and *m*-chloroaniline (1:5 by mole) in a sealed tube together in the presence of *p*-TsOH (few crystals), using xylene at 160 °C for 24 h, gave **4v** with a 73% yield (entry 3). Conversion of commercially available pyrrole **3w** to *N*-substituted pyrrole **4w** was typically carried out in THF with 1.0 equiv of *n*-BuLi and MeI at 0 °C to room temperature (entry 4) [32]. 

### 2.4. Synthesis of Tetrahydroisoquinoline

**Scheme 4:**
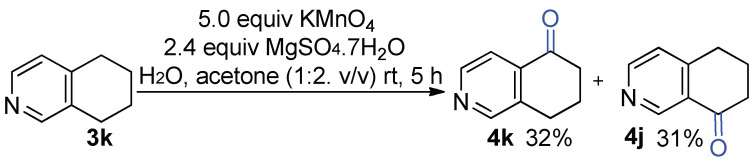
Synthesis of tetrahydroisoqunoline derivatives **4k** and **4j**.

Two tetrahydroisoqunoline derivatives, **4k** and **4j**, were isolated with 32% and 31% yields when tetrahydroisoqunoline **3k** was subjected to KMNO_4_ and MgSO_4_·7H_2_O in acetone:H_2_O (1:2 *v*/*v*) (Scheme 4). Benzylic oxidation took place at position five for **4k** and at position eight for **4j**. The obtained compounds were characterized via NMR and compared with previously reported spectral data [33]. 

### 2.5. Protection of Indole and Pyrrole Derivatives

The acidic protons of indoles and pyrroles were protected with different protecting groups (electron-withdrawing or electron-donating groups) aiming to determine the electronic effects on Witting olefination. Moreover, this acidic hydrogen can also interfere with the Wittig olefination reaction. Indole **3d** was treated with electrophiles (AcCl and TsCl) using 1.5 equiv of NaH (60% dispersion in mineral oil) in THF [34]. The resulting acylated and tosylated indoles, **4d** and **4e**, were isolated with 92% and 62% yields, respectively (entries 1 and 2, Table 2). Similarly, indole **3e** was also protected with acetyl and tosyl chloride, and **4f** and **4g** were isolated with 84% and 72% yields, respectively (entries 3 and 4).

In the subsequent protection reaction, indole **3f** was treated with Boc_2_O and the corresponding protected indole **4h** was isolated with a 67% yield (entry 5). Indole **3f** and pyrrole **3w** were reacted with 3.0 equiv of NaOH as a base in dichloroethane (DCE), and the resulting mesylate-indole **4i** and mesylate-pyrrole **4o** were isolated with 64% and 81% yields, respectively (entries 6 and 7). Using 1.5 equiv of NaH (60% dispersion in mineral oil) in THF with **3w**, the tosyl and Boc-protected pyrroles (**4p** and **4q**) were isolated with 98% and 75% yields, respectively (entries 8 and 9). Furthermore, **3w** was protected with benzyl chloride using KOH in DMSO, which afforded **4r** with 90% yield (entry 10). 

**Figure 2:**
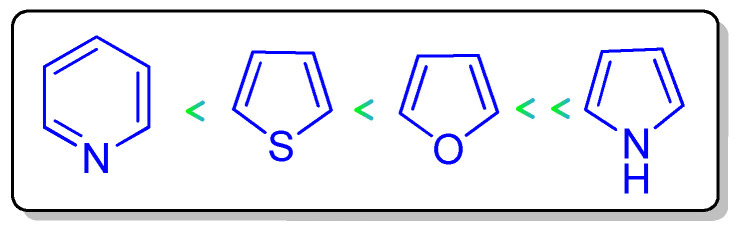
The reactivity trend of pyrrole, furan, thiophene and pyridine.

In the reactivity trend of some common heterocycles, where pyrrole is more reactive than furan, furan is more reactive than thiophene, and thiophene is more reactive than pyridine (Figure 2). This trend was also verified when (hetero)benzylic alkenes (where the heterocycles were pyrrole, furan, thiophene and pyridine) were isolated with different yields. The more reactive the heterocycles, the more they will push the electron density towards the corresponding *exo*-methylene double bond of the alkene products and thus will be less stable. Due to the higher reactivity of pyrrole or indole (Figure 2), their respective benzylic positions can be expected to be more reactive compared to furan, thiophene or pyridine. To modulate the reactivity, we incorporated electron-withdrawing protecting groups (such as acetyl-, tosyl-, mesyl- and boc-) to the prepared or commercially available pyrrole and indole derivatives. Conversely, electron-donating protecting groups (such as benzyl-, silyl-, methyl-) were also incorporated into the pyrrole or indole derivatives with the aim of evaluating the reactivity of (hetero)benzylic alkenes.

### 2.6. Heterocyclic Wittig Olefination

In our preliminary study, we aimed to evaluate the electronic effect of aromaticity on the benzylic position. To achieve this, we conducted two experiments under similar conditions, focusing on the olefination at the benzylic and non-benzylic positions of the substrates. Thus, thiophene derivatives that have benzylic ketone (**4a**) and non-benzylic ketone (**4aa**) were subjected to the Wittig olefination reaction followed by their transformation into the corresponding exocyclic olefinic products, **5a** and **5aa**, with 97% and 69% yields, respectively (entries 1 and 2, Table 3). The relatively lower yield (69%) observed for **5aa** can be attributed to the non-involvement of the carbonyl with an aromatic ring in **4aa**. However, the carbonyl associated with hetero(benzyl) in **4a** underwent in almost full conversion to the respective alkene. In the follow-up study, furan derivatives **4b** and **4c** underwent successful conversion into their respective exocyclic olefinic products **5b** and **5c**, with yields of 86% and 48%, respectively. The lower yield obtained for **5c** was attributed to the steric hindrance imposed by the two methyl groups present on substrate **4c** (entry 4).

Next, acetyl-protected indoles **4d** and **4e** were treated with a Wittig reagent. However, no olefinic products were isolated for either ketone; instead, the protection group (acetyl) was removed (entries 5 and 6). On the other hand, tosyl-protected indoles **4f** and **4g** were successfully converted to their corresponding olefinic products in 82% and 58% yields, respectively (entries 7 and 8). Again, the relatively lower yield (58%) observed for **4g** could be attributed to the steric hindrance, forced by the two methyl groups present on the substrate **4g**. In the subsequent experiment, boc-protected indole **4h** was exposed to Wittig reaction conditions, aiming to observe their influence on the resulting methylene derivative **5h**, which was obtained in good yield (91%) (entry 9). However, for the mesyl-protected indole **4i,** the isolated olefinic product **5i** was also deprotected with the Wittig reagent (entry 10). DCM was used as a co-solvent because of the partial solubility of indole **4i** in Et_2_O.

After the study of protected (EWG) indole derivatives, we extended our investigation to pyridine derivatives (such as isoquinolinones) to determine the stability of the resulting methylene products. Using Wittig conditions, both **4j** and **4k** were effectively transformed into the corresponding exocyclic methylene derivatives **5j** and **5k**, yielding 88% and 62%, respectively (entries 11 and 12). In the subsequent experiment, thiochroman-4-one (**4l**) was also subjected to the evaluation of the stability of the olefinic product. The resultant methylenethiochroman **5l** was isolated with a 72% yield (entry 13). 

To determine the resulting alkene stability, chromane substrates were selected, featuring one with an electron-withdrawing substituent (such as fluorinated-chormane) and another with an electron-donating substituent (such as methoxy-chormane). A nearly identical Wittig olefination protocol was employed for both **4m** and **4n** (entries 14 and 15). After 5 h, the progress of the reactions was determined via TLC analysis, indicating the absence of the reactants (**4m** and **4n**). After completion of the reaction, fluorinated-chromane **5m** was successfully isolated with a 90% yield. However, the anticipated product for the methoxy-substituted chromane (**5n**) was not isolated. The TLC analysis after the work-up process revealed multiple spots, and these spots increased further when the crude mixture was subjected to silica gel chromatography. There was a clear indication that the electron-withdrawing effect of fluorine reduced the reactivity of methylene in **5m** relative to **5n** where the methoxy effect was prominent, and we were unable to keep the compound in pure form for further characterization. 

**Scheme 5:**
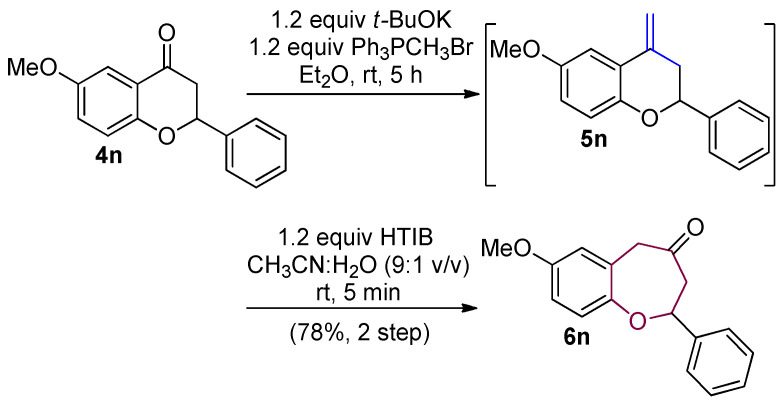
Wittig olefination of **4n**, followed by an HTIB-mediated ring expansion reaction.

After the unsuccessful isolation of the expected olefinic product for **4n**, our subsequent goal was to capture the intermediate (olefinic product) before decomposition in the work-up process. Our strategy was to generate the resulting methylene product but using a minimal amount of Wittig reagent (1.2 equiv) with the aim of minimizing any complexation in the subsequent step, where the reaction mixture was directly exposed (without work-up and isolation process) to an iodine(III) reagent (Hydroxy(tosyloxy)iodobenzene (HTIB) dissolved in CH_3_CN:H_2_O (9:1 *v*/*v*)), with the potential to induce rearrangement and incorporate the *exo*-methylenic carbon into the ring (Scheme 5) [23]. 

**Scheme 6:**
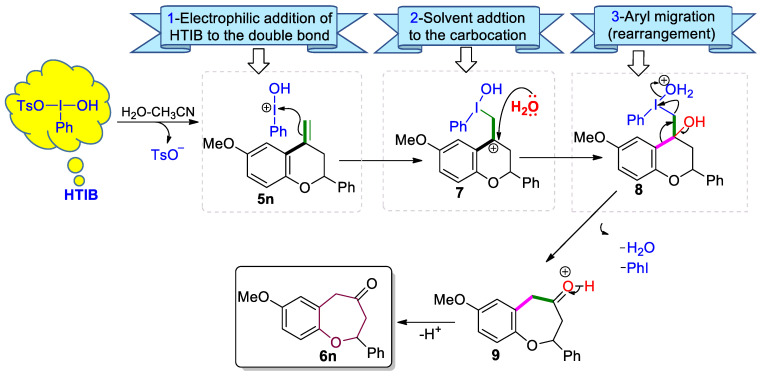
Proposed mechanism for ring expansion reactions.

The proposed mechanism for this rearrangement reaction involves the creation of carbocation **7** through an electrophilic attack by HTIB on *exo*-methylene. Following this, the solvent (H_2_O) undergoes a nucleophilic attack on the carbocation, forming intermediate **8.** The final ring expansion product is then produced through the migration of the aryl bond, accompanied by the elimination of phenyl iodide and H_2_O molecules (Scheme 6).

In the succeeding study, the pyrrole derivative was protected with electron-withdrawing (such as mesyl- and boc-) as well as electron-donating protecting groups (TBS- and benzyl-) to explore the impact of alkene stability on the resulting products. In this context, the mesylated pyrrole **4o** underwent successful transformation into the corresponding exocyclic alkene **5o** with a 50% yield (entry 1, Table 4) contrary to the **4i** conversion to **5i** where deprotection was also seen with the olefinic product (entry 10, Table 3). Similarly, Boc-protected pyrrole **4p** was treated under the above reaction conditions, leading to desired product **5p** with a moderate (56%) yield (entry 2, Table 4). TBS-protected pyrrole **4q** and benzyl-protected pyrrole **4r** were also treated with a Wittig reagent. Upon completion of these reactions (monitored via TLC), followed by work-up processes, the resultant olefins were decomposed before their successful isolation (entries 3 and 4).

As shown in Table 3 (entries 3 and 4), both furans underwent the Wittig olefinations and their resultant alkenes were isolated successfully. However, when alkyl-substituted furan **4s** underwent Wittig olefination (entry 5), the expected (hetero)benzylic alkenes were not isolated. Similarly, the alkyl substituted-pyrrole **4t** was also decomposed after Wittig olefination (entry 6). We also attempted to protect *N*-pyrrole **3w** with methyl and phenyl to compare the stability of the resulting olefinic products. However, the exocyclic alkenes of these pyrroles decomposed during their purification process (entries 7 and 8). Using the given protocol of Wittig olefination, pyrrole **5l** did not react due to the steric hindrance of the two-methyl groups located at the alpha position to carbonyl. The end of the reaction resulted in the recovery of the starting material in 96% yield (entry 9).

## 3. Experimental Procedures

### 3.1. General Information

The compounds outlined in the study underwent characterization involving ^1^H NMR, ^13^C NMR, and melting point analysis (for solids), with comparisons made against the available literature data. Additionally, all newly synthesized compounds were subjected to a comprehensive characterization process that included ^1^H NMR, ^13^C NMR, FTIR, HRMS and melting point determination (for solids). All protection reactions and Wittig reactions were executed within septum-sealed flasks under a N_2_ atmosphere. The progression of these reactions was continuously monitored through TLC analysis. Thin-layer chromatographic (TLC) analyses utilized silica gel plates of Merck Type 60 F254 on aluminum, with detection facilitated by UV light (254 nm). Staining was carried out using KMnO_4_ solution, phosphomolybdic acid solution, KmnO_4_ solution, vanillin or p-anisaldehyde. Purification via flash column chromatography was accomplished using either 200–400 mesh silica gel or Al_2_O_3_. Reagents and solvents underwent treatment and/or drying, as necessary, employing standard procedures.

The sample was analyzed and characterized using

- Buchi B-545 apparatus for melting point (Merck, Darmstadt, Germany);
- Perkin Elmer 1750-FT equipment for IR analysis (Perkin Elmer, Waltham, MA, USA);
- Bruker Daltonics microTOF electrospray for HRMS (Bruker, Billerica, MA, USA);
- Perkin Elmer 2400 Series II for elemental analysis (Perkin Elmer, Waltham, MA, USA);
- INOVA 300 MHz, (^1^H and ^13^C NMR analysis) (INOVA, Purcellville, VA, USA);
- Bruker AIII 300 MHz (^1^H and ^13^C NMR analysis) (Bruker, Billerica, MA, USA); and
- Bruker AIII 500 MHz (^1^H and ^13^C NMR analysis) (Bruker, Billerica, MA, USA).

Chemical shifts are expressed in parts per million (ppm), and coupling constants (*J*) are provided in hertz. Standard and peak multiplicities are denoted as follows: s for singlet, d for doublet, dd for doublet of doublets, t for triplet, q for quintet, br s for broad singlet and m for multiplet. The samples were prepared using CDCl_3_ as a solvent.

### 3.2. Synthesis of Isoquinolin Derivatives

6,7-Dihydroisoquinolin-8(5*H*)-one (**4k**) and 7,8-dihydroisoquinolin-5(6*H*)-one (**4j**).

To a stirred solution of **3k** (1.3 g, 10.0 mmol) in acetone (20 mL), MgSO_4_·7H_2_O (5.9 g, 24.0 mmol, 2.4 equiv) and H_2_O (10 mL) were added at 0 °C. To this mixture, KMnO_4_ (7.9 g, 50.0 mmol, 5.0 equiv) was added in small portions over 30–40 min and stirred further for 5 h at rt. The solid was filtered and the filtrate was treated with a saturated solution of K_2_S_2_O_5_. The resulting mixture was again filtered, and the filtrate was extracted with DCM (3 × 10 mL). The combined extract was washed with distilled water, saturated brine and dried over anhydrous MgSO_4_. The solvent was removed under vacuum pressure. The residue was purified by flash column chromatography (30–50% EtOAc in hexanes).

### 3.3. Conversion of Furan Derivatives to Pyrrole Derivatives

A sealed tube (volume 10 mL) was charged with furan derivatives, using alkyl amine or aryl amine as a solvent (3 mL) with *p*-TsOH (few crystals). The tube was sealed and placed in an oil bath at 125–160 °C for 20–24 h. After cooling to rt, the solution was brought to pH 1 by the addition of 1.0 M HCl solution. The aqueous mixture was then extracted with CH_2_Cl_2_. The organic layers were combined and dried over anhydrous MgSO_4_, and solvents were evaporated under reduced pressure.

### 3.4. General Protocol for the Protection of Heterocyclic Derivatives

To a round bottom flask, substrate (1.0 equiv) and NaH (1.5 equiv, 60% dispersion in mineral oil) were added to the solvent (generally THF) at 0 °C. The electrophile (protecting groups) (1.5 equiv) was added to the reaction mixture after 30 min. The reaction mixture was then brought to room temperature and stirred until the starting material was consumed (analyzed via TLC). After completion of the reaction, the mixture was quenched with distilled H_2_O and extracted with EtOAc. The organic layer was washed with brine, dried over anhydrous MgSO_4_ and filtered. The solvent was removed under reduced pressure. The crude product was purified using flash column chromatography.

### 3.5. General Protocol for Ring Expansion

A round bottom flask was charged with an alkene (1.0 equiv) in 90% CH_3_CN solution (CH_3_CN:H_2_O, 9:1, *v*/*v*). To the above reaction mixture, HTIB (1.2 equiv) was added and stirred for 5 min at room temperature. The reaction was monitored via TLC for the consumption of the starting material. After completion of the reaction, the mixture was quenched with saturated solution of NaHCO_3_, extracted with DCM. The combined organic extracts were washed with brine, dried over MgSO_4_ and filtered. The solvent was removed under reduced pressure. The residue was purified using flash column chromatography.

### 3.6. General Procedure for Wittig Olefination

Under a nitrogen atmosphere, 1.5 equiv of *t*-BuOK was added to a stirred mixture of 1.5 equiv of Ph_3_PCH_3_Br (pre-dried in a vacuum oven for 5–6 h) in anhydrous Et_2_O. The resulting canary yellow mixture was allowed to stir for 2 h. After which 1.0 equiv of the desired substrate dissolved in anhydrous Et_2_O/THF was added dropwise to the above reaction mixture. The reaction was monitored by TLC for complete consumption of the starting material. The reaction mixture was quenched with distilled H_2_O and extracted with EtOAc (3 × 10 mL). The organic layers were washed with brine, dried over MgSO_4_ and filtered. The solvent was removed under reduced pressure. The crude product was purified using flash column chromatography.

4-Methylene-4,5,6,7-tetrahydrobenzo[*b*]thiophene (**5a**) [23].

The reaction was performed following the general protocol, using *t*-BuOK (0.336 g, 3.0 mmol), Ph_3_PCH_3_Br (1.07 g, 3.0 mmol) and **4a** (0.304 g, 2.0 mmol) in anhydrous Et_2_O (12 mL).

Purification: The residue was purified by flash column chromatography (20–25% EtOAc in hexane in hexanes).

Yield: 97% (0.29 g, 1.94 mmol).

Sample appearance: Colorless oil.

^1^H NMR (200 MHz, CDCl_3_) δ: 1.86-195 (2H, q, *J* = 6.1 Hz), 2.46 (2H, t, *J* = 6. Hz), 2.83 (2H, t, *J* = 6 Hz), 4.84 (1H, s), 5.2 (1H, s), 7.00 (1H, d, *J* = 5.2 Hz), 7.14 (1H, d, *J* = 5.2 Hz).

^13^C NMR (50 MHz, CDCl_3_) δ: 24.4, 25.7, 31.8, 106.4, 122.2, 123.7, 135.8, 138.9, 140.1. 

5-Methylene-5,6,7,8-tetrahydro-4*H*-cyclohepta[*b*]thiophene (**5aa**).

The reaction was performed following the general protocol, using *t*-BuOK (0.336 g, 3.0 mmol), Ph_3_PCH_3_Br (1.07 g, 3.0 mmol) and **4aa** (0.332 g, 2.0 mmol) in anhydrous Et_2_O (10 mL).

Purification: The residue was purified by flash column chromatography (20–25% EtOAc in hexane in hexanes).

Yield: 69% (0.226 g, 1.38 mmol). 

Sample appearance: Light yellow oil. 

^1^H NMR (300 MHz, CDCl_3_) δ: 1.78–1.85 (2H, q, *J* = 6.0 Hz), 2.49 (2H, t, *J* = 6.1 Hz), 2.87 (2H, t, *J* = 5.7 Hz), 3.39 (2H, s), 4.72 (1H, s), 4.75 (1H, s), 6.76 (1H, d, *J* = 5.1 Hz), 6.88 (1H, d, *J* = 5.1 Hz). 

^13^C NMR (75 MHz, CDCl_3_) δ: 28.7, 28.8, 38.5, 39.6, 111.7, 120.4, 129.7, 137.1, 138.2, 147.1.

HRMS [ESI(+)] calcd. for [C_10_H_12_S+H]^+^ 165.0738, found 165.0743.

IR (film): 3396, 3145, 3119, 3081, 2934, 2895, 2847, 1648, 1439, 1349, 1326, 1253, 1110, 976, 849, 723, 705, 630 cm^−1^. 

4-Methylene-4,5,6,7-tetrahydrobenzofuran (**5b**) [24].

The reaction was performed following the general protocol, using *t*-BuOK (0.84 g, 7.5 mmol), Ph_3_PCH_3_Br (2.68 g, 7.5 mmol) and **4b** (0.68 g, 5.0 mmol) in anhydrous Et_2_O (15 mL). The crude reaction mixture was purified by flash column chromatography (10% EtOAc in hexanes).

Purification: The residue was purified by flash column chromatography (10% EtOAc in hexanes).

Yield: 86% (0.576 g, 4.3 mmol). 

Sample appearance: Colorless oil. 

^1^H NMR (300 MHz, CDCl_3_) δ: 1.55-1.94 (2H, q, *J* = 6.2), 2.41 (2H, t, *J* = 6.3 Hz), 2.68 (2H, t, *J* = 6.3 Hz), 4.76 (1H, s), 4.98 (1H, s), 6.47 (1H, d, *J* = 2 Hz), 7.24 (1H, d, *J* = 2 Hz).

^13^C NMR (75 MHz, CDCl_3_) δ: 23.5, 31.5, 104.9, 106.50, 119.3, 138.6, 141.3, 153.4. 

6,6-Dimethyl-4-methylene-4,5,6,7-tetrahydrobenzofuran (**5c**).

The reaction was performed following the general protocol, using *t*-BuOK (0.84 g, 7.5 mmol), Ph_3_PCH_3_Br (2.68 g, 7.5 mmol) and **4c** (0.82 g, 5.0 mmol) in anhydrous Et_2_O (15 mL). 

Purification: The residue was purified by flash column chromatography (10% EtOAc in hexanes).

Yield: 48% (0.388 g, 2.4 mmol). 

Sample appearance: Colorless oil. 

^1^H NMR (300 MHz, CDCl_3_) δ: 1.00 (6H, s), 2.18 (2H, s), 2.48 (2H, s), 4.79 (1H, s), 5.04 (1H, s), 6.47 (1H, d, *J* = 2.0 Hz), 7.25 (1H, d, *J* = 2.0 Hz).

^13^C NMR (75 MHz, CDCl_3_) δ: 28.33, 32.7, 37.4, 45.7, 106.3, 106.4, 118.4, 137.5, 141.5, 153.0.

Elemental Analysis: calcd. for [C, 81.44; H, 8.70] found [C, 81.17; H, 8.28].

IR (film): 3067, 2929, 2836, 1583, 1462, 1462, 1437, 1327, 1252, 1073, 726, 719 cm^−1^. 

1,2,3,9-Tetrahydro-*4H*-carbazol-4-one (**3d**) [29,30].

The reaction was performed following the general protocol, using *t*-BuOK (0.37 g, 3.3 mmol), Ph_3_PCH_3_Br (1.18 g, 3.3 mmol) and **4d** (0.5 g, 2.2 mmol) in anhydrous Et_2_O (12 mL). 

Purification: The residue was purified by flash column chromatography (40–50% EtOAc in hexanes).

Yield: 83% (0.337 g, 1.8 mmol). 

Sample appearance: Light brown solid. 

Milting Point: 225–227 °C (lit 223 °C).

^1^H NMR (300 MHz, MeOD) δ: 2.10 (2H, q, *J* = 6.2 Hz), 2.43 (2H, t, *J* = 6.4 Hz), 2.95 (2H, t, *J* = 6.1 Hz), 7.14–7.18 (2H, m), 7.41 (1H, d, *J* = 6.6 Hz), 7.98 (1H, d, *J* = 7.8 Hz), 11.86 (1H, s).

^13^C NMR (75 MHz, MeOD) δ: 24.3, 25.2, 39.0, 112.6, 113.7, 122.1, 123.3, 124.3, 126.3, 138.0, 155.1, 197.3.

2,2-Mimethyl-1,2,3,9-tetrahydro-4*H*-carbazol-4-one (**3e**) [35].

The reaction was performed following the general protocol, using *t*-BuOK (0.168 g, 1.5 mmol), Ph_3_PCH_3_Br (0.535 g, 1.5 mmol) and **4e** (0.255 g, 1.0 mmol) in anhydrous Et_2_O (8 mL). 

Purification: The residue was purified by flash column chromatography (40–50% EtOAc in hexane in hexanes).

Yield: 66% (0.139 g, 0.66 mmol). 

Sample appearance: Brown solid

Milting Point: 207 °C (lit 209–210 °C).

^1^H NMR (300 MHz, CDCl_3_) δ: 1.16 (6H, s), 2.46 (2H, s), 2.83 (2H, s), 7.21–7.27 (2H, m), 7.35 (1H, d, *J* = 8.7 Hz), 8.20 (1H, d, *J* = 8.7 Hz), 8.91 (1H, s).

^13^C NMR (75 MHz, CDCl_3_) δ: 28.8, 35.9, 37.5, 52.5, 111.1, 112.3, 121.5, 122.6, 123.3, 124.8, 136.1, 150.4, 193.9.

4-Methylene-9-tosyl-2,3,4,9-tetrahydro-1*H*-carbazole (**5f**) [23].

The reaction was performed following the general protocol, using *t*-BuOK (0.101 g, 0.9 mmol), (Ph_3_PCH_3_Br (0.321 g, 0.9 mmol), and **4f** (0.17 g, 0.5 mmol) in anhydrous Et_2_O (8 mL).

Purification: The residue was purified by flash column chromatography (5% EtOAc in hexane in hexanes).

Yield: 82% (0.138 g, 0.41 mmol). 

Sample appearance: Light yellow oil. 

^1^H NMR (300 MHz, CDCl_3_) δ: 1.92–2.00 (2H, q, *J* = 6.2 Hz), 2.33 (3H, s), 2.45 (2H, t, *J* = 6.1 Hz), 3.15 (2H, t, *J* = 6.3 Hz), 5.05 (1H, s), 5.50 (1H, s), 7.19 (2H, d, *J* = 7.8 Hz), 7.25–7.30 (2H, m), 7.66 (2H, d, *J* = 8.4 Hz), 7.79 (1H, d, *J* = 9 Hz), 8.22 (1H, d, *J* = 9.3 Hz).

^13^C NMR (75 MHz, CDCl_3_) δ: 21.7, 23.8, 25.3, 33.2, 108.4, 114.7, 118.0, 120.4, 124.0, 124.3, 126.5, 127.8, 130.0, 136.3, 136.8, 138.3, 139.6, 144.9.

2,2-Dimethyl-4-methylene-9-tosyl-2,3,4,9-tetrahydro-1*H*-carbazole (**5g**) [23].

The reaction was performed following the general protocol, using *t*-BuOK (0.22 g, 1.96 mmol), Ph_3_PCH_3_Br (0.7 g, 1.96 mmol) and **4g** (0.3 g, 0.817 mmol) in anhydrous Et_2_O (15 mL).

Purification: The residue was purified by flash column chromatography (20% Et_2_O in hexane in hexanes).

Yield: 58% (0.173 g, 0.474 mmol). 

Sample appearance: Colorless oil. 

^1^H NMR (300 MHz, CDCl_3_) δ: 1.00 (6H, s), 2.22 (2H, s), 2.33 (3H, s), 2.96 (2H, s), 5.05 (1H, s), 5.56 (1H, s), 7.18 (2H, d, *J* = 8.1 Hz), 7.26–7.29 (2H, q, *J* = 3.4 Hz), 7.62 (2H, d, *J* = 8.1 Hz), 7.78–7.81 (1H, q, *J* = 3 Hz), 8.19–8.22 (1H, q, *J* = 3 Hz).

^13^C NMR (75 MHz, CDCl_3_) δ: 21.7, 28.37, 32.07, 39.17, 47.1, 109,7, 114.8, 117.5, 120.4, 124.0, 124.2, 126.5, 127.7, 130.0, 136.20, 137.20, 137,7, 138.3, 145.0. 

*t*-Butyl 1-methylene-1,2,3,4-tetrahydro-9*H*-carbazole-9-carboxylate (**5h**) [23].

The reaction was performed following the general protocol, using *t*-BuOK (0.099 g, 0.88 mmol), Ph_3_PMeBr (0.313 g, 0.88 mmol), and **6c** (0.125 g, 0.44 mmol) in anhydrous Et_2_O (6 mL).

Purification: The residue was purified by flash column chromatography (5% EtOAc in hexane in hexanes).

Yield: 91% (0.114 g, 0.4 mmol). 

Sample appearance: Yellow oil. 

^1^H NMR (300 MHz, CDCl_3_) δ: 1.55 (9H, s), 1.86–195 (2H, q, *J* = 6.2 Hz), 2.53 (2H, t, *J* = 6 Hz), 2.70 (2H, t, *J* = 6.3 Hz), 4.99 (1H, s), 5.05 (1H, s), 7.08–7.24 (2H, m), 7.33 (1H, d, *J* = 7.5 Hz), 7.87 (1H, d, *J* = 8.1 Hz).

^13^C NMR (75 MHz, CDCl_3_) δ: 21.5, 25.0, 28.1, 34.4, 83.5, 110.6, 114.6, 118.9, 122.2, 122.5, 125.0, 129

1-Methylene-2,3,4,9-tetrahydro-1*H*-carbazole (**5i**).

The reaction was performed following the general protocol, using *t*-BuOK (0.336 g, 3.0 mmol), Ph_3_PCH_3_Br (1.072 g, 3.0 mmol), in anhydrous Et_2_O (6 mL) and **4i** (0.526 g, 2.0 mmol) in DCM (4.0 mL). (DCM was used for the solubility of **4i**).

Purification: The residue was purified by flash column chromatography (15–20% EtOAc in hexane in hexanes).

Yield: 49% (0.179 g, 0.98 mmol). 

Sample appearance: Colorless oil. 

^1^H NMR (300 MHz, CDCl_3_) δ: 1.93-2.01 (2H, q, *J* = 6.1 Hz), 2.58 (2H, t, *J* = 6 Hz), 2.82 (2H, t, *J* = 6 Hz), 4.90 (1H, s), 5.09 (1H, s), 7.09 (1H, t, *J* = 7.5 Hz), 7.19 (1H, t, *J* = 7.5 Hz), 7.31 (1H, d, *J* = 8.1 Hz), 7.51 (1H, d, *J* = 7.8 Hz).

^13^C NMR (75 MHz, CDCl_3_) δ: 21.7, 24.5, 32.2, 103.6, 110.8, 115.2, 119.1, 119.6, 123.1, 127.8, 133.1, 136.7, 137.2. 

HRMS [ESI(+)] calcd. for [C_13_H_13_N+H]^+^ 184.1126, found 184.1121. 

IR (film): 3540, 3520, 3369, 3343, 3053, 3001, 2971, 2938, 1915, 1632, 1597,1470, 1452, 1372, 1172, 747, 669, 580, 542 cm^−1^.

5-Methylene-5,6,7,8-tetrahydroisoquinoline (**5j**).

The reaction was performed following the general protocol, using *t*-BuOK (0.336 g, 3.0 mmol), Ph_3_PCH_3_Br (1.07 g, 3.0 mmol) and **4j** (0.294 g, 2.0 mmol) in anhydrous Et_2_O (12 mL).

Purification: The residue was purified by flash column chromatography (30–40% EtOAc in hexane in hexanes).

Yield: 88% (0.254 g, 1.76 mmol). 

Sample appearance: Yellow oil. 

^1^H NMR (300 MHz, CDCl_3_) δ: 1.84–1.91 (2H, q, *J* = 6.2 Hz), 2.56 (2H, t, *J* = 6.3 Hz), 2.81 (2H, t, *J* = 6.3 Hz), 5.15 (1H, s), 5.68 (1H, s), 7.43 (1H, d, *J* = 5.6 Hz), 8.34 (1H, d, *J* = 5.6 Hz), 8.37 (1H, s).

^13^C NMR (75 MHz, CDCl_3_) δ: 23.1, 27,3, 32,4, 112.0, 117.9, 132.1, 141.3, 141.7, 147.2, 151.1.

HRMS [ESI(+)] calcd. for [C_10_H_11_N+H]^+^ 146.0970, found 146.0969. 

IR (film): 3401, 3087, 3057, 3034, 3019, 2983, 2937, 2863, 2839, 2677, 2488, 2422, 1894, 1800, 1619, 1630, 1592, 1547, 1487, 1456, 1440, 1432, 1413, 1341, 1330, 1308, 1292, 1272, 1248, 1177. 1147, 1104, 1064, 070, 902, 864, 831, 808, 768 cm^−1^.

8-Methylene-5,6,7,8-tetrahydroisoquinoline (**5k**).

The reaction was performed following the general protocol, using *t*-BuOK (0.336 g, 3.0 mmol), Ph_3_PCH_3_Br (1.07 g, 3.0 mmol) and **4k** (0.294 g, 2.0 mmol) in anhydrous Et_2_O (12 mL).

Purification: The residue was purified by flash column chromatography (30–40% EtOAc in hexane in hexanes).

Yield: 62% (0.179 g, 1.24 mmol). 

Sample appearance: Light yellow oil. 

^1^H NMR (300 MHz, CDCl_3_) δ: 1.83–1.92 (2H, q, *J* = 6.3 Hz), 2.53 (2H, t, *J* = 6.2 Hz), 2.80 (2H, t, *J* = 6.2 Hz), 5.03 (1H, s), 5.57 (1H, s), 6.98 (1H, d, *J* = 5.1 Hz), 8.30 (1H, d, *J* = 5.1 Hz), 8.83 (1H, s).

^13^C NMR (75 MHz, CDCl_3_) δ: 22.2, 29.6, 32.7, 109.1, 123.6, 130.8, 140.6, 145.6, 146.2, 147.7.

HRMS [ESI(+)] calcd. for [C_10_H_11_N+H]^+^ 146.0970, found 146.0966. 

IR (film): 3403, 3088, 3051, 3032, 3019, 2982, 2864, 2840, 2675, 2481, 2421, 1890, 1800, 1620, 1630, 1590, 1545, 1488, 1442, 1431, 1411, 1344, 1333, 1305, 1292, 1272, 1250, 1175. 1147, 1104, 1064, 070, 902, 865, 831, 808 cm^−1^.

4-Methylenethiochroman (**5l**) [36].

The reaction was performed following the general protocol, using *t*-BuOK (0.336 g, 3.0 mmol), Ph_3_PCH_3_Br (1.07 g, 3.0 mmol) and **4l** (0.328 g, 2.0 mmol) in anhydrous Et_2_O (12 mL).

Purification: The residue was purified by flash column chromatography (10% EtOAc in hexane in hexanes).

Yield: 72% (0.233 g, 1.44 mmol).

Sample appearance: Colorless oil.

^1^H NMR (300 MHz, CDCl_3_) δ: 2.83 (2H, t, *J* = 6.0 Hz), 3.06 (2H, t, *J* = 6.0 Hz), 4.94 (1H, s), 5.47 (1H, s), 6.98–7.05 (1H, m), 7.08–7.10 (2H, m), 7.52 (1H, d, *J* = 8.1 Hz).

^13^C NMR (75 MHz, CDCl_3_) δ: 27.8, 33.0, 111.7, 124.3, 126.4, 126.8, 128.2, 132.9, 133.5, 141.6. 

6-Fluoro-4-methylenechroman (**5m**) [23].

The reaction was performed following the general protocol, using *t*-BuOK (0.336 g, 3.0 mmol), Ph_3_PCH_3_Br (1.07 g, 3.0 mmol) and **4m** (0.328 g, 2.0 mmol) in anhydrous Et_2_O (12 mL). 

Purification: The residue was purified by flash column chromatography (10% EtOAc in hexane in hexanes).

Yield: 90% (0.295 g, 1.8 mmol). 

Sample appearance: Colorless oil. 

^1^H NMR (500 MHz, CDCl_3_) δ: 2.67 (2H, t, *J* = 5.7 Hz), 4.20 (2H, t, *J* = 5.7 Hz), 4.94 (1H, s), 5.47 (1H, s), 6.78 (1H, dd, *J* = 10.0 and 5.0 Hz), 6.84–6.89 (1H, td, *J* = 8.5 and 3.0 Hz), 7.23 (1H, dd, *J* = 9.5, and 3.0 Hz).

^13^C NMR (125 MHz, CDCl_3_) δ: 31.3, 66.9, 108.4, 110.2 (*J* _C-F_ = 13.8 Hz), 116.5 (*J*_C-F_ = 14.2 Hz), 118.6 (*J*_C-F_ = 13.8 Hz), 122.6 (*J*_C-F_ = 3.7 Hz), 136.5, 150.8, 156.3, 158.2.

4-Methylene-1-(methylsulfonyl)-4,5,6,7-tetrahydro-1*H*-indole (**5o**) [23].

The reaction was performed following the general protocol, using *t*-BuOK (0.336 g, 3.0 mmol), Ph_3_PCH_3_Br (1.07 g, 3.0 mmol) and **4o** (0.426 g, 2.0 mmol) in anhydrous Et_2_O (6 mL). 

Purification: The residue was purified by flash column chromatography (25% EtOAc in hexane in hexanes).

Yield: 50% (0.21 g, 1.0 mmol). 

Sample appearance: Yellow viscous oil

^1^H NMR (300 MHz, CDCl_3_) δ: 1.88–1.96 (2H, q, *J* = 6.2 Hz), 2.42 (2H, t, *J* = 6.3 Hz), 2.87 (2H, t, *J* = 6.1 Hz), 3.10 (3H, s), 4.83 (1H, s), 5.10 (1H, s), 6.44 (1H, d, *J* = 3.3 Hz), 7.03 (1H, d, *J* = 3.3 Hz).

^13^C NMR (75 MHz, CDCl_3_) δ: 23.4, 23.6, 31.2, 42.7, 106.0, 108.2, 121.3, 124.7, 130.9, 138.6.

*t*-Butyl 4-methylene-4,5,6,7-tetrahydro-1*H*-indole-1-carboxylate (**5p**) [24].

The reaction was performed following the general protocol **2.1**, using *t*-BuOK (0.302 g, 2.7 mmol), Ph_3_PCH_3_Br (0.944 g, 2.7 mmol) and **4p** (0.424 g, 1.8 mmol) in anhydrous Et_2_O (12 mL).

Purification: The residue was purified by flash column chromatography (33% EtOAc in hexane in hexanes).

Yield: 58% (0.243 g, 1.04 mmol). 

Sample appearance: Colorless oil. 

^1^H NMR (300 MHz, CDCl_3_) δ: 1.58 (9H, s), 1.83–191 (2H, q, *J* = 6.1 Hz), 2.39 (2H, t, *J* = 6.1 Hz), 2.93 (2H, t, *J* = 6.3 Hz), 4.74 (1H, s), 5.04 (1H, s), 6.32 (1H, d, *J* = 3.6 Hz), 7.14 (1H, d, *J* = 3.6 Hz).

^13^C NMR (75 MHz, CDCl_3_) δ: 24.0, 25.0, 28.2, 31.5, 83.5, 104.5, 106.9, 120.7, 123.5, 131.9, 139.7, 149.6.

## 4. Conclusions

In summary, this study focused on understanding how common protective groups and heterocycles affect the (hetero)benzylic positions in the resultant alkenes produced through the widely used Wittig olefination reaction. The results described in this study showed that comparatively less reactive heterocyclic derivatives or electron-withdrawing groups (EWGs) connected to *N*-(hetero)aryls improved the stability of the benzylic position, both during and after Wittig olefination, leading to the creation of stable alkenes. Conversely, more reactive heterocyclic derivatives or electron-donating groups (EDGs) connected to *N*-(hetero)aryls improved the reactivity of the benzylic position, leading to the creation of unstable products that resulted in lower yields or decomposition of the alkenes.

## Figures and Tables

**Table 1:** Preparation of pyrrole derivatives.

Entry	Substrate	Conditions	Product	Yield %
1	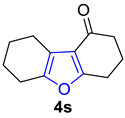	CH_3_NH_2_, PTSA (few crystals) rt to 125 °C, 20 h	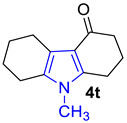	25%
2	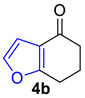	CH_3_NH_2_, PTSA (few crystals) rt to 125 °C, 20 h	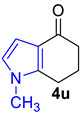	98%
3	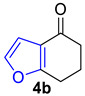	5.0 equiv *m*-chloroaniline xylene, PTSA rt to 160 °C, 24 h	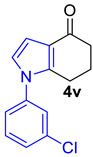	73%
4	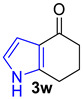	1.0 equiv *n*-BuLi, 1.0 equiv MeI THF, 0 °C to rt, 1 h	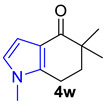	11%

**Table 2:** Protection of indole and pyrrole derivatives.

Entry	Substrate	Conditions	Product	%Yield
1	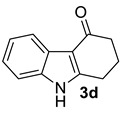	(i) 1.5 equiv NaH, THF, 0 °C, 30 min (ii) 1.5 equiv AcCl, 0 °C to rt, 3 h	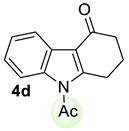	92
2	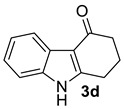	(i) 1.5 equiv NaH, THF, 0 °C, 30 min (ii) 1.5 equiv TsCl, 0 °C to rt, 2 h	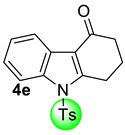	62
3	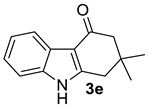	(i) 1.5 equiv NaH, DMF, 0 °C, 30 min (ii) 1.5 equiv AcCl, 0 °C to rt, 3 h	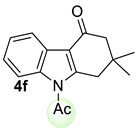	84
4	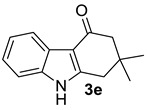	(i) 1.5 equiv NaH, THF, 0 °C, 30 min (ii) 1.5 equiv TsCl, 0 °C to rt, 2 h	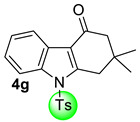	72
5	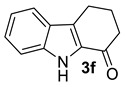	(i) 3.0 equiv NaH, THF, 0 °C, 30 min (ii) 3.0 equiv Boc_2_O, 0 °C to rt, 6 h	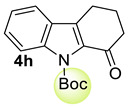	67
6	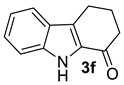	(i) 3.0 equiv NaOH, DCE, 0 ºC, 30 min (ii) 3.0 equiv MsCl, 0 ºC to rt, 24 h	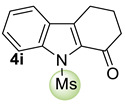	64
7	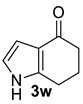	(i) 3.0 equiv NaOH, DCE, 0 °C, 30 min (ii) 3.0 equiv MsCl, 0 °C to rt, 15 h	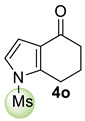	81
8	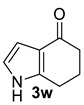	(i) 1.5 equiv NaH, THF, 0 °C, 30 min (ii) 1.5 equiv Boc_2_O, 0 °C to rt, 3 h	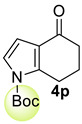	98
9	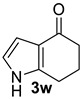	(i) 1.5 equiv NaH, THF, 0 °C, 30 min (ii) 1.5 equiv TBS, 0 °C to rt, 20 h	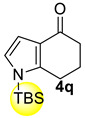	75
10	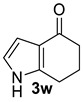	(i) 4.0 equiv KOH, DMSO, 0 °C, 30 min (ii) 1.2 equiv PhCH_2_Br, 0 °C to rt, 12 h	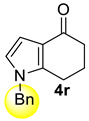	90

**Table 3:** Wittig olefination of heterocyclic ketones.

Entry	Substrate	Conditions	Product	Yield
1	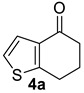	1.5 equiv *t*-BuOK 1.5 equiv Ph_3_PCH_3_Br Et_2_O, rt, 3 h	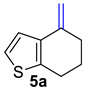	97%
2	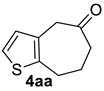	1.5 equiv *t*-BuOK 1.5 equiv Ph_3_PCH_3_Br Et_2_O, rt, 3 h	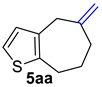	69%
3	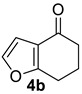	1.5 equiv *t*-BuOK 1.5 equiv Ph_3_PCH_3_Br Et_2_O, rt, 3 h	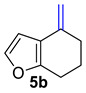	86%
4	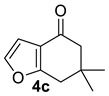	1.5 equiv *t*-BuOK 1.5 equiv Ph_3_PCH_3_Br Et_2_O, rt, 3 h	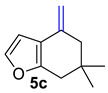	48%
5	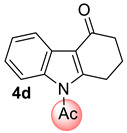	1.5 equiv *t*-BuOK 1.5 equiv Ph_3_PCH_2_Br Et_2_O, rt, 3 h	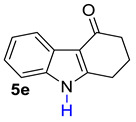	83%
6	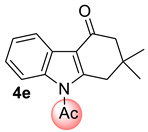	1.5 equiv *t*-BuOK 1.5 equiv Ph_3_PCH_3_Br Et_2_O, rt, 3 h	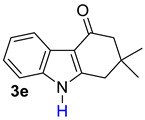	66%
7	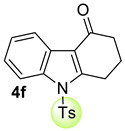	1.8 equiv *t*-BuOK 1.8 equiv Ph_3_PCH_3_Br Et_2_O, rt, 10 h	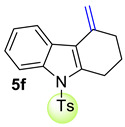	82%
8	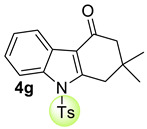	2.4 equiv *t*-BuOK 2.4 equiv Ph_3_PCH_3_Br Et_2_O, rt, 26 h	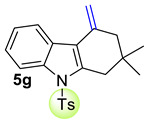	58%
9	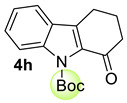	2.0 equiv *t*-BuOK 2.0 equiv Ph_3_PCH_3_Br Et_2_O, rt, 3 h	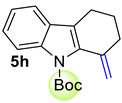	91%
10	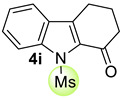	1.5 equiv *t*-BuOK 1.5 equiv Ph_3_PCH_3_Br Et_2_O:DCM (4:1), rt, 15 h	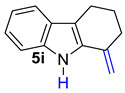	49%
11	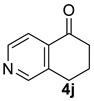	1.5 equiv *t*-BuOK 1.5 equiv Ph_3_PCH_3_Br Et_2_O, rt, 3 h	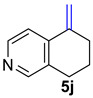	88%
12	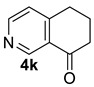	1.5 equiv *t*-BuOK 1.5 equiv Ph_3_PCH_3_Br Et_2_O, rt, 3 h	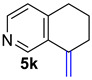	62%
13	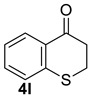	1.5 equiv *t*-BuOK 1.5 equiv Ph_3_PCH_3_Br Et_2_O, rt, 5 h	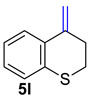	72%
14	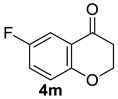	1.5 equiv *t*-BuOK 1.5 equiv Ph_3_PCH_3_Br Et_2_O, rt, 5 h	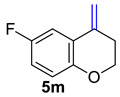	90%
15	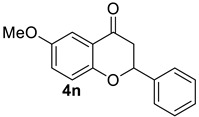	1.5 equiv *t*-BuOK 1.5 equiv Ph_3_PCH_3_Br Et_2_O, rt, 5 h	decomposed (complex mixture)	

**Table 4:** Wittig olefination of heterocyclic ketones.

Entry	Substrate	Conditions	Product	
1	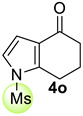	1.5 equiv *t*-BuOK 1.5 equiv Ph_3_PCH_3_Br Et_2_O, rt, 24 h	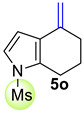	50%
2	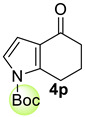	1.5 equiv *t*-BuOK 1.5 equiv Ph_3_PCH_3_Br Et_2_O, rt, 6 h	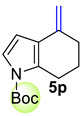	58%
3	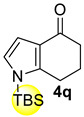	1.5 equiv *t*-BuOK 1.5 equiv Ph_3_PCH_3_Br Et_2_O, rt, 5 h	Decomposed (complex mixture)	
4	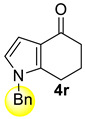	1.5 equiv *t*-BuOK 1.5 equiv Ph_3_PCH_3_Br Et_2_O, rt, 5 h	Decomposed (complex mixture)	
5	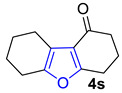	1.5 equiv *t*-BuOK 1.5 equiv Ph_3_PCH_3_Br Et_2_O, rt, 3 h	Decomposed (complex mixture)	
6	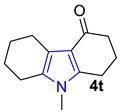	1.5 equiv *t*-BuOK 1.5 equiv Ph_3_PCH_3_Br Et_2_O, rt, 10 h	Decomposed (complex mixture)	
7	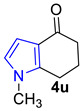	1.5 equiv *t*-BuOK 1.5 equiv Ph_3_PCH_3_Br Et_2_O, rt, 12 h	Decomposed (complex mixture)	
8	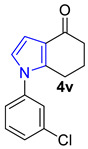	1.5 equiv *t*-BuOK 1.5 equiv Ph_3_PCH_3_Br Et_2_O, rt, 10 h	Decomposed (complex mixture)	
9	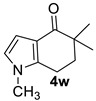	1.5 equiv *t*-BuOK 1.5 equiv Ph_3_PCH_3_Br Et_2_O, rt, 10 h	No reaction	

Note: The decomposition of these compounds could be observed by TLC analysis where the single spot converted to multiple spots during the work-up and purification processes.

## Data Availability

Data are contained within the article and Supplementary Materials.

## Supplementary Materials

The following supporting information can be downloaded at: https://www.mdpi.com/article/10.3390/molecules29020501/s1, The supporting information (SI) includes substrate preparation, procedures and analytical data (1H NMR, 13C NMR) for all new compounds and selected ones. Refs. [37,38,39,40,41,42,43,44,45] are cited in the Supplementary Materials.

## References

[1] Al-Mulla A. (2017). A Review: Biological Importance of Heterocyclic Compounds. Der Pharma Chem..

[2] Arora P., Arora V., Lamba H.S., Wadhwa D. (2012). Importance of Heterocyclic Chemistry: A Review. Int. J. Pharm. Sci. Res..

[3] Alvarez-Builla J., Barluenga J. (2011). Heterocyclic Compounds: An Introduction.

[4] Kabir E., Uzzaman M. (2022). A Review on Biological and Medicinal Impact of Heterocyclic Compounds. Results Chem..

[5] Chinchilla R., Nájera C., Yus M. (2004). Metalated Heterocycles and Their Applications in Synthetic Organic Chemistry. Chem. Rev..

[6] Karanam P., Reddy G.M., Lin W. (2018). Strategic Exploitation of the Wittig Reaction: Facile Synthesis of Heteroaromatics and Multifunctional Olefins. Synlett.

[7] Das U., Tsai Y.-L., Lin W. (2014). Preparation of Functionalized Heteroaromatics Using an Intramolecular Wittig Reaction. Org. Biomol. Chem..

[8] Rocha D.H.A., Pinto D.C.G.A., Silva A.M.S. (2018). Applications of the Wittig Reaction on the Synthesis of Natural and Natural-Analogue Heterocyclic Compounds. Eur. J. Org. Chem..

[9] Heravi M.M., Zadsirjan V., Hamidi H., Daraie M., Momeni T. (2020). Recent Applications of the Wittig Reaction in Alkaloid Synthesis. Alkaloids Chem. Biol..

[10] Hoffmann R.W. (2001). Wittig and His Accomplishments: Still Relevant beyond His 100th Birthday. Angew. Chem. Int. Ed..

[11] Byrne P.A., Gilheany D.G. (2013). The Modern Interpretation of the Wittig Reaction Mechanism. Chem. Soc. Rev..

[12] Maryanoff B.E., Reitz A.B. (1989). The Wittig Olefination Reaction and Modifications Involving Phosphoryl-Stabilized Carbanions. Stereochemistry, Mechanism, and Selected Synthetic Aspects. Chem. Rev..

[13] Edmonds M., Abell A. (2004). The Wittig Reaction. Modern Carbonyl Olefination.

[14] Nicolaou K.C., Härter M.W., Gunzner J.L., Nadin A. (1997). The Wittig and Related Reactions in Natural Product Synthesis. Liebigs Ann..

[15] Takeda T. (2006). Modern Carbonyl Olefination: Methods and Applications.

[16] Heravi M.M., Ghanbarian M., Zadsirjan V., Alimadadi Jani B. (2019). Recent Advances in the Applications of Wittig Reaction in the Total Synthesis of Natural Products Containing Lactone, Pyrone, and Lactam as a Scaffold. Monatshefte Chem. Chem. Mon..

[17] Khaskin E., Milstein D. (2015). Catalytic, Oxidant-Free, Direct Olefination of Alcohols Using Wittig Reagents. Chem. Commun..

[18] Tukhtaev H.B., Ivanov K.L., Bezzubov S.I., Cheshkov D.A., Melnikov M.Y., Budynina E.M. (2019). Aza-Wittig Reaction with Nitriles: How Carbonyl Function Switches from Reacting to Activating. Org. Lett..

[19] Fitjer L., Quabeck U. (1985). The Wittig Reaction Using Potassium-Tert-Butoxide High Yield Methylenations of Sterically Hindered Ketones. Synth. Commun..

[20] Rathke M.W., Nowak M. (1985). The Horner-Wadsworth-Emmons Modification of the Wittig Reaction Using Triethylamine and Lithium or Magnesium Salts. J. Org. Chem..

[21] Taylor R.J.K., Reid M., Foot J., Raw S.A. (2005). Tandem Oxidation Processes Using Manganese Dioxide: Discovery, Applications, and Current Studies. Acc. Chem. Res..

[22] Jeena V., Robinson R.S. (2014). Recent Developments in One-Pot Tandem Oxidation Process Coupling Reactions. RSC Adv..

[23] Khan A., Silva L.F., Rabnawaz M. (2021). Iodine (III)-Promoted Ring Expansion Reactions: A Metal-Free Approach toward Seven-Membered Heterocyclic Rings. Asian J. Org. Chem..

[24] Lussari N., Khan A., Pilli R.A., Dos Santos A.A., Silva L.F., Braga A.A.C. (2022). A DFT Study on the Formation of Heterocycles via Iodine (Iii)-Promoted Ring Expansion Reactions. New J. Chem..

[25] Plouvier B., Beatch G.N., Jung G.L., Zolotoy A., Sheng T., Clohs L., Barrett T.D., Fedida D., Wang W.Q., Zhu J.J. (2007). Synthesis and Biological Studies of Novel 2-Aminoalkylethers as Potential Antiarrhythmic Agents for the Conversion of Atrial Fibrillation. J. Med. Chem..

[26] Lee Y.R., Morehead A.T. (1995). A New Route for the Synthesis of Furanoflavone and Furanochalcone Natural Products. Tetrahedron.

[27] Peng Y., Luo J., Feng Q., Tang Q. (2016). Understanding the Scope of Feist–Bénary Furan Synthesis: Chemoselectivity and Diastereoselectivity of the Reaction Between α-Halo Ketones and β-Dicarbonyl Compounds. Eur. J. Org. Chem..

[28] Arai M., Miyauchi Y., Miyahara T., Ishikawa T., Saito S. (2008). Synthesis of 4-Acetoxyindoles and Related Derivatives by Means of Air Oxidation of 4-Oxo-4,5,6,7-Tetrahydroindoles Obtained from Nitroalkenes and Cyclohexane-1,3-Diones. Synlett.

[29] de Candia M., Zaetta G., Denora N., Tricarico D., Majellaro M., Cellamare S., Altomare C.D. (2017). New Azepino[4,3-b]Indole Derivatives as Nanomolar Selective Inhibitors of Human Butyrylcholinesterase Showing Protective Effects against NMDA-Induced Neurotoxicity. Eur. J. Med. Chem..

[30] Kadam S.A., Haav K., Toom L., Haljasorg T., Leito I. (2014). NMR Method for Simultaneous Host-Guest Binding Constant Measurement. J. Org. Chem..

[31] Azizi N., Khajeh-Amiri A., Ghafuri H., Bolourtchian M., Saidi M. (2009). Iron-Catalyzed Inexpensive and Practical Synthesis of N-Substituted Pyrroles in Water. Synlett.

[32] Ueda K., Amaike K., Maceiczyk R.M., Itami K., Yamaguchi J. (2014). β-Selective C-H Arylation of Pyrroles Leading to Concise Syntheses of Lamellarins C and I. J. Am. Chem. Soc..

[33] Lardenois P., Frost J., Dargazanli G., George P. (1996). A Convenient Synthesis 7,8-Dihydroisoquinolin-5(6H)-One. Synth. Commun..

[34] Khan A., Silva L.F., Rabnawaz M. (2021). A Comparative Study of Thallium (Iii) and Iodine (Iii)-Mediated Ring Contraction Reactions for the Synthesis of Indane. New J. Chem..

[35] Zuo Y., He X., Ning Y., Wu Y., Shang Y. (2017). Rh(III)-Catalyzed C–H Activation/Intramolecular Cyclization: Access to N-Acyl-2,3-Dihydro-1 H-Carbazol-4(9H)-Ones from Cyclic 2-Diazo-1,3-Diketones and N-Arylamides. ACS Omega.

[36] Gabbutt C.D., Hepworth J.D., Heron B.M. (1995). Reactions of Some 2H-Chromenes and 2H-Thiochromenes with Triazolinediones. Tetrahedron.

[37] Davies H.M.L., Manning J.R. (2006). C-H Activation as a Strategic Reaction: Enantioselective Synthesis of 4-Substituted Indoles. J. Am. Chem. Soc..

[38] Cho H., Iwama Y., Sugimoto K., Mori S., Tokuyama H. (2010). Regioselective Synthesis of Heterocycles Containing Nitrogen Neighboring an Aromatic Ring by Reductive Ring Expansion Using Diisobutylaluminum Hydride and Studies. J. Org. Chem.

[39] Bardakos V., Sucrow W. (1978). Enhydrazine, 21: Lactame Aus 1,2,3,9-Tetrahydro-4H-Carbazol-4-Onen. Chem. Ber..

[40] Montalban A.G., Baum S.M., Cowell J., McKillop A. (2012). Formation of N-Substituted 4-and 7-Oxo-4, 5, 6, 7-Tetrahydroindoles Revisited: A Mechanistic Interpretation and Conversion into 4-and 7-Oxoindoles. Tetrahedron Lett..

[41] Laurila M.L., Magnus N.A., Staszak M.A. (2009). Green N-Methylation of Electron Deficient Pyrroles with Dimethylcarbonate. Org. Process Res. Dev..

[42] Lee I.-S.H. (2012). Synthesis of N-Aryl-4,5,6,7-Tetrahydroindoles. Korean Chem. Soc..

[43] McEachern E.J., Yang W., Chen G., Skerlj R.T., Bridger G.J. (2003). Convenient Synthesis of 5, 6, 7, 8-Tetrahydroquinolin-8-Ylamine and 6, 7-Dihydro-5 H-Quinolin-8-One. Synth. Commun..

[44] Gartshore C.J., Lupton D.W. (2013). Studies on the Enantioselective Synthesis of Carbazolones as Intermediates in Aspidosperma and Kopsia Alkaloid Synthesis. Aust. J. Chem..

[45] Piras L., Ghiron C., Minetto G., Taddei M. (2008). Microwave-Assisted Synthesis of Tetrahydroindoles. Tetrahedron Lett..

