# Supplementary material for: Reactivity and Stability of (Hetero)Benzylic Alkenes via the Wittig Olefination Reaction
Source: Molecules. 2024 Jan 19;29(2):501. doi: 10.3390/molecules29020501 (PMC10819551; doi:10.3390/molecules29020501)
Supplement: Supplementary file 1 [file molecules-29-00501-s001.zip › molecules-2810949-supplementary.pdf]

Supporting information

Reactivity and stability of the (hetero)benzylic alkenes via Wittig olefination reaction

Ajmir Khan *et al*

Corresponding authors:
Dr. Ajmir Khan*
Email: khanajmi@msu.edu Tel:
+1-517-483-3827

This **PDF** includes

1) Some common protocoals and data characterization
2) Spectra (^{1}H and ^{13}C NMR)
3) References

1. **6,7-Dihydrobenzofuran-4(5*H*)-one (4b).**[1]

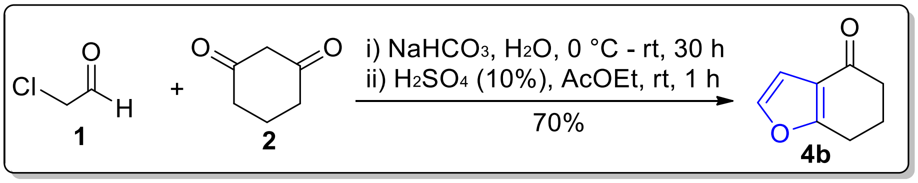

A round bottom flask was charged with a solution of **2** (1.68 g, 15.0 mmol) in AcOEt (20 mL), $NaHCO_3$ (1.26 g, 15.0 mmol) and H_2O (22 mL). To the above mixture, 50% aqueous solution of **1** (3.5 g, 45.0 mmol) was added at 0 °C. The mixture was stirred for 30 hours at rt. To the reaction mixture conc; H_2SO_4 (1.68 g) was added and stirred for additional 1 hour at rt. AcOEt (15 mL) was then added followed by the organic layer separation and washed with aqueous $NaHCO_3$ (20 mL), brine (20 mL), dried over anhydrous $MgSO_4$ and concentrated under reduced pressure.
Purification: The residue was purified by flash column chromatography (20% EtOAc in hexanes).
Yield: `70% (1.43 g, 10.5 mmol).
Sample appearance: Light yellow oil.
^{1}H NMR (300 MHz, $CDCl_3$) δ: 2.18 (2H, q, J = 6.4 Hz), 2.50 (2H, t, J = 6.4 Hz), 2.88 (2H, t, J = 6.3Hz), 6.67 (d, J = 1.9 Hz, 1H), 7.32 (d, J = 1.9 Hz, 1H).
^{13}C NMR (75 MHz, $CDCl_3$) δ: 22.4, 23.1, 37.4, 106.2, 120.8, 142.4, 167.0, 194.4.

2. **6,6-Dimethyl-6,7-dihydrobenzofuran-4(5*H*)-one (4c).**[1]

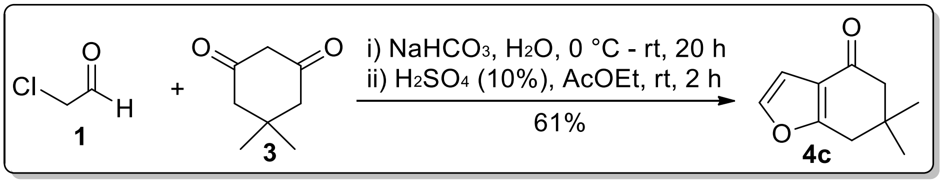

The mixture of **1** (1.39 g, 17.8 mmol, 3 ml of a 50% weight solution in water) and $NaHCO_3$ (2.1 g, 25.0 mmol) in H_2O (17.5 ml) were stirred together and cooled to 5 °C. To the above solution, dimedone **3** (2.45 g, 17.5 mmol) dissolved in H_2O (17.5 ml) was added dropwise and stirred for 20 hours at rt. Reaction mixture was diluted with EtOAc (22.5 ml) and aqueous H_2SO_4 (10%) was added to achieve the pH 2. The mixture was stirred for further 2 h at room temperature. Neutralization to pH 7 was achieved by portion wise addition of solid $NaHCO_3$. The reaction mixture was then extracted with EtOAc (3 × 10 ml) and dried over anhydrous $MgSO_4$, concentrated in vacuum.
Purification: The residue was purified by flash column chromatography (20% EtOAc in hexanes)
Yield: 61% (1.76 g, 10.7 mmol).
Sample appearance: Light yellow oil.
^{1}H NMR (300 MHz, $CDCl_3$) δ: 1.14 (6H, s), 2.37 (2H, s), 2.75 (2H, s), 6.65 (1H, d, *J* = 1.6 Hz), 7.34 (1H, d, *J* = 1.6 Hz).
^{13}C NMR (75 MHz, $CDCl_3$) δ: 28.4, 35.2, 37.2, 51.9, 106.2, 119.8, 142.9, 166.2, 193.8.

3. **3,4,6,7,8,9-Hexahydrodibenzo[b,d]furan-1(2*H*)-one (4s).**[2]

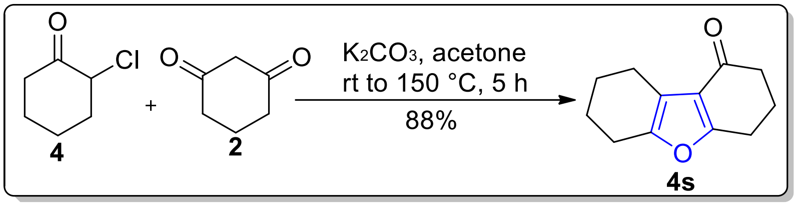

To the stirred solution of **2** (1.0 g, 8.92 mmol) and K_2CO_3 (1.34 g, 9.78 mmol) in 100 ml of acetone, **4** (1.18 g, 8.92 mmol) were added dropwise to the flask at 0 °C. The resulting mixture was allowed to heat 0 °C to 150 °C and stirred for 5 hours. Next, 20 ml of H_2O was added into the reaction flask, agitation was carried out, and HCl (6 N) was slowly added into the reaction flask to acidify the solution until pH was reached to 6-7. The residue of the reaction mixture was extracted with DCM (3 × 20 ml), and the combined organic layers were dried over anhydrous $MgSO_4$. The solvent was removed under reduced pressure.
Purification: The residue was purified by flash column chromatography (10% EtOAc in hexanes).
Yield: 88% (1.44 g, 7.84 mmol).
Sample appearance: Light yellow oil.
^{1}HNMR (300 MHz, $CDCl_3$) δ: 1.72-1.85 (4H, m), 2.13 (2H, q, *J* = 6.2 Hz), 2.44 (2H, t, *J* = 6.4 Hz), 2.55 (2H, t), 2.63 (2H, t, *J* = 5.8 Hz), 2.81 (2H, t, *J* = 6.2Hz).
^{13}C NMR (75 MHz, $CDCl_3$) δ: 21.5, 22.6, 22.6, 22.8, 22.8, 23.5, 38.1, 115.3, 120.3, 150.8, 165.5, 195.5.

4. **2,3-dihydro-1H-carbazol-4(9H)-one (3d).**[3,4]

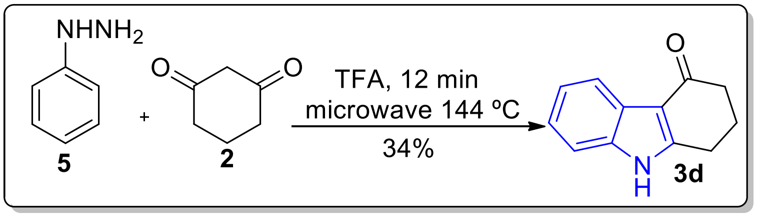

To a microwave vial, **2** (1.40 g, 10.0 mmol) and **5** (1.3 g, 12.0 mmol) were added in TFA (4.0 mL). The reaction mixture was carried out in a microwave reactor at 144 °C for 10-12 min. After the

completion of reaction, TFA was evaporated under vacuumed pressure. The crude reaction mixture was saturated with $NaHCO_3$ aqueous solution (20 mL). The aqueous layer was extracted with EtOAc (3 × 20 mL), dried over anhydrous $MgSO_4$ and filtered. The solvents were evaporated under reduced pressure.
Purification: The residue was purified by flash column chromatography (50% EtOAc in hexanes).
Yield: 34% (0.441 g, 2.38 mmol).
Sample appearance: Light brown solid.
Milting Point: 225-227 ºC (lit 223 °C).[5]
^{1}H NMR (300 MHz, MeOD) δ: 2.10 (2H, q, *J* = 6.2 Hz), 2.43 (2H, t,*J* = 6.4 Hz), 2.95 (2H, t, *J* = 6.1 Hz), 7.14-7.18 (2H, m), 7.41 (1H, d, *J* = 6.6 Hz), 7.98 (1H, d, *J* = 7.8 Hz), 11.86 (1H, s).
^{13}C NMR (75 MHz, MeOD) δ: 24.3, 25.2, 39.0, 112.6, 113.7, 122.1, 123.3, 124.3, 126.3, 138.0, 155.1, 197.3.

5. **2,2-dimethyl-2,3-dihydro-1H-carbazol-4(9H)-one (3e).**[6]

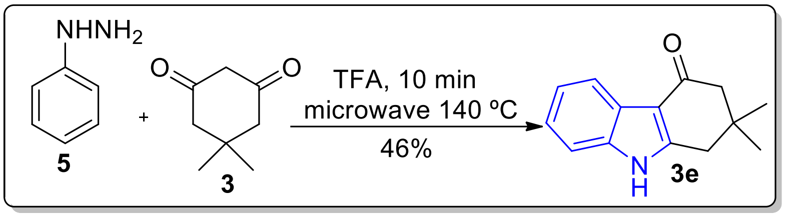

The reaction was performed following the general protocol, using **3** (0.784 g, 7.0 mmol), **5** (0.864 g, 8.0 mmol) and TFA (4.0 mL). The reaction was carried out in a microwave reactor at 140°C for 10-12 minutes.
Purification: The residue was purified by flash column chromatography (50% EtOAc in hexanes).
Yield: 46% (0.98 g, 4.6 mmol).
Sample appearance: Brown solid.
Milting Point: 207 ºC (lit 209-210 °C).[5]
^{1}H NMR (300 MHz, $CDCl_3$) δ: 1.16 (6H, s), 2.46 (2H, s), 2.83 (2H, s), 7.21-7.27 (2H, m), 7.35 (1H, d, *J* = 8.7 Hz), 8.20 (1H, d, *J* = 8.7 Hz), 8.91 (1H, s).
^{13}C NMR (75 MHz, $CDCl_3$) δ: 28.8, 35.9, 37.5, 52.5, 111.1, 112.3, 121.5, 122.6, 123.3, 124.8, 136.1, 150.4, 193.9.

6. **2,3,4,9-Tetrahydro-*1H*-carbazol-1-one (3f).**[3]

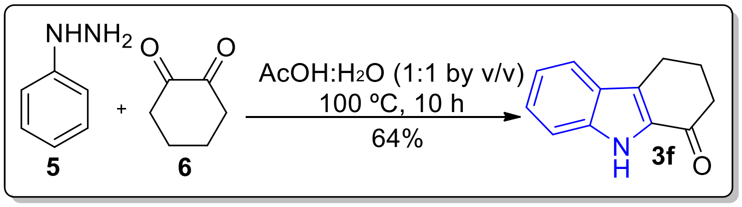

6 (1.5 g, 13.4 mmol) and **5** (1.6 g, 14.8 mmol) were dissolved in a mixture of acetic acid (20 mL) and water (20 mL) 1:1 (v/v) than refluxed (90-100 ºC) for 10 h. The reaction mixture was quenched with aqueous $NaHCO_3$ (1M, 0.5 L) with cautions. The precipitate was then filtered and washed with water and solvents were evaporated under reduced pressure.
Purification: The residue was purified by flash column chromatography (20% EtOAc in hexanes)
Yield: 64% (1.57 g, 8.5 mmol).
Sample appearance: Light brown solid.
Milting Point: 166-168 °C (lit 170 °C).[7]
^{1}H NMR (300 MHz, $CDCl_3$) δ: 2.27 (2H, q, *J* = 6.2 Hz), 2.68 (2H, t, *J* = 6.4 Hz), 3.01 (2H, t, *J* = 6 Hz), 7.14 (1H, t, *J* = 7 Hz) 7.36 (1H, t, *J* = 7 Hz), 7.45 (1H, d, *J* =5.4 Hz), 7.65 (1H, d, *J* = 5.1 Hz), 9.45 (1H, s).
^{13}C NMR (75 MHz, $CDCl_3$) δ: 21.5, 25.1, 38.3, 112.7, 120.4, 121.4, 125.9, 127.1, 129.8, 131.3, 138.1, 191.7.

7. **9-Methyl-1,2,3,5,6,7,8,9-octahydro-4*H*-carbazol-4-one 2 (4t).**[8]

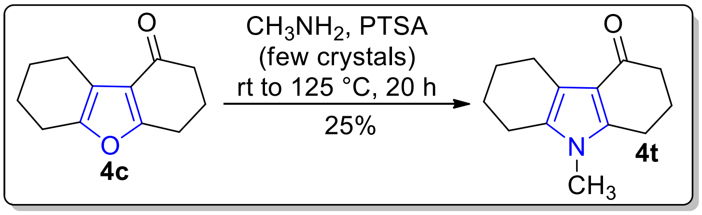

The reaction was performed following the general protocol, using **4c** (1.14 g, 6.0 mmol), CH_3NH_2 (3 ml) and *p*-TsOH (few crystals).
Purification: The residue was purified by flash column chromatography (30-40% EtOAc in hexanes).
Yield: 25% (0.302 g, 1.49 mmol).
Sample appearance: Brown solid.
Milting Point : 122 - 124 °C (lit 120 °C).[8]
^{1}HNMR (300 MHz, $CDCl_3$) δ: 1.66-1.86 (4H, m), 2.11 (2H, q, *J* = 6.3 Hz), 2.41 (2H, t, *J* = 6.4 Hz), 2.48 (2H, t, *J* = 6 Hz), 2.68-2.77 (4H, m), 3.38 (3H, s).
^{13}C NMR (75 MHz, $CDCl_3$) δ: 21.5, 21.9, 22.7, 23.0, 23.2, 23.7, 29.9, 38.3, 116.6, 117.5, 129.3, 142.7, 194.6.

8. **1-Methyl-1,5,6,7-tetrahydro-4H-indol-4-one (4u).**[9]

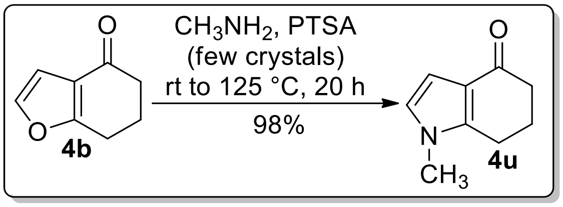

The reaction was performed following the general protocol, using **4b** (0.408 g, 3.0 mmol) and CH_3NH_2 (5 ml).
Purification: The residue was purified by flash column chromatography (40% EtOAc in hexanes).
Yield: 98% (0.44 g, 2.95 mmol).
Sample appearance: Brown crystal.
Milting Point: 88 °C (lit 84 - 85 °C).[10]
^{1}HNMR (300 MHz, $CDCl_3$) δ: 2.15 (2H, q, *J* = 6.3 Hz), 2.44 (2H, t, *J* = 6 Hz), 2.73 (2H, t, *J* = 6.3 Hz), 6.51 (1H, d, *J* = 3 Hz), 6.54 (1H, d, *J* = 3.0 Hz).
^{13}C NMR (75 MHz, $CDCl_3$) δ: 21.5, 23.6, 33.5, 37.6, 105.2, 120.7, 123.2, 143.8, 194.2.

9. **1-(3-Chlorophenyl)-1,5,6,7-tetrahydro-4*H*-indol-4-one (4v).**[11]

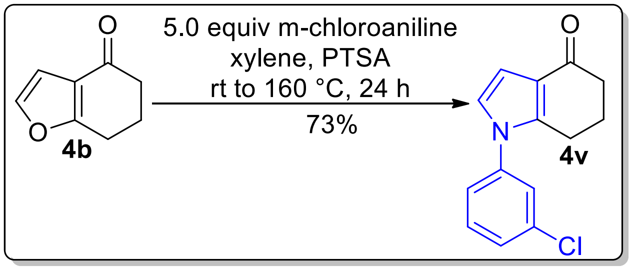

The reaction was performed following the general protocol, using **4b** (0.408 g, 3.0 mmol), *m*-chloroaniline (1.9 g, 15.0 mmol), xylene (5 mL), and few crystals of *p*-TsOH.
Purification: The residue was purified by flash column chromatography (40% EtOAc in hexanes).
Yield: 73% (0.538 g, 2.19 mmol).
Sample appearance: Brown crystal.
Milting Point: 118 - 120 °C (lit;121 °C).[11]

^{1}HNMR (300 MHz, $CDCl_3$) δ: 2.14 (2H, q, J = 6.3 Hz), 2.52 (2H, t, J = 6.4 Hz), 2.79 (2H, t, J = 6.1 Hz), 6.69 (1H, d, J = 3.0 Hz), 6.79 (1H, d, J = 3.0 Hz), 7.22 (1H, d, J = 7.5 Hz), 7.33-7.46 (3H, m).
^{13}C NMR (75 MHz, $CDCl_3$) δ: 23.1, 24.0, 37.8, 107.0, 122.2, 123.0, 123.1, 125.2, 128.0, 130.6, 135.2, 140.0, 143.3, 194.7.

10. **1,5,5-Trimethyl-1,5,6,7-tetrahydro-4*H*-indol-4-one (4w).**

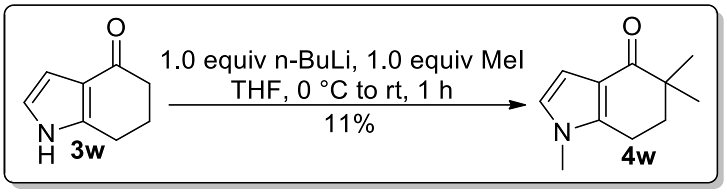

To a solution of **3w** (0.405 mg, 3.0 mmol) in THF (4 mL) at 0 °C was added *n*-BuLi (0.192 g, 3.0 mmol, 1.62 ml) and stirred for 30 min. MeI (0.426 g, 3.0 mmol) was added dropwise to the reaction mixture and allowed to rt, stirred for 1 h. After completion of the reaction, it was quenched with H_2O (5 mL). The reaction mixture was extracted with EtOAc (3 × 10 ml), washed with brine, and dried over $MgSO_4$. The solvents were evaporated under reduced pressure.
Purification: The residue was purified by silica gel column chromatography (25% EtOAc in hexanes).
Yield: 11% (0.06 g, 0.333 mmol).
Sample appearance: Colorless oil.
^{1}HNMR (300 MHz, $CDCl_3$) δ: 1.16 (6H, s), 1.98 (2H, t, J = 6.1 Hz), 2.73 (2H, t, J = 6.1 Hz), 3.53 (3H, s), 6.49 (1H, d, J = 3.0 Hz), 6.53 (1H, d, J = 3.0 Hz).
^{13}C NMR (75 MHz, $CDCl_3$) δ: 18.9, 24.5, 33.3, 37.5, 41.3, 106.0, 119.1, 123.4, 142.0, 199.0.
HRMS [ESI(+)] calcd. for $[C_{11}H_{15}NO+H]^+$, 178.1226, found 178.1230.
IR (film): 3280, 2950, 2928, 2887, 2857, 1654, 1599, 1376, 1287, 1263, 1114, 844, 822.

11. **6,7-Dihydroisoquinolin-8(5*H*)-one (4k)** and
7,8-dihydroisoquinolin-5(6*H*)-one (4j).[12]

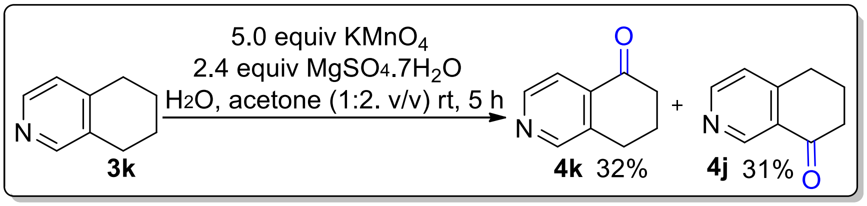

Purification: The residue was purified by flash column chromatography (30-50% EtOAc in hexanes).
6,7-Dihydroisoquinolin-8(5*H*)-one (**4k**).
Yield: 31% (0.455 g, 3.1 mmol).
Sample appearance: Light yellow oil.
^{1}HNMR (500 MHz, $CDCl_3$) δ: 2.12-2.18 (2H, q, J = 6.4), 2.69 (2H, t, J = 6.5), 2.96 (2H, t, J = 6.0), 7.17 (1H, d, J = 5.5), 8.58 (1H, d, J = 5.0), 9.12 (1H, s).
^{13}C NMR (125 MHz, $CDCl_3$) δ: 22.5, 29.0, 39.2, 123.6, 128.0, 149.3, 152.6, 153.0, 197.5.
7,8-Dihydroisoquinolin-5(6*H*)-one (**4j**).
Yield: 32% (0.47 g, 3.2 mmol).
Sample appearance: Light yellow oil.
^{1}HNMR (500 MHz, $CDCl_3$) δ: 2.15-2.20 (2H, q, J = 6.4), 2.69 (2H, t, J = 6.5), 2.94 (2H, t, J = 6.0), 7.74 (1H, d, J = 5.0), 8.60 (1H, d, J = 5.0), 8.64 (1H, s).
^{13}C NMR (125 MHz, $CDCl_3$) δ: 23.0, 26.4, 39.2, 119.2, 137.6, 137.7, 148.5, 151.4, 197.7.

12. **9-Acetyl-1,2,3,9-tetrahydro-4*H*-carbazol-4-one (4d).**[6]

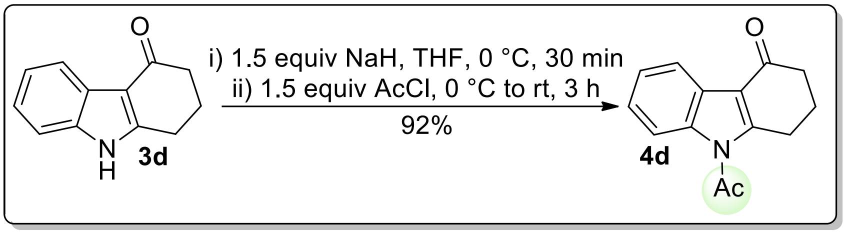

The reaction was performed following the general protocol, using **3d** (0.555 g, 3.0 mmol), NaH (0.18 g, 4.5 mmol) and AcCl (0.353 g 4.5 mmol) in THF (10 mL).
Purification: The residue was purified by flash column chromatography (33% EtOAc in hexanes).
Yield: 92% (0.625 g, 2.76 mmol).
Sample appearance: Light brown solid.
Milting Point: 201-204 °C.
^{1}H NMR (300 MHz, $CDCl_3$) δ: 2.21 (2H, m, *J* = 6.0 Hz), 2.56 (2H, t, *J* = 6.4 Hz), 2.76 (3H, s), 3.24 (2H, t, *J* = 5.7 Hz), 7.31-7.34 (2H, m), 7.83 (1H, d, *J* = 5.1 Hz), 8.35 (1H, d, *J* = 4.8 Hz).
^{13}C NMR (75 MHz, $CDCl_3$) δ: 23.6, 26.4, 27.6, 37.8, 114.4, 117.6, 122.0, 124.7, 125.1, 126.2, 135.5, 151.7, 170.4, 195.7.

13. **9-Tosyl-1,2,3,9-tetrahydro-4*H*-carbazol-4-one (4e)**[13]

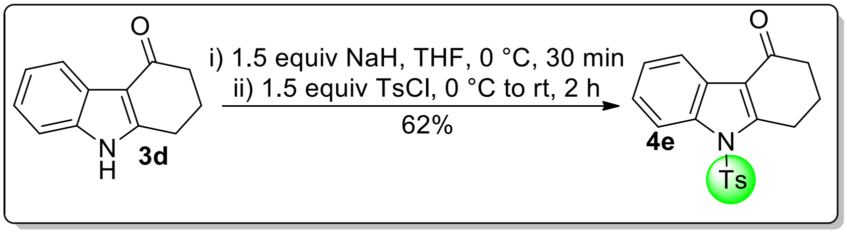

The reaction was performed following the general protocol, using **3d** (0.185 g, 1.0 mmol), NaH (0.06 g, 1.5 mmol, 60% dispersion in mineral oil) and TsCl (0.285 g, 1.5 mmol) in anhydrous THF (8 mL).
Purification: The residue was purified by flash column chromatography (25% EtOAc in hexanes).
Yield: 62% (0.211 g, 0.622 mmol).
Sample appearance: White solid.
Milting Point: 154-156 °C (lit 152-153 °C).[13]
^{1}H NMR (300 MHz, $CDCl_3$) δ: 2.16-2.25 (2H, q, *J* = 6.3 Hz), 2.35 (3H, s), 2.55 (2H, t, *J* = 6.6 Hz), 3.32 (2H, t, *J* = 6.1 Hz), 7.25 (2H, d, *J* = 8.7 Hz), 7.31-7.35 (2H, m), 7.75 (2H, d, *J* = 8.4 Hz), 8.15 (1H, d, *J* = 8.7 Hz), 8.24 (1H, d, *J* = 9 Hz).
^{13}C NMR (75 MHz, $CDCl_3$) δ: 21.7, 23.3, 24.6, 37.9, 113.9, 118.0, 122.0, 125.0, 125.4, 125.8, 126.7, 130.3, 135.6, 136.0, 145.9, 151.0, 195.1.

14. **9-Acetyl-2,2-dimethyl-1,2,3,9-tetrahydro-4*H*-carbazol-4-one (4f).**[6]

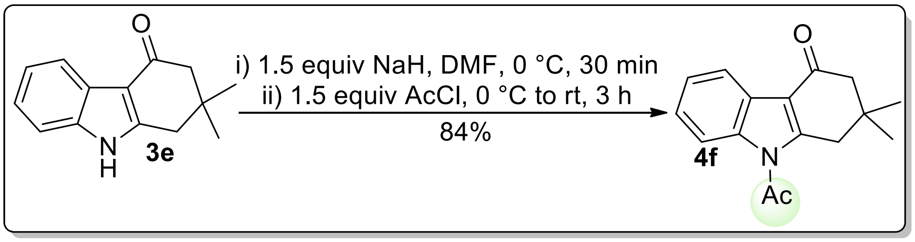

The reaction was performed following the general protocol, using **3e** (0.765 g, 3.0 mmol), NaH (0.18 g, 4.5 mmol) and AcCl (0.353 g 4.5 mmol) in DMF (10 mL).
Purification: The residue was purified by flash column chromatography (50% EtOAc in hexanes).

Yield: 84% (0.642 g, 2.52 mmol).
Sample appearance: Brown solid.
Milting Point: 181 -183 °C. (lit 184 °C).[6]
^{1}H NMR (300 MHz, $CDCl_3$) δ: 1.16 (6H, s), 2.45 (2H, s), 2.79 (3H, s), 3.14 (2H, s), 7.30-7.37 (2H, m), 7.82 (1H, d, *J* = 9.3 Hz), 8.35 (1H, d, *J* = 9 Hz).
^{13}C NMR (75 MHz, $CDCl_3$) δ: 27.7, 28.7, 35.2, 40.3, 51.7, 114.4, 116.7, 122.0, 124.7, 125.0, 126.1, 135.8, 150.5, 170.5, 195.3.

15. **2,2-Dimethyl-9-tosyl-1,2,3,9-tetrahydro-*4H*-carbazol-4-one (4g).**

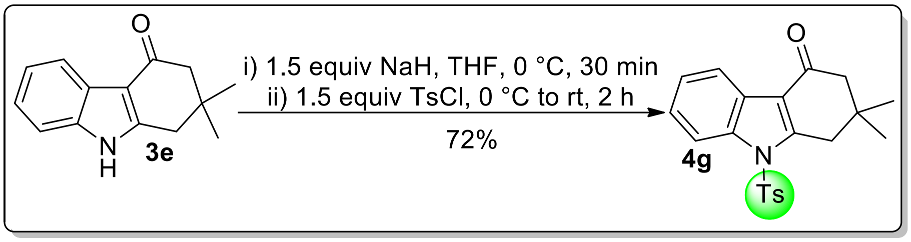

The reaction was performed following the general protocol but using **3e** (0.639 g, 3.0 mmol), Boc (0.85 g, 4.5 mmol), THF (15 mL).
Purification: The residue was purified by flash column chromatography (50% EtOAc in hexanes).
Yield: 72% (0.792 g, 2.16 mmol).
Sample appearance: Light brown solid.
Milting Point: 171-172 °C.
^{1}H NMR (300 MHz, $CDCl_3$) δ: 1.13 (6H, s), 2.37 (3H, s), 2.4 (2H, s), 3.22 (2H, s), 7.26 (2H, d, *J* = 8.1 Hz), 7.34 (2H, t, *J* = 3.9 Hz), 7.74 (2H, d, *J* = 8.4 Hz), 8.14-8.17 (2H, m), 8.22 (2H, t, *J* = 9 Hz).
^{13}C NMR (75 MHz, $CDCl_3$) δ: 21.8, 28.7, 35.3, 38.5, 52.0, 114.1, 117.1, 121.9, 125.1, 125.4, 125.7, 126.7, 130.3, 135.7, 136.4, 146.0, 150.0 194.9.
HRMS [ESI(+)] calcd. for $[C_{21}H_{21}NO_3S+H]^+$ 368.1320, found 368.1311.
IR (film): 3339, 3059, 2959, 1669, 1597, 1559, 1450, 1408, 1176, 917, 813, 751, 665 cm^{-1}.

16. ***t*-Butyl 1-oxo-1,2,3,4-tetrahydro-9*H*-carbazole-9-carboxylate (4h).**

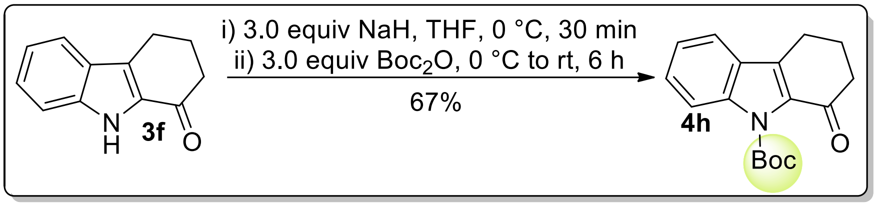

The reaction was performed following the general protocol, using **3f** (0.278 g, 1.5 mmol), NaH (0.18 g, 4.5 mmol) and Boc_2O (0.981 g, 4.5 mmol) in anhydrous THF (10 mL).
Purification: The residue was purified by flash column chromatography (10% EtOAc in hexanes).
Yield: 67% (0.288 g, 1.01 mmol).
Sample appearance: white solid.
Milting Point: 150-152 °C.
^{1}H NMR (300 MHz, $CDCl_3$) δ: 1.63 (6H, s), 2.23 (2H, q, *J* = 6.3 Hz), 2.66 (2H, t, *J* = 6.4 Hz), 2.93 (2H, t, *J* =6 Hz), 7.25 (1H, t, *J* = 5 Hz), 7.46 (1H, t, *J* = 5.2 Hz), 7.58 (1H, d, *J* =8.1 Hz), 8.05 (1H, d, *J* = 8.4 Hz).
^{13}C NMR (75 MHz, $CDCl_3$) δ: 21.8, 24.0, 27.8, 39.4, 84.1, 114.9, 121.1, 123.0, 126.8, 128.8, 132.4, 137.0, 139.1, 149.8, 188.0.
HRMS [ESI(+)] calcd. for $[C_{17}H_{19}NO_3+H]^+$ 286.1443, found286.1444.
IR (film): 3402, 3074, 2978, 2935, 2869, 2324, 1756, 1710, 1609, 1582, 1465, 1432, 1395, 1370, 1351, 1327, 1260, 1234, 1146, 1075, 1060, 1034, 950, 916, 880, 798, 783, 747, 706 cm^{-1}.

17. **9-(Methylsulfonyl)-2,3,4,9-tetrahydro-1*H*-carbazol-1-one (4i).**

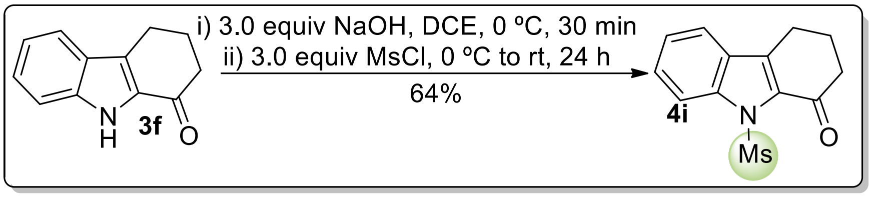

The reaction was performed following the general protocol**,** using **3f** (0.536 g, 2.89 mmol), NaOH (0.44 g, 11.0 mmol) and MsCl (0.988 g, 8.67 mmol) 1,2-dichloroethane (20 mL).
Purification: The residue was purified by flash column chromatography (25% EtOAc in hexanes).
Yield: 64% (0.486 g, 1.84 mmol).
Sample appearance: Dark brown solid.
Milting Point: 188-191 °C.
^{1}H NMR (300 MHz, $CDCl_3$) δ: 2.24 (2H, q, *J* = 6.3 Hz), 2.70 (2H, t, *J* = 6.4 Hz), 2.97 (2H, t, *J* = 6.1 Hz), 3.79 (3H, s), 7.30 (1H, t, *J* = 7.5 Hz), 7.48 (1H, t, *J* = 7.9 Hz), 7.62 (1H, d, *J* = 7.2 Hz), 8.12 (1H, d, *J* = 8.7 Hz).
^{13}C NMR (75 MHz, $CDCl_3$) δ: 21.8, 23.6, 39.3, 43.8, 115.8, 121.4, 123.6, 126.9, 129.2, 132.3, 138.3, 139.3, 189.0.
HRMS [ESI(+)] calcd. for $[C_{13}H_{13}NO_3S+Na]^+$ 286.0514, found 286.0505.
IR (film): 3468, 3119, 3012, 2928, 1672, 1555, 1496, 1444, 1414, 1369, 1335, 1287, 1177, 1145, 1125, 1111, 1054, 1009, 973, 897, 854, 774, 749, 711, 666 cm^{-1}.

18. **1-(Methylsulfonyl)-1,5,6,7-tetrahydro-4*H*-indol-4-one (4o).**

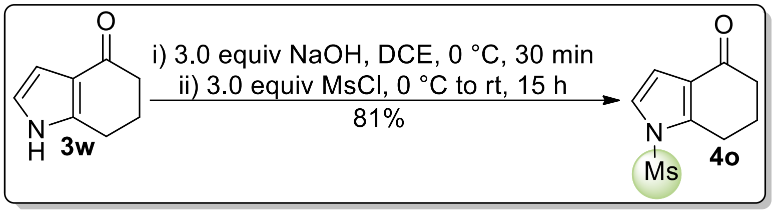

The reaction was performed following the general protocol**,** using **3w** (0.50 g, 3.7 mmol), NaOH (0.44 g, 11.0 mmol) and MsCl (1.27 g. 11.1 mmol) in DCE (15 mL).
Purification: The residue was purified by flash column chromatography (50% EtOAc in hexanes)
Yield: 81% (0.638 g, 2.99 mmol).
Sample appearance: Dark brown solid.
Milting Point: 171-172 °C.
^{1}H NMR (300 MHz, $CDCl_3$) δ: 2.17-2.26 (2H, q, *J* = 6.4 Hz,), 2.51 (2H, t, *J* = 6.4 Hz), 3.09 (2H, t, *J* = 6.1 Hz), 3.24 (3H, s), 6.65 (1H, d, *J* = 3.3 Hz), 7.10 (1H, d, *J* = 3.3 Hz).
^{13}C NMR (75 MHz, $CDCl_3$) δ: 22.2, 23.5, 37.5, 43.1, 108.5, 122.0, 125.0, 143.7, 194.4.
HRMS [ESI(+)] calcd. for $[C_9H_{11}NO_3S+H]^+$ 214.0538, found 214.0531.
IR (film): 3468, 3119, 2928, 1672, 1444, 1369, 1125, 1111, 774.

19. ***t*-Butyl 4-oxo-4,5,6,7-tetrahydro-1*H*-indole-1-carboxylate (4p).**[5]

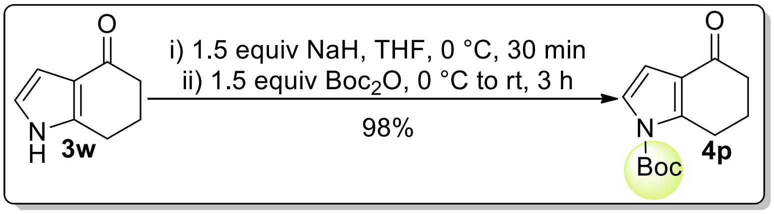

The reaction was performed following the general protocol, using **3w** (0.27 g, 2.0 mmol), NaH (0.12 g, 3.0 mmol, 60% dispersion in mineral oil) and Boc_2O (0.657 g, 3.0 mmol) in anhydrous THF (6 mL).
Purification: The residue was purified by flash column chromatography (33% EtOAc in hexanes)
Yield: 98% (0.465 g, 1.97 mmol).
Sample appearance: White colorless solid.
Milting Point: 78-80 ℃ (lit 79-80 ℃).
^{1}H NMR (300 MHz, $CDCl_3$) δ: 1.60 (9H, s), 2.09-2.18 (2H, q, J = 6.4 Hz), 2.46 (2H, t, J = 6.4 Hz), 3.12 (2H, t, J = 6.2 Hz) 6.53 (1H, d, J = 3.6 Hz), 7.15 (1H, d, J = 3.6 Hz).
^{13}C NMR (75 MHz, $CDCl_3$) δ: 21.7, 23.6, 37.6, 50.6, 105.6, 121.2, 123.0, 126.6, 128.0, 129.0, 136.5, 143.6, 194.2.

20. **1-(*t*-Butyldimethylsilyl)-1,5,6,7-tetrahydro-4*H*-indol-4-one (4q).**

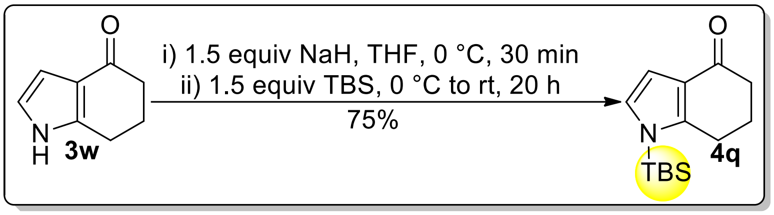

The reaction was performed following the general protocol, using **3w** (0.135 g, 1.0 mmol), NaH (0.06 g 1.5 mmol, 60% dispersed in mineral oil) and TBDMS-Cl (0.225 g, 1.5 mmol) in anhydrous THF (5 mL).
Purification: The residue was purified by flash column chromatography (20% EtOAc in hexanes).
Yield: 75% (0.186 g, 0.75 mmol.
Sample appearance: Light yellow oil.
^{1}H NMR (300 MHz, $CDCl_3$) δ: 0.41 (6H, s), 0.82, (9H, s), 2.02 (2H, q, J = 6.1 Hz), 2.36 (2H, t, J = 6.3 Hz), 2.74 (2H, t, J = 6.1 Hz), 6.51 (1H, d, J = 2.5) 6.56 (1H, d, J = 2.5 Hz).
^{13}C NMR (75 MHz, $CDCl_3$) δ: 3.7, 18.9, 24.3, 25.4, 26.0, 37.7, 107.3, 124.0, 125.6, 149.2, 195.0.
HRMS [ESI(+)] calcd. for $[C_{14}H_{23}NOSi+H]^+$ 250.1627, found 250.1623.
IR (film): 3436, 2959, 2925, 1652, 1512, 1471, 1425, 1233, 940, 721.

21. **1-Benzyl-1,5,6,7-tetrahydro-4*H*-indol-4-one (4r).**[14]

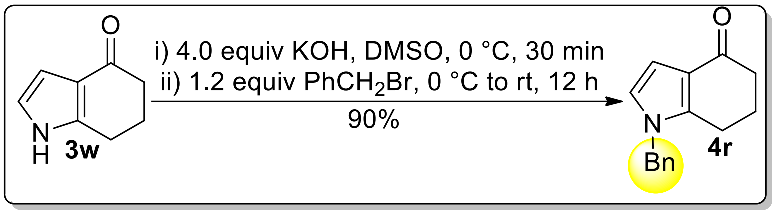

The reaction was performed following the general protocol, using **3w** (0.27 g, 2.0 mmol), KOH (0.448 g, 8.0 mmol) and BnBr (0.41 g, 2.4 mmol) in DMSO (6 mL).
Purification: The residue was purified by flash column chromatography (33% EtOAc in hexanes).
Yield: 90% (0.405 g, 1.8 mmol).
Sample appearance: Light red oil.
^{1}H NMR (300 MHz, $CDCl_3$)δ: 2.09 (2H, q, J = 6.4 Hz), 2.43 (2H, t, J = 6.45 Hz), 2.64 (2H, t, J = 6.15 Hz), 5.03 (2H, s) 6.59 (2H, dd, J = 8.7 and 3.0 Hz), 7.04, (2H, d, J = 6.6 Hz), 7.25-7.36 (3H, m).
^{13}C NMR (75 MHz, $CDCl_3$) δ: 21.7, 23.6, 37.6, 50.6, 105.6, 121.2, 123.0, 126.6, 128.0, 129.0, 136.5, 143.6, 194.2.

22. **4,6,7,8-Tetrahydro-*5H*-cyclohepta[*b*]thiophen-5-one** **(4aa)**[15]

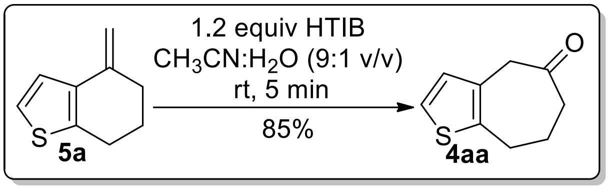

To a solution of **5a** (0.15 g, 1.0 mmol) in CH_3CN 90% (5 mL of CH_3CN:H_2O, 9:1, v/v) was added HTIB (0.47 g, 1.2 mmol, 1.2 equiv) and stirred for 5 min at rt. The reaction was monitored by TLC for the consumption of the starting material. The reaction mixture was quenched with saturated solution of $NaHCO_3$, extracted with DCM (3 x 10 mL). The combined organic extracts were washed with brine (10 mL), dried over $MgSO_4$ and filtered. The solvent was removed under reduced pressure.
Purification: The residue was purified by flash column chromatography (5% EtOAc in hexanes).
Yield: 85% (0.141 g, 0.85 mmol).
Sample appearance: Yellowish oil.
^{1}H NMR (300 MHz, $CDCl_3$) δ: 2.11-2.19 (2H, q, *J* = 6.1 Hz), 2.66 (2H, t, *J* = 6.3 Hz), 3.04 (2H, t, *J* = 6.0 Hz), 3.75 (2H, s), 6.71 (1H, d, *J* = 4.8 Hz), 7.02 (1H, d, *J* = 5.1 Hz).
^{13}C NMR (75 MHz, $CDCl_3$) δ: 24.4, 28.4, 43.9, 44.4, 122.4, 128.6, 129.9, 137.8, 208.1.

2. ^{1}H NMR and ^{13}C NMR Spectra

of the (all syntheszed compounds in thos study)

6,7-Dihydrobenzofuran-4(5*H*)-one (4b).

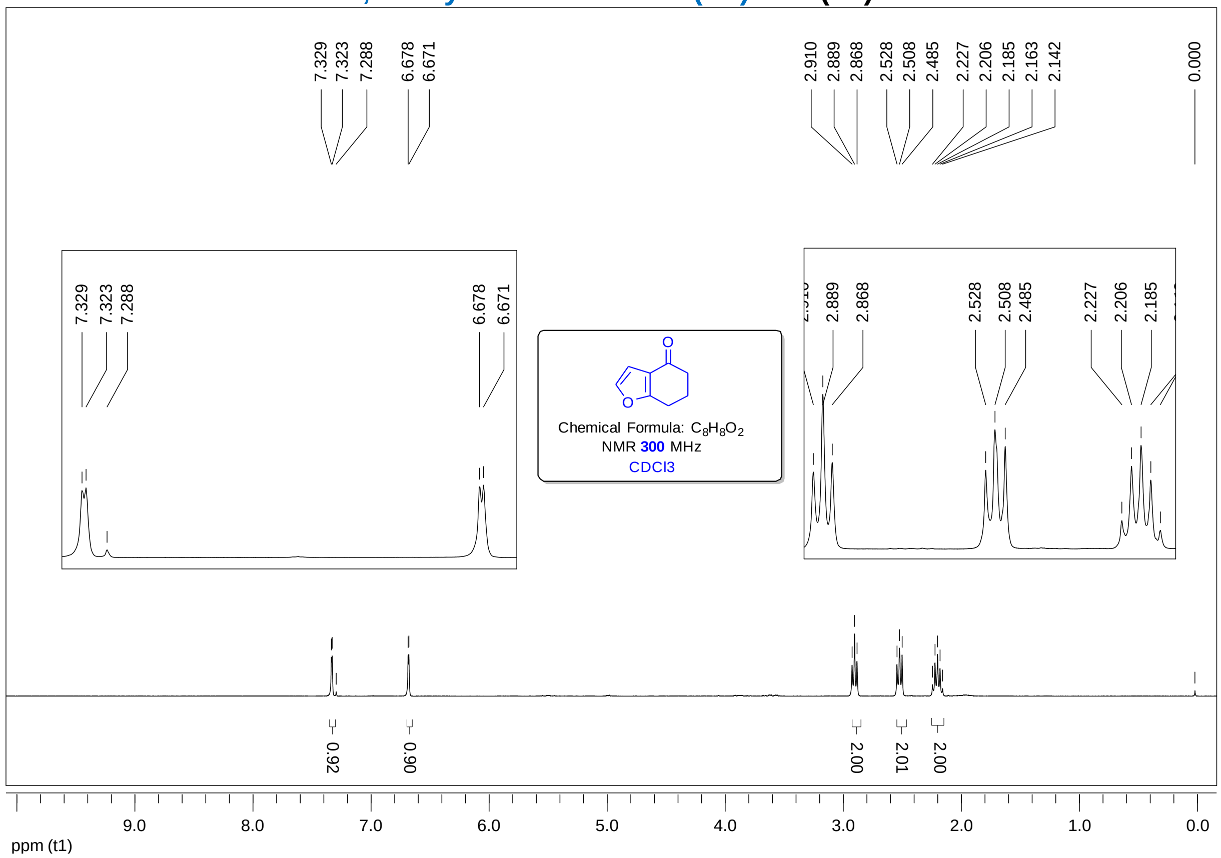

6,7-Dihydrobenzofuran-4(5*H*)-one (4b).

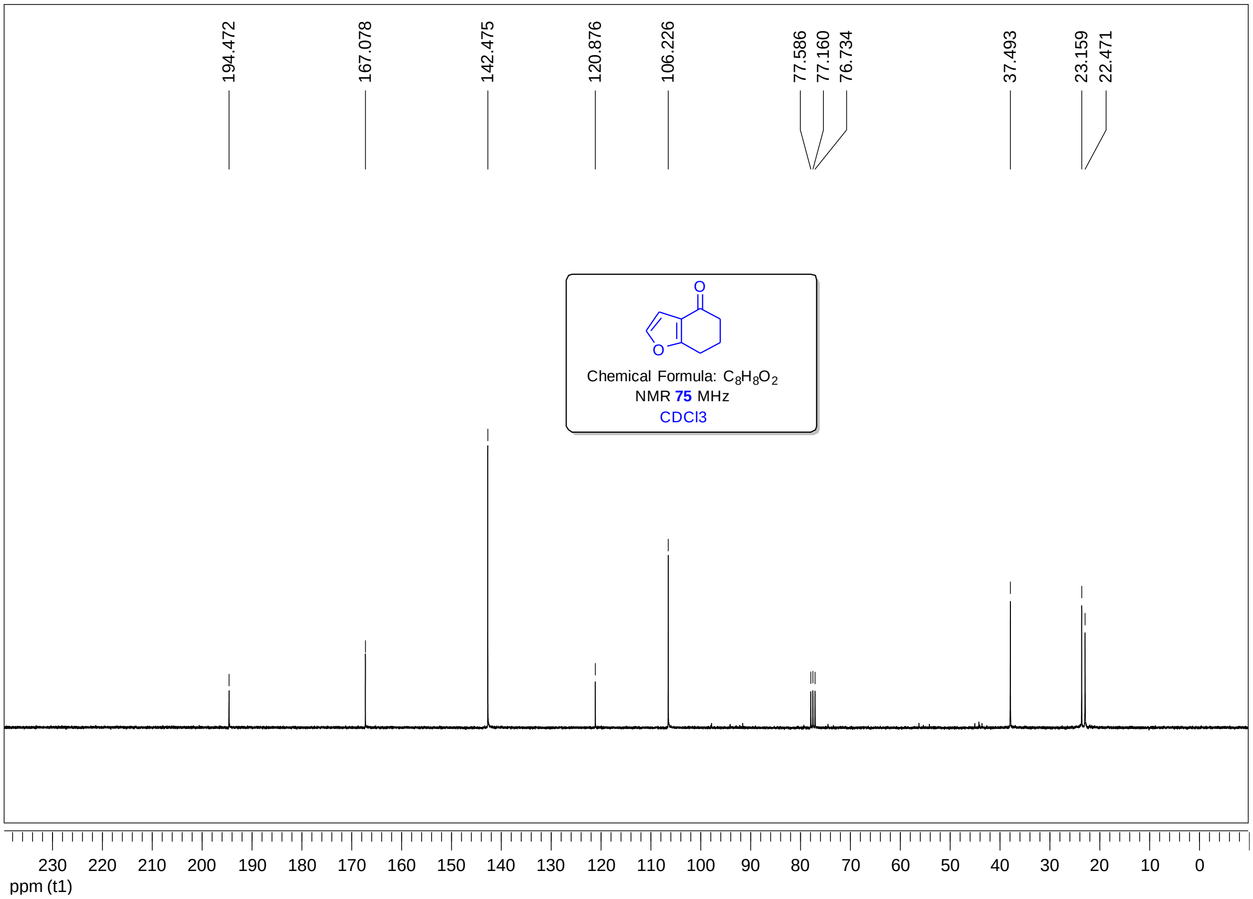

6,6-Dimethyl-6,7-dihydrobenzofuran-4(5*H*)-one (4c)

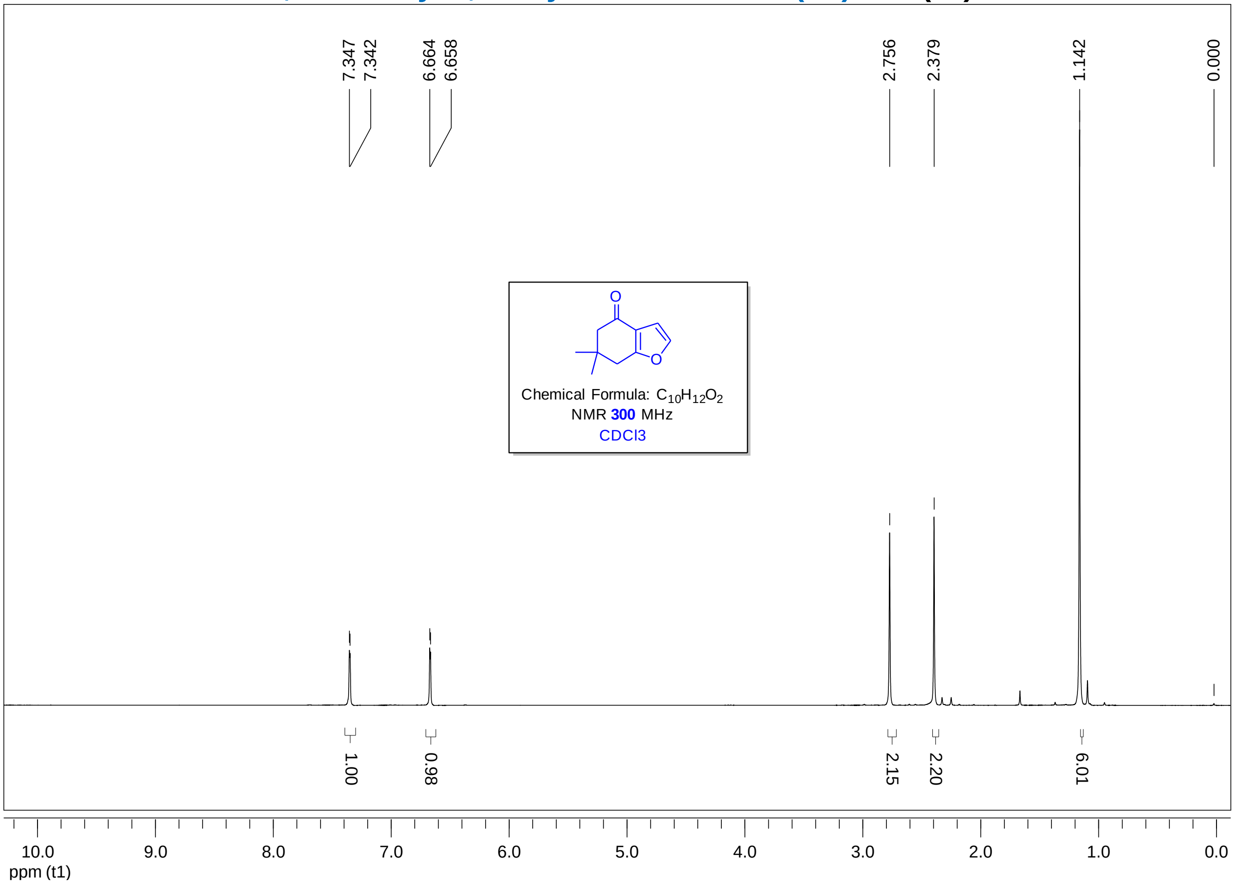

6,6-Dimethyl-6,7-dihydrobenzofuran-4(5*H*)-one (4c)

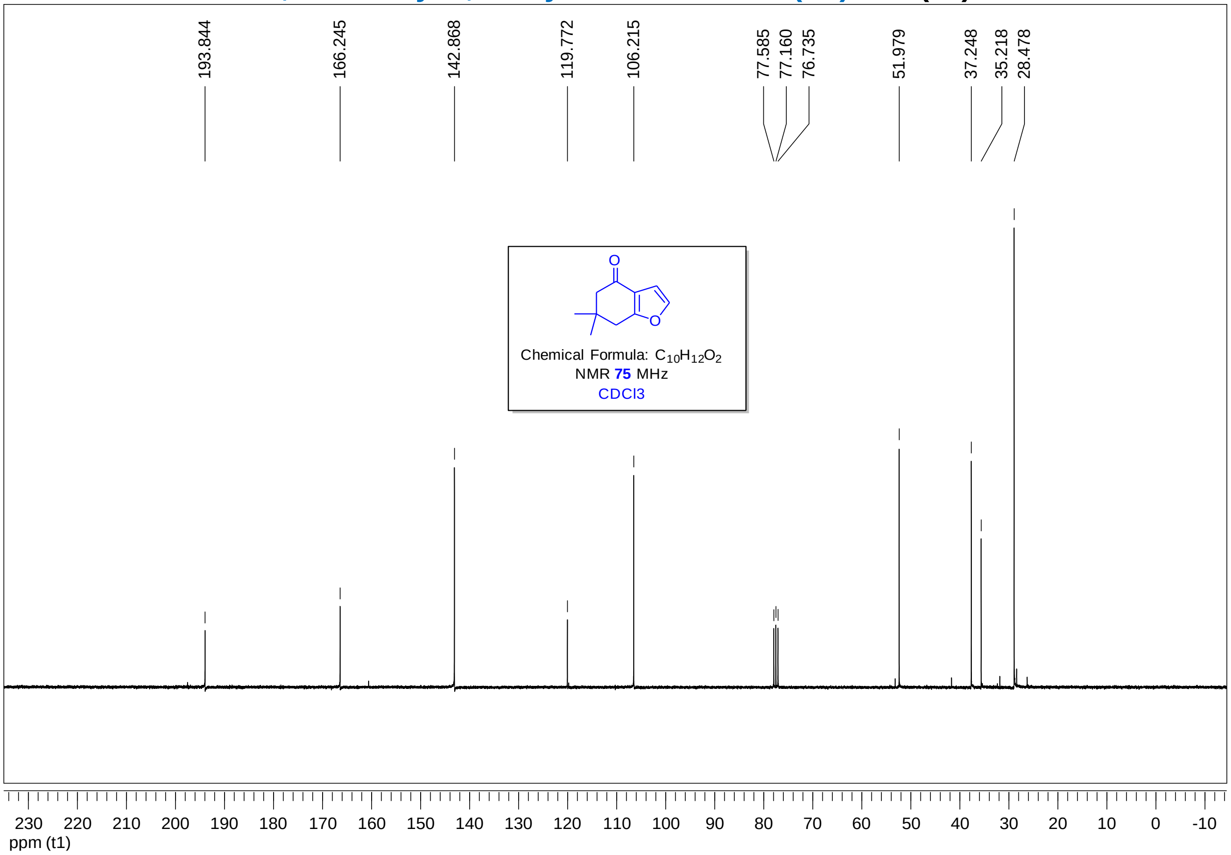

3,4,6,7,8,9-Hexahydrodibenzo[b,d]furan-1(2*H*)-one (4s)

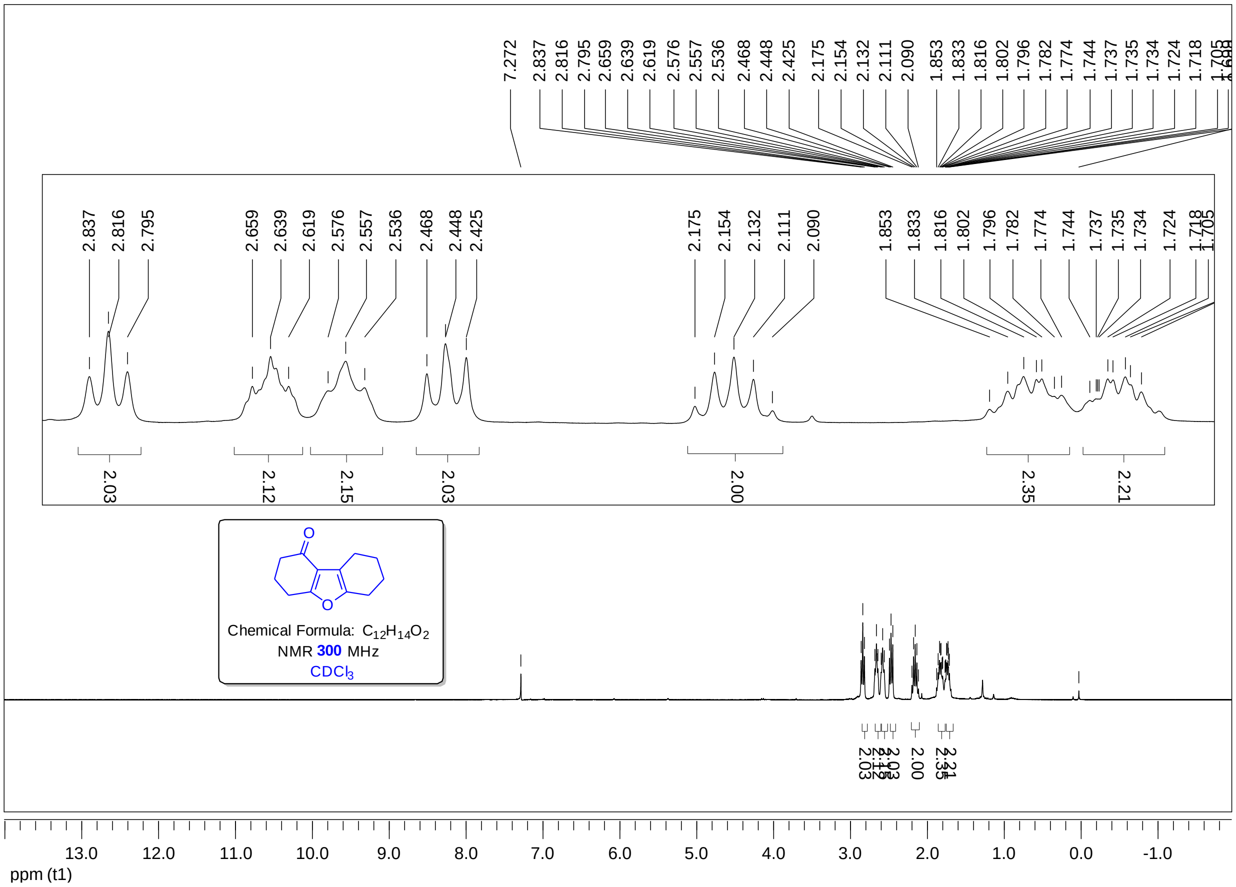

3,4,6,7,8,9-Hexahydrodibenzo[b,d]furan-1(2*H*)-one (4s)

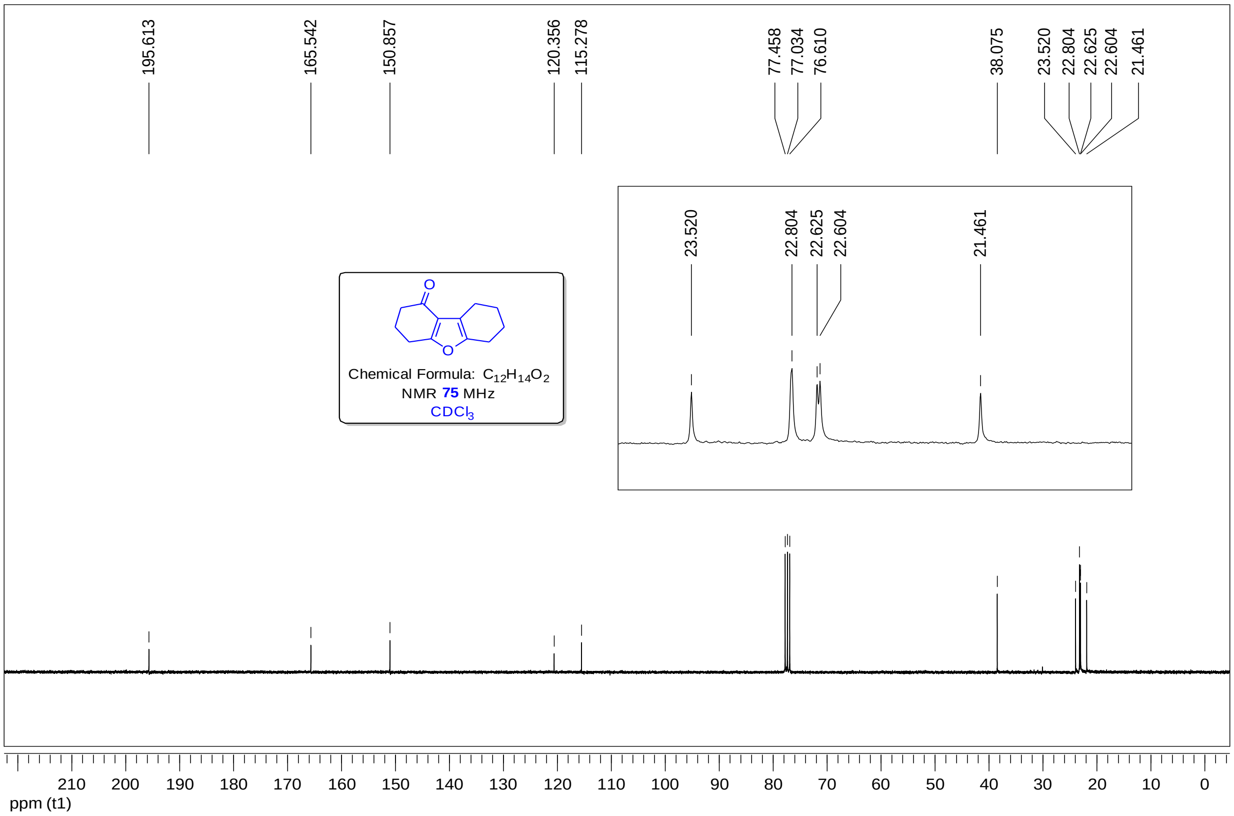

2,3-dihydro-1H-carbazol-4(9H)-one (3d)

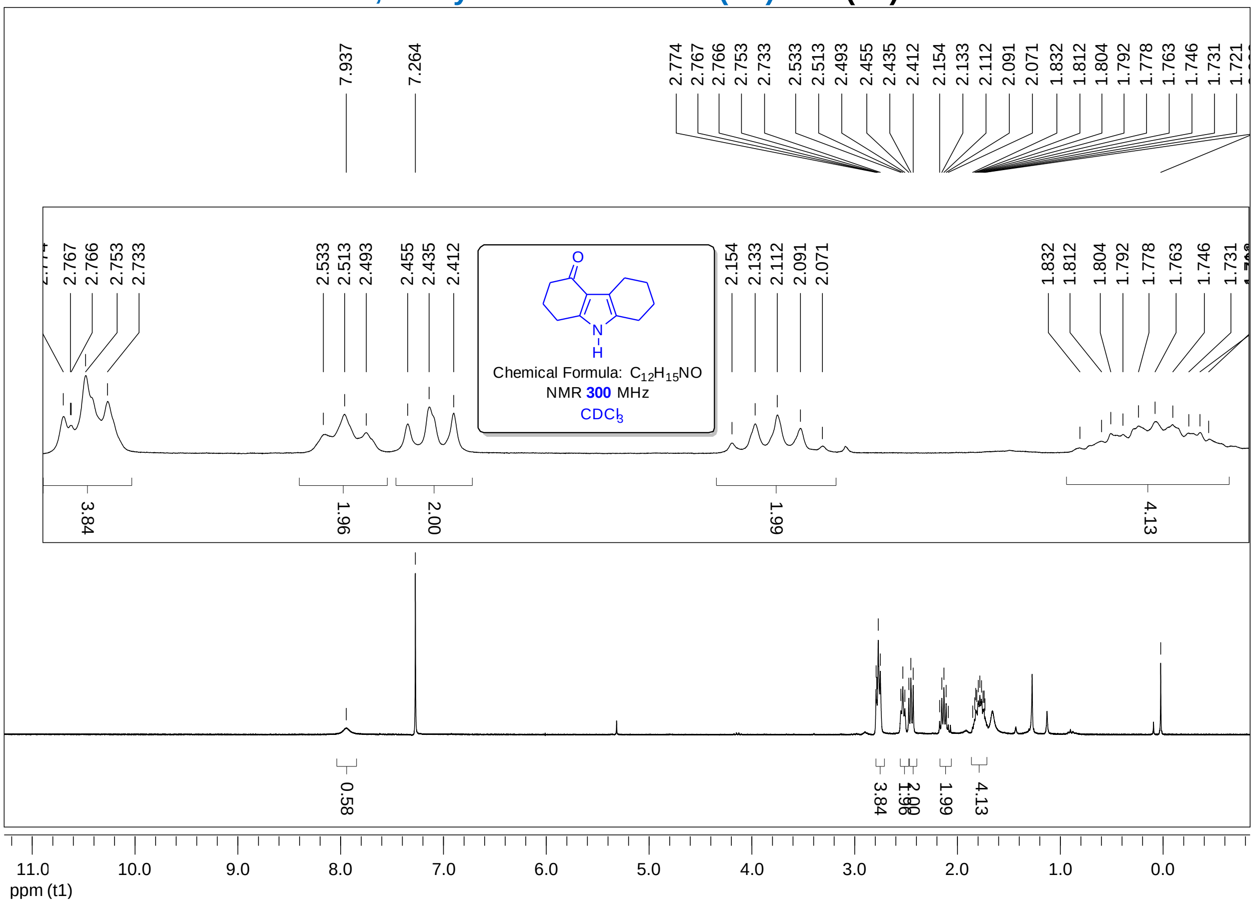

2,3-dihydro-1H-carbazol-4(9H)-one (3d)

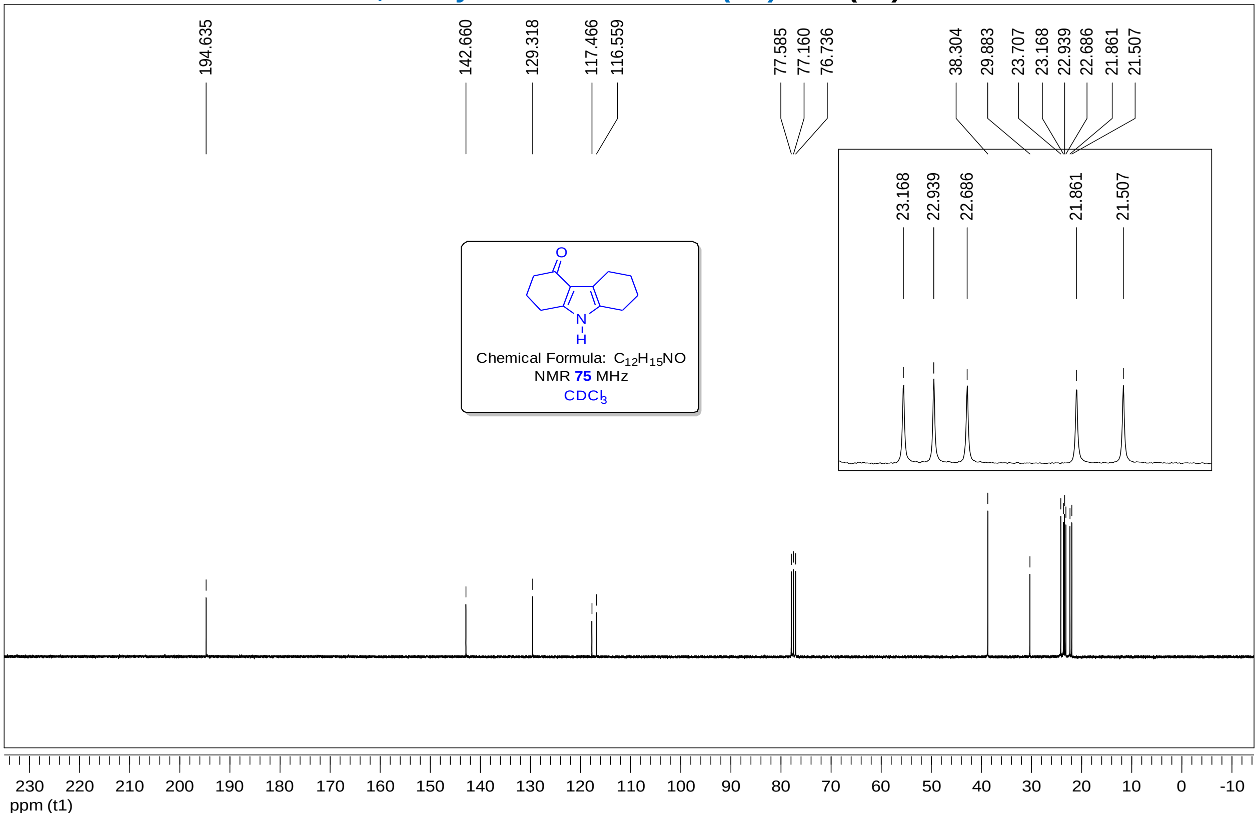

2,2-dimethyl-2,3-dihydro-1H-carbazol-4(9H)-one (3e)

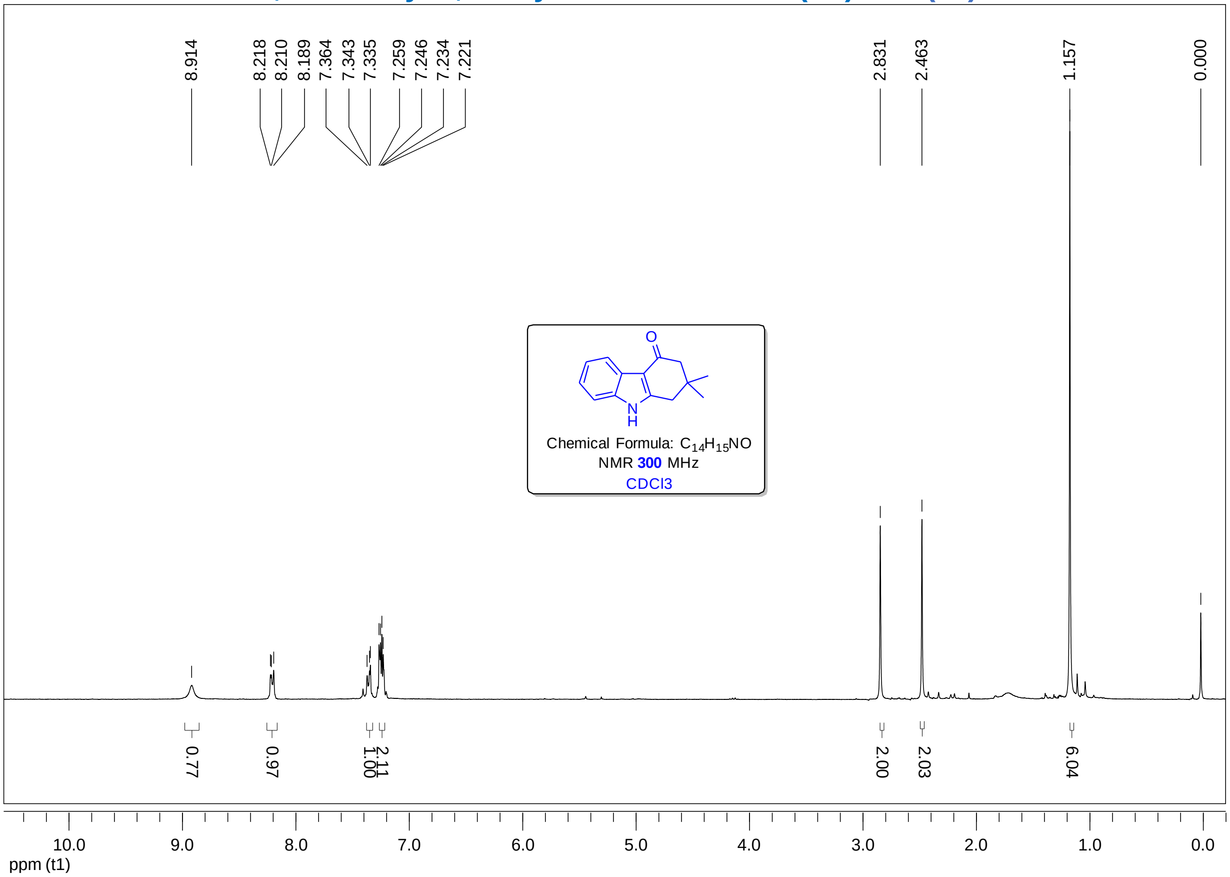

2,2-dimethyl-2,3-dihydro-1H-carbazol-4(9H)-one (3e)

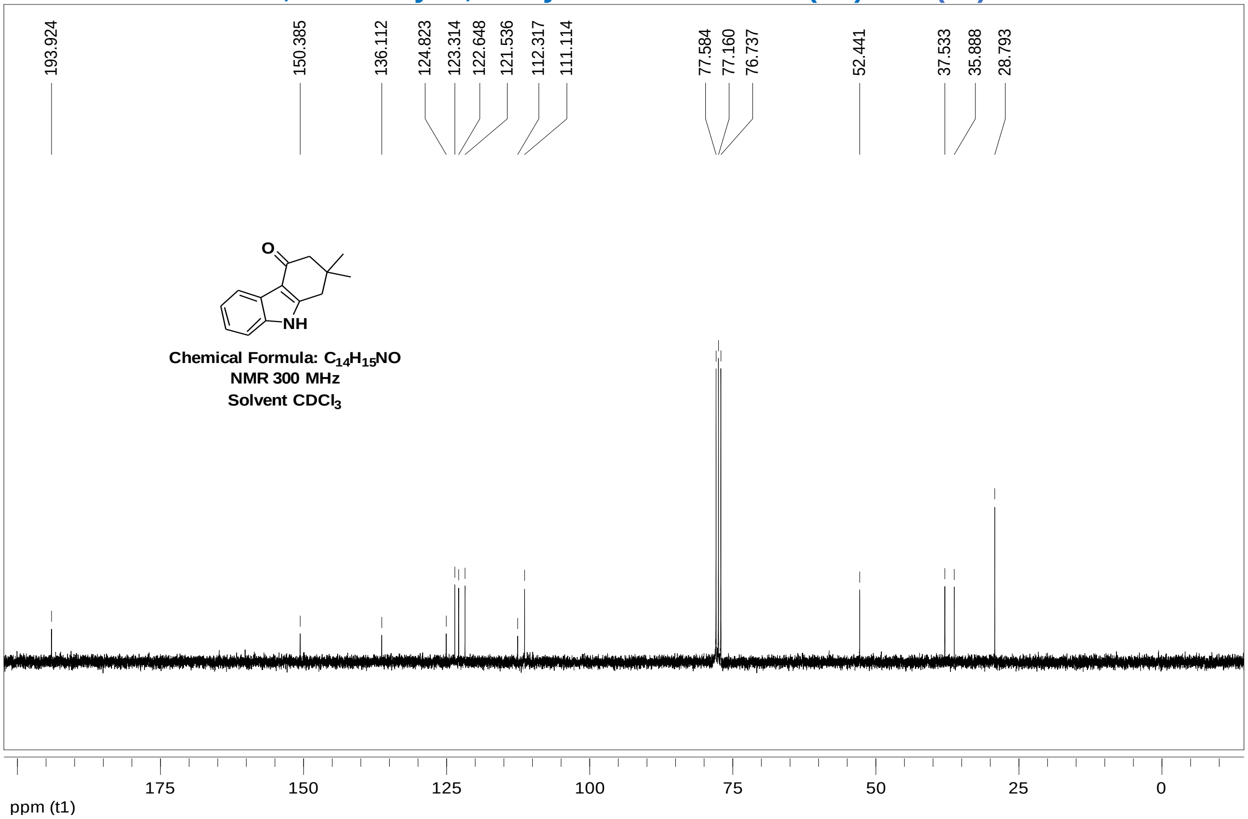

2,3,4,9-Tetrahydro-*1H*-carbazol-1-one (3f)

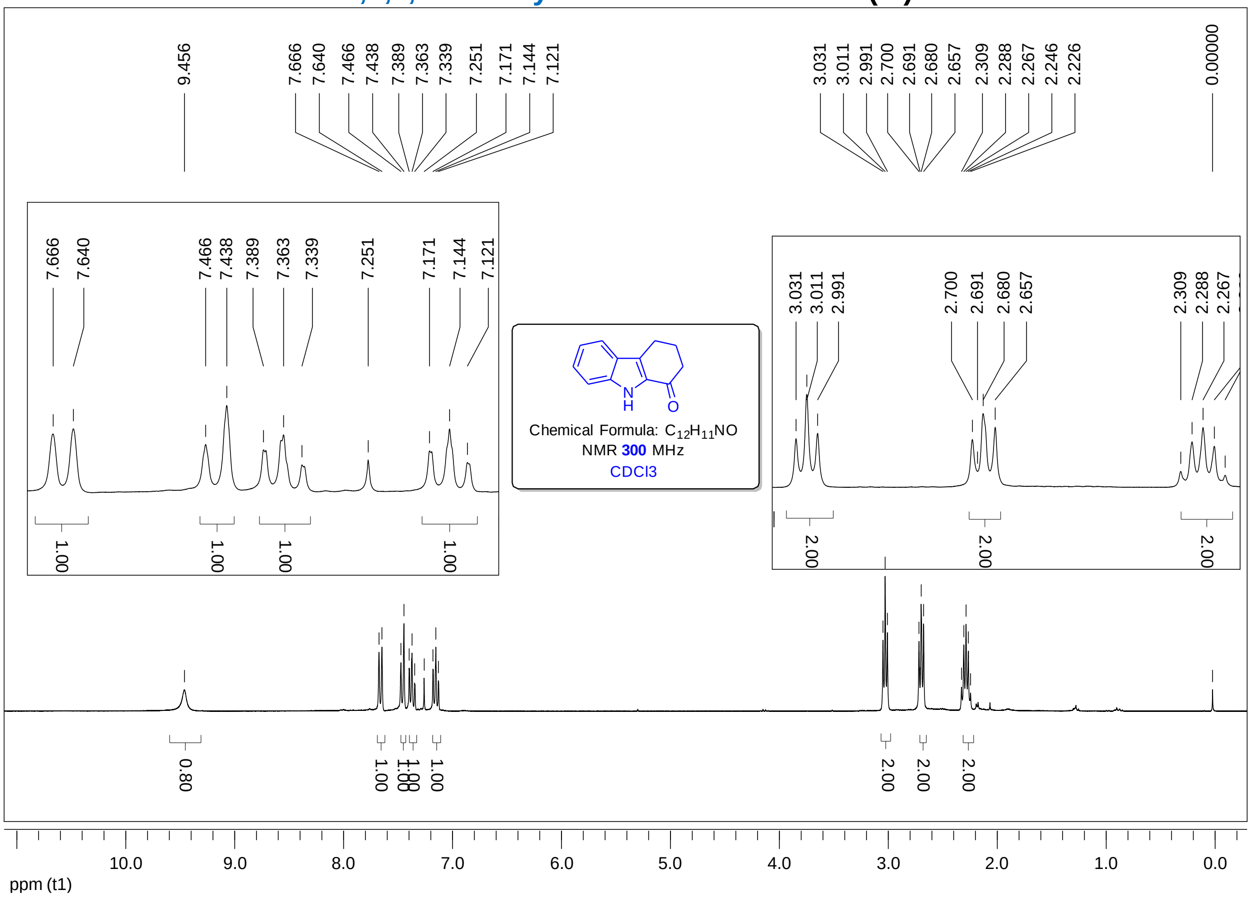

2,3,4,9-Tetrahydro-*1H*-carbazol-1-one (3f)

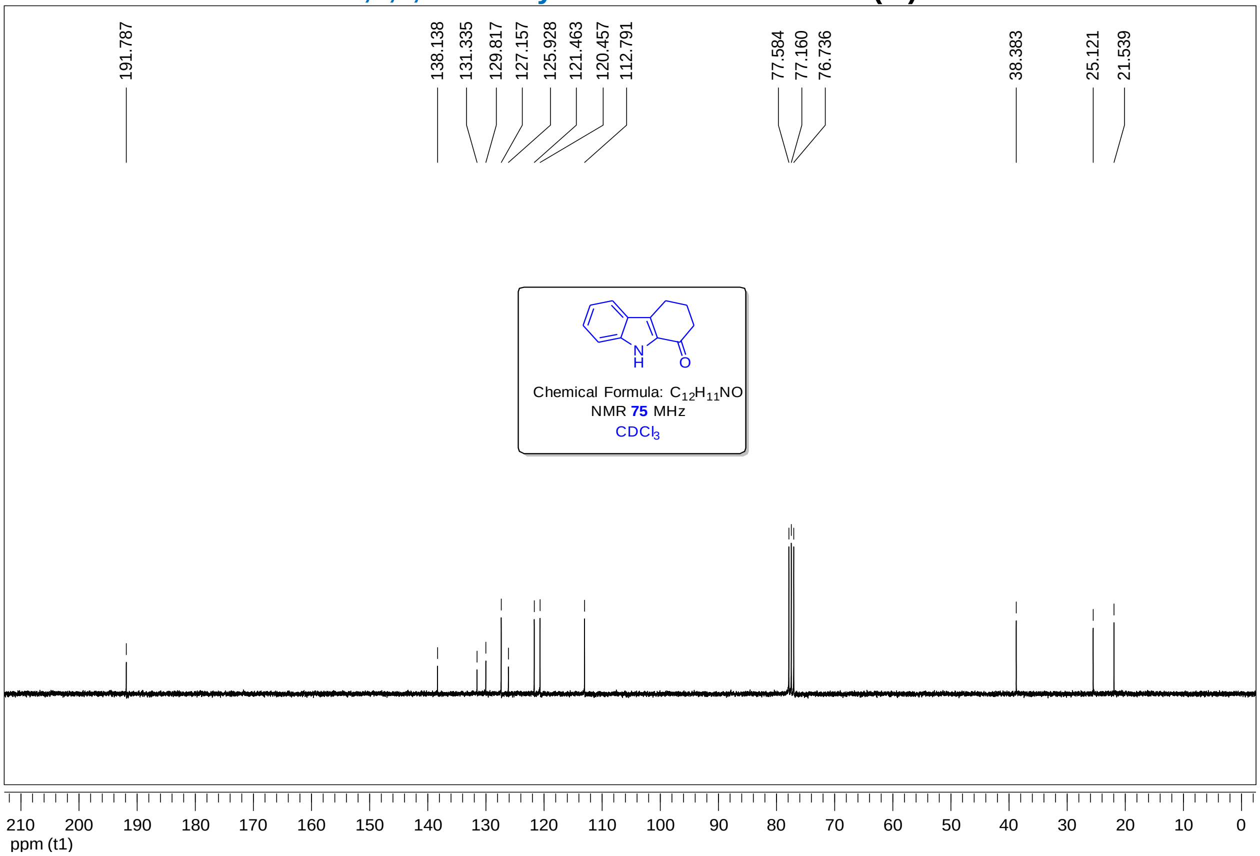

9-Methyl-1,2,3,5,6,7,8,9-octahydro-4*H*-carbazol-4-one 2 (4t)

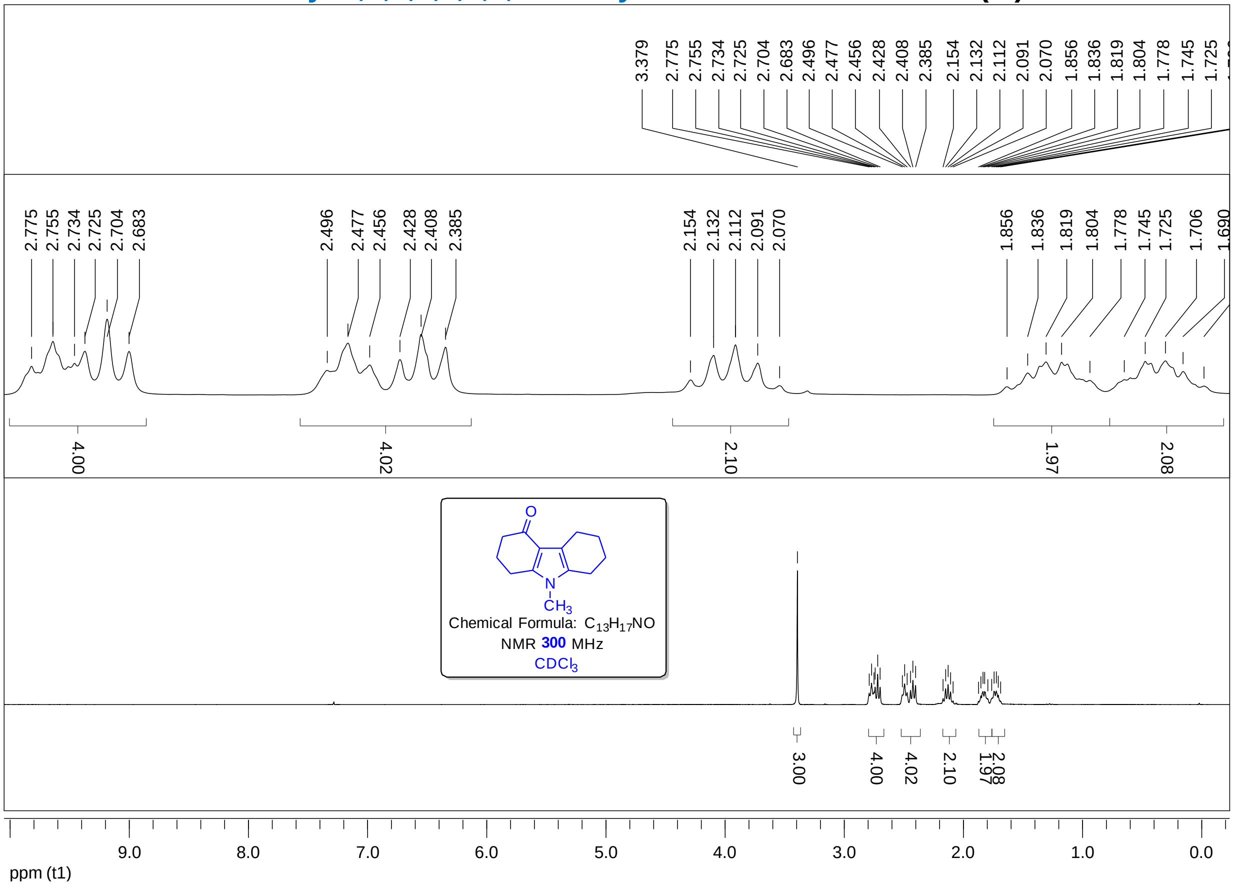

9-Methyl-1,2,3,5,6,7,8,9-octahydro-4*H*-carbazol-4-one 2 (4t)

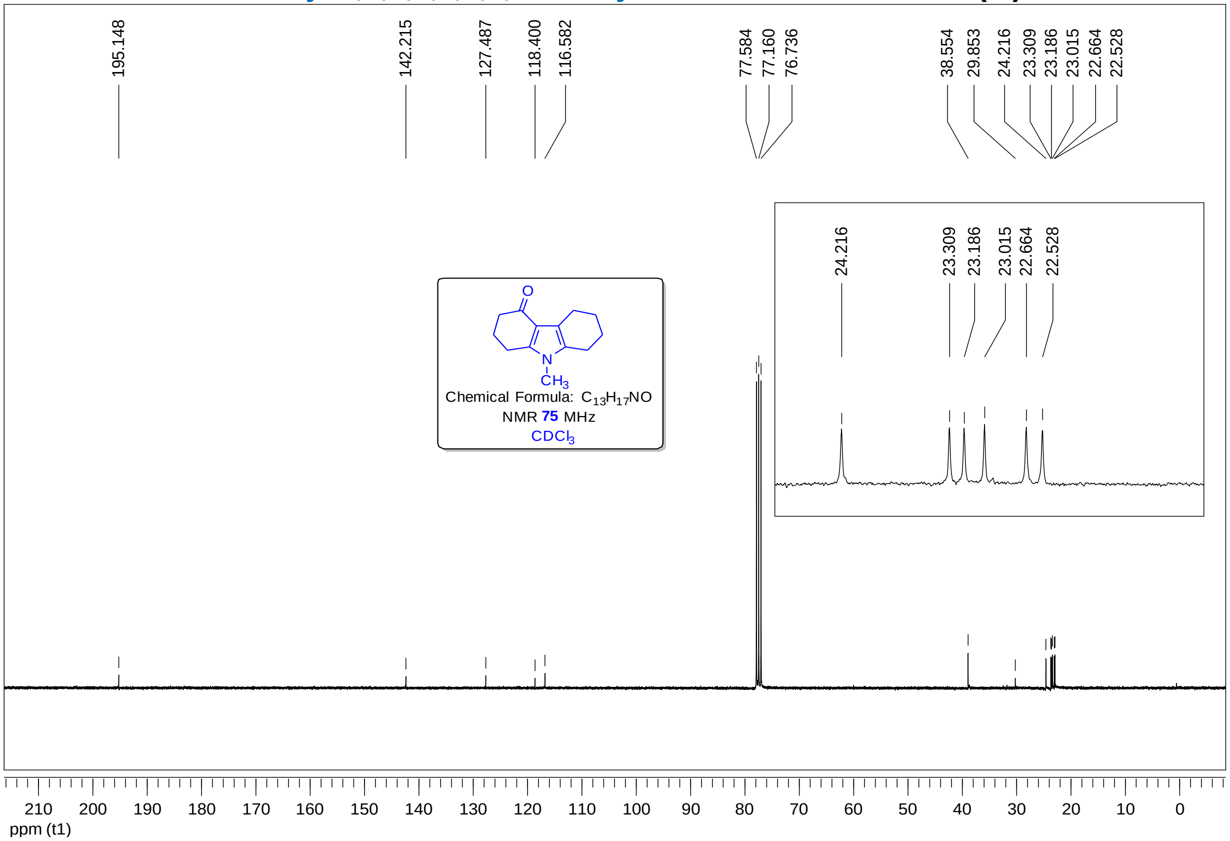

1-Methyl-1,5,6,7-tetrahydro-4H-indol-4-one (4u)

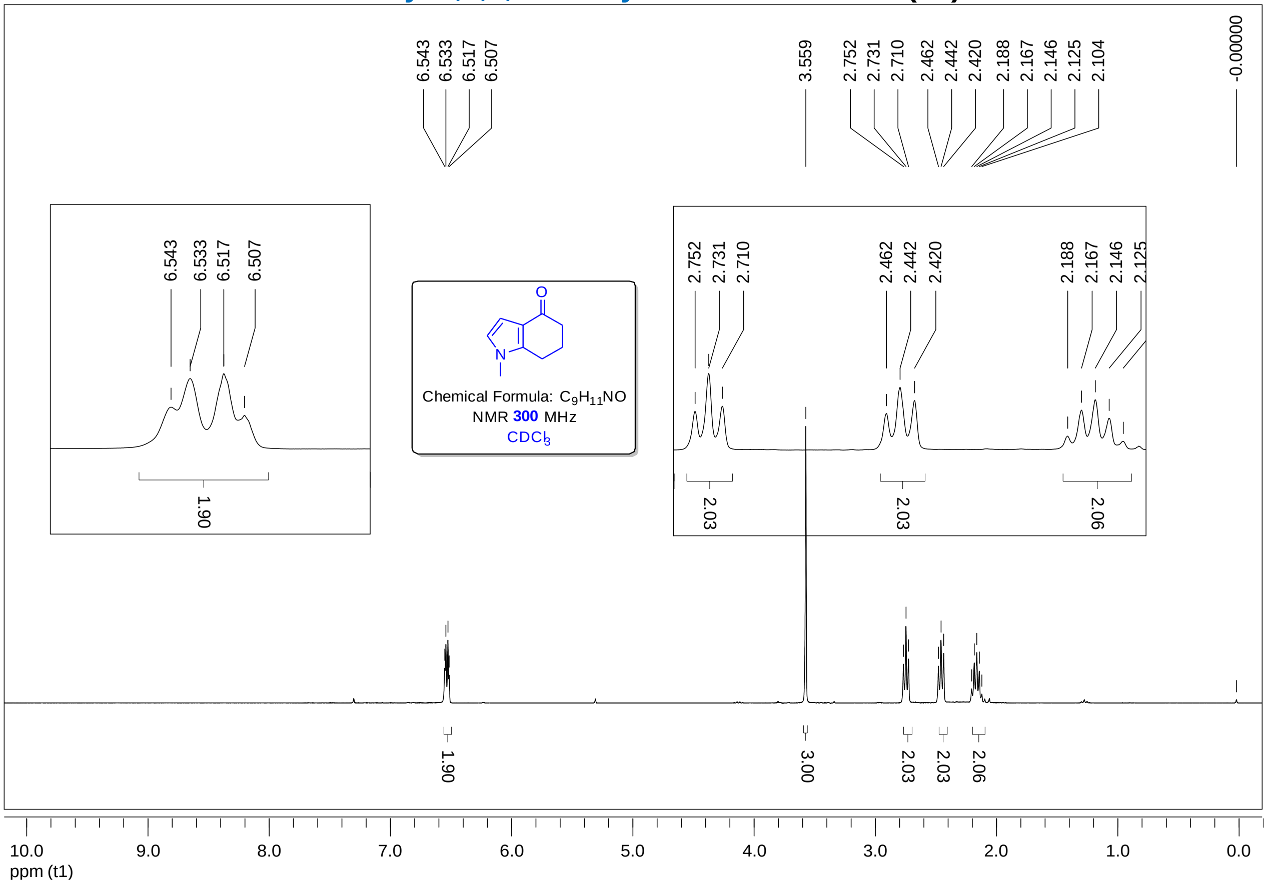

1-Methyl-1,5,6,7-tetrahydro-4H-indol-4-one (4u)

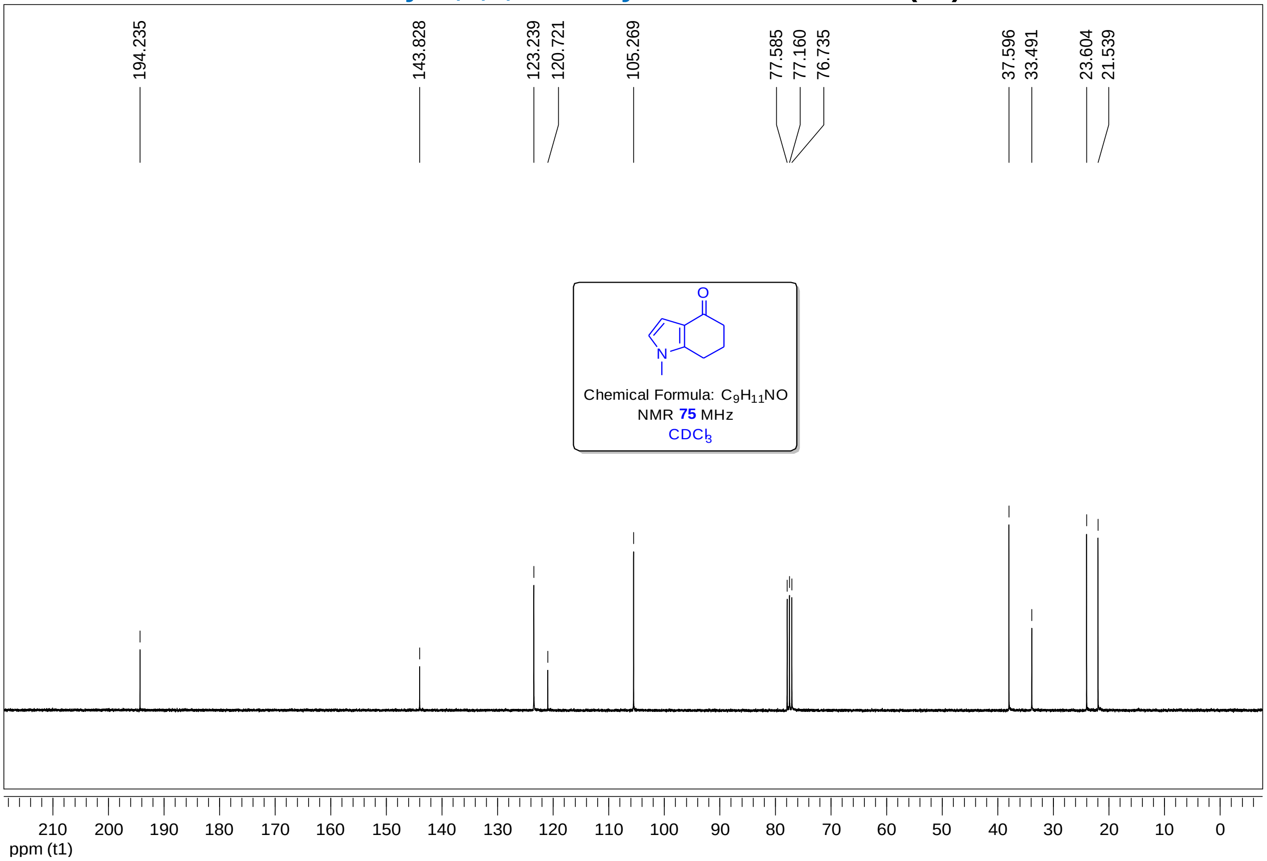

1-(3-Chlorophenyl)-1,5,6,7-tetrahydro-4*H*-indol-4-one (4v)

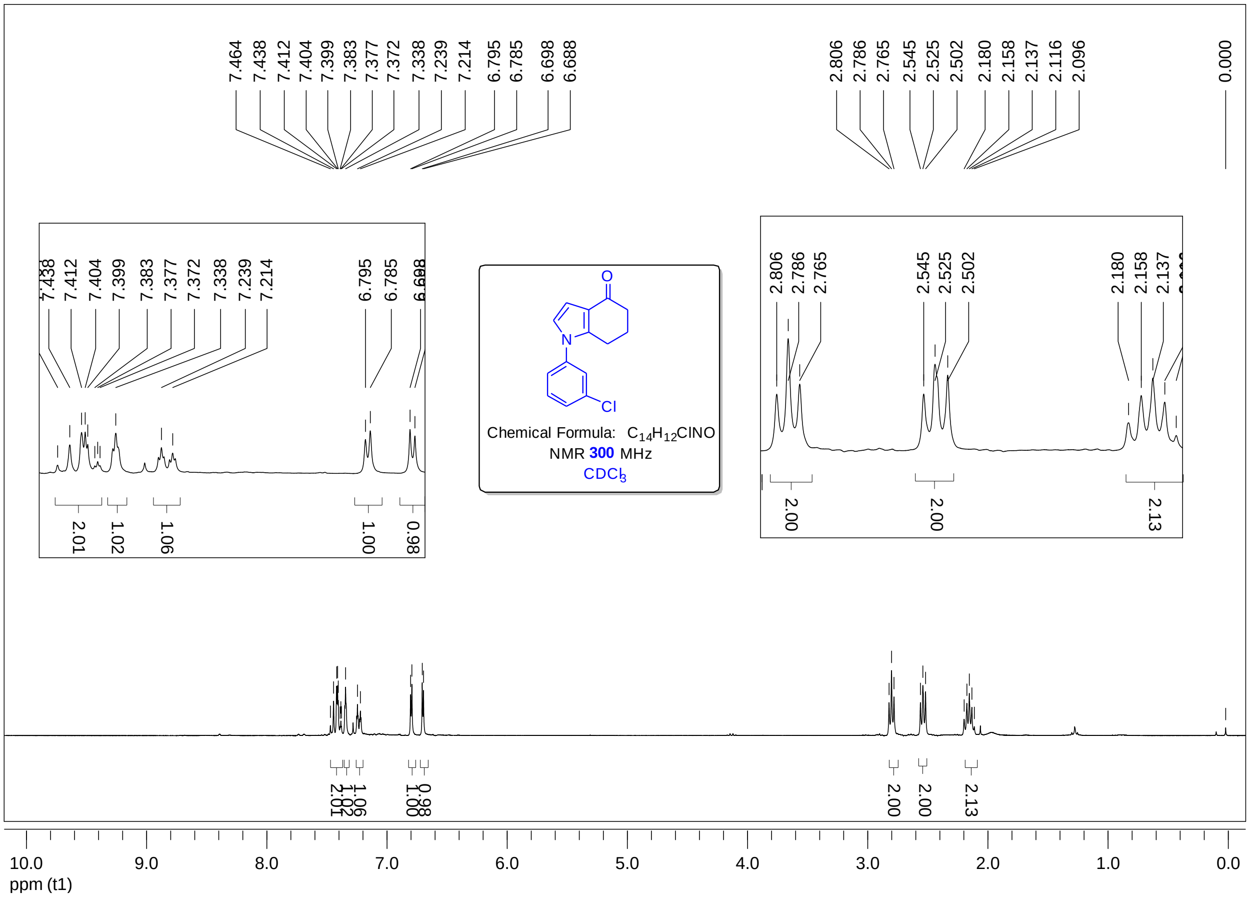

1-(3-Chlorophenyl)-1,5,6,7-tetrahydro-4*H*-indol-4-one (4v)

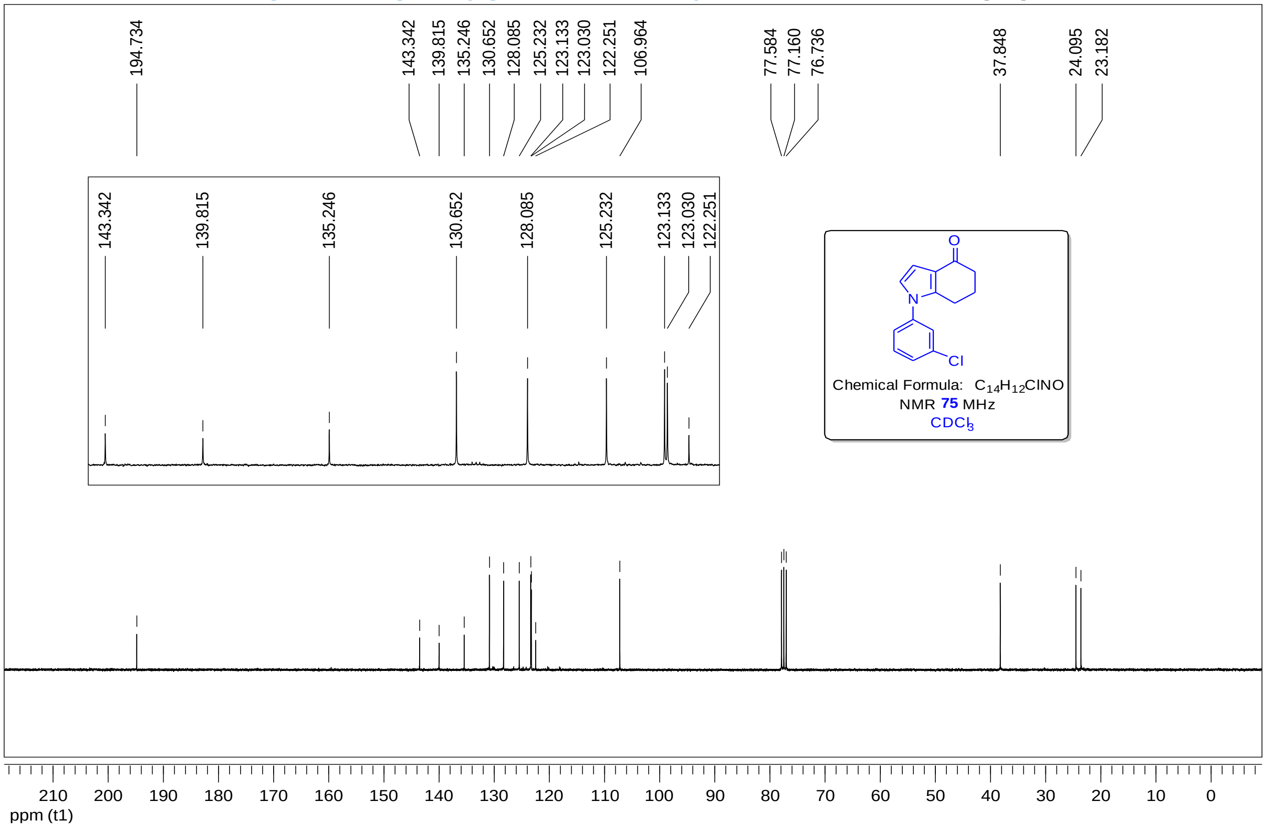

1,5,5-Trimethyl-1,5,6,7-tetrahydro-4*H*-indol-4-one (4w)

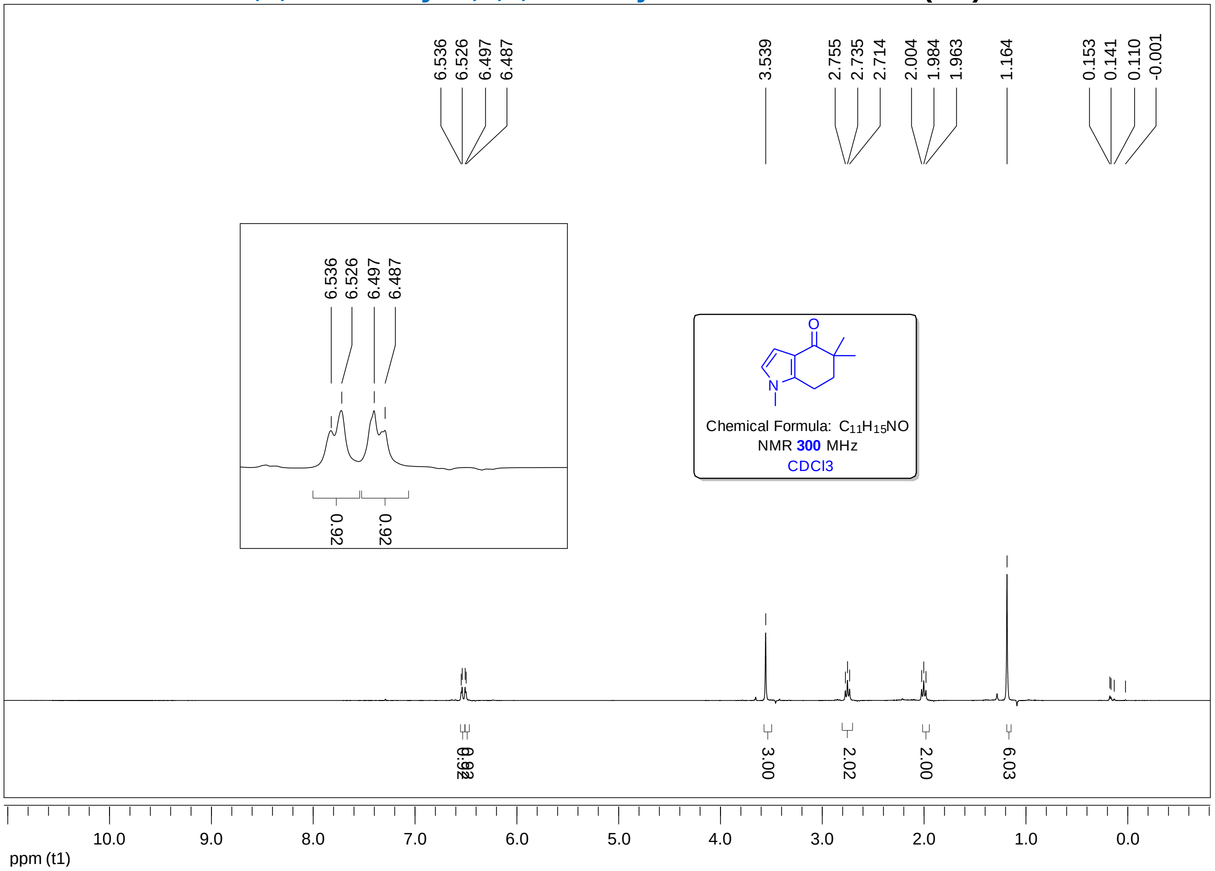

1,5,5-Trimethyl-1,5,6,7-tetrahydro-4*H*-indol-4-one (4w)

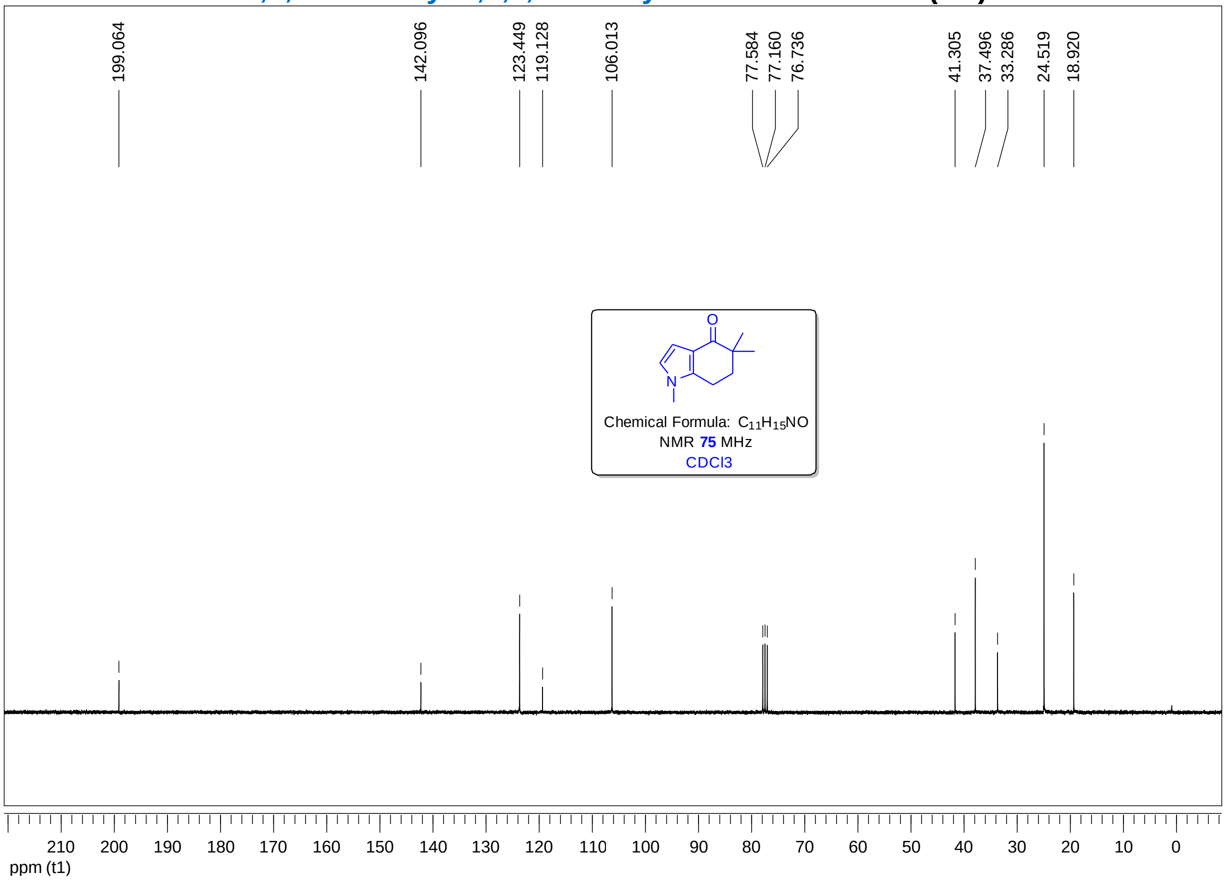

6,7-Dihydroisoquinolin-8(5*H*)-one (4k)

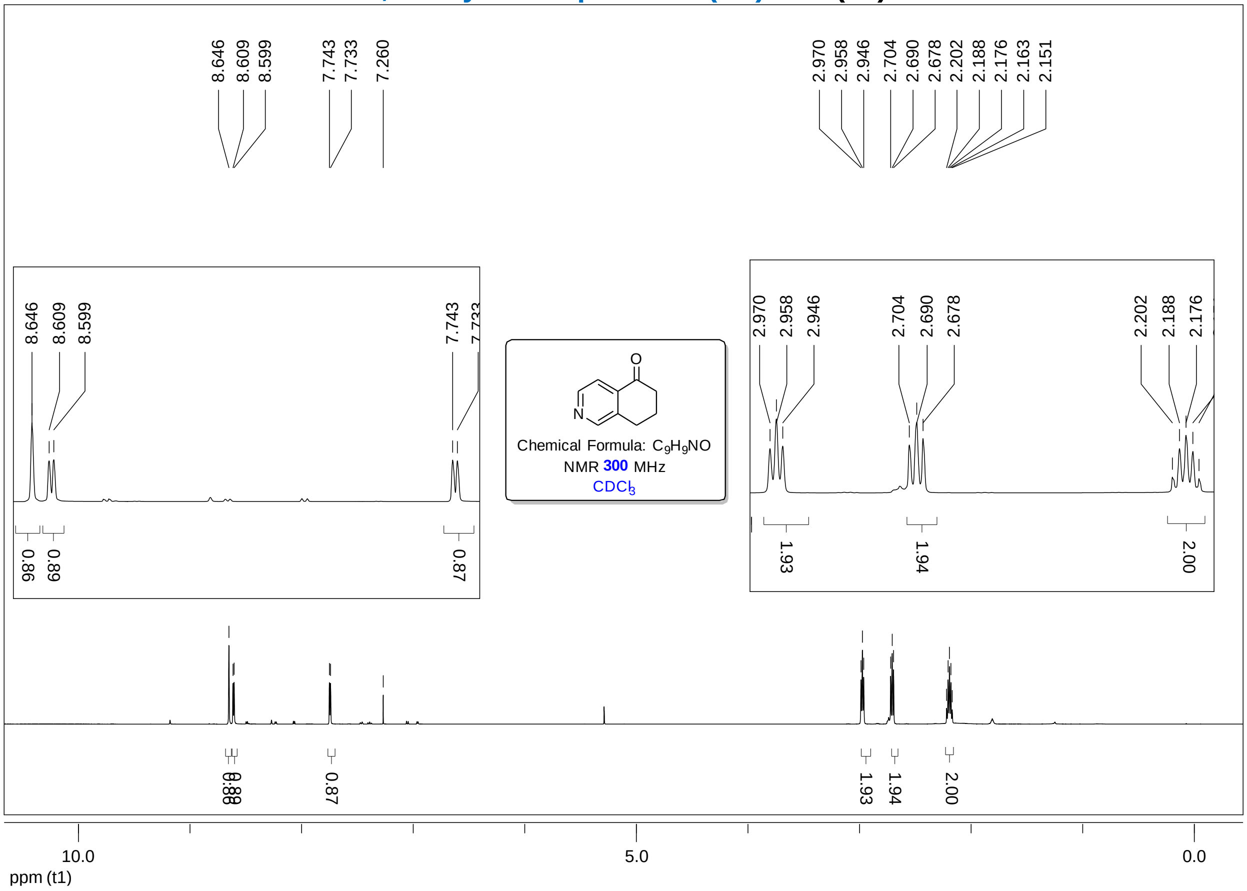

6,7-Dihydroisoquinolin-8(5*H*)-one (4k)

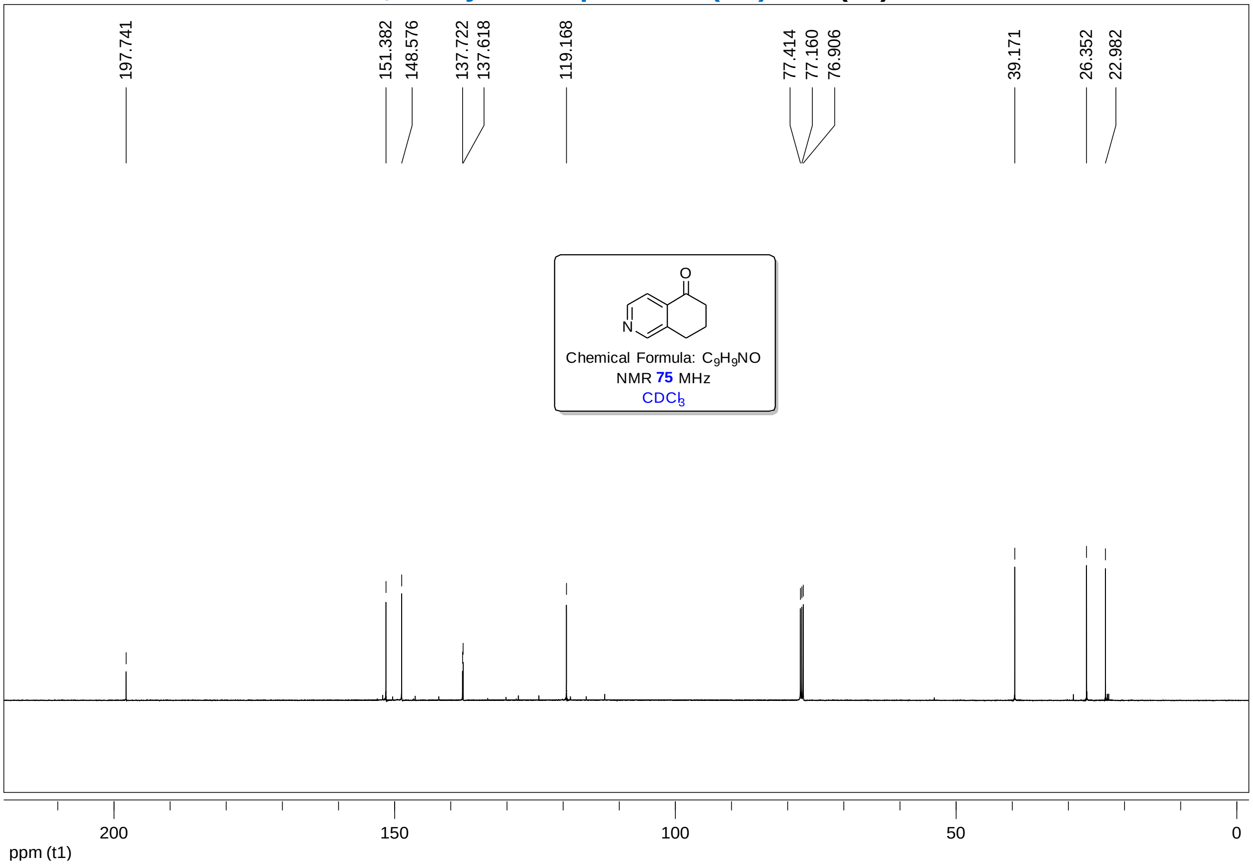

7,8-dihydroisoquinolin-5(6*H*)-one (4j)

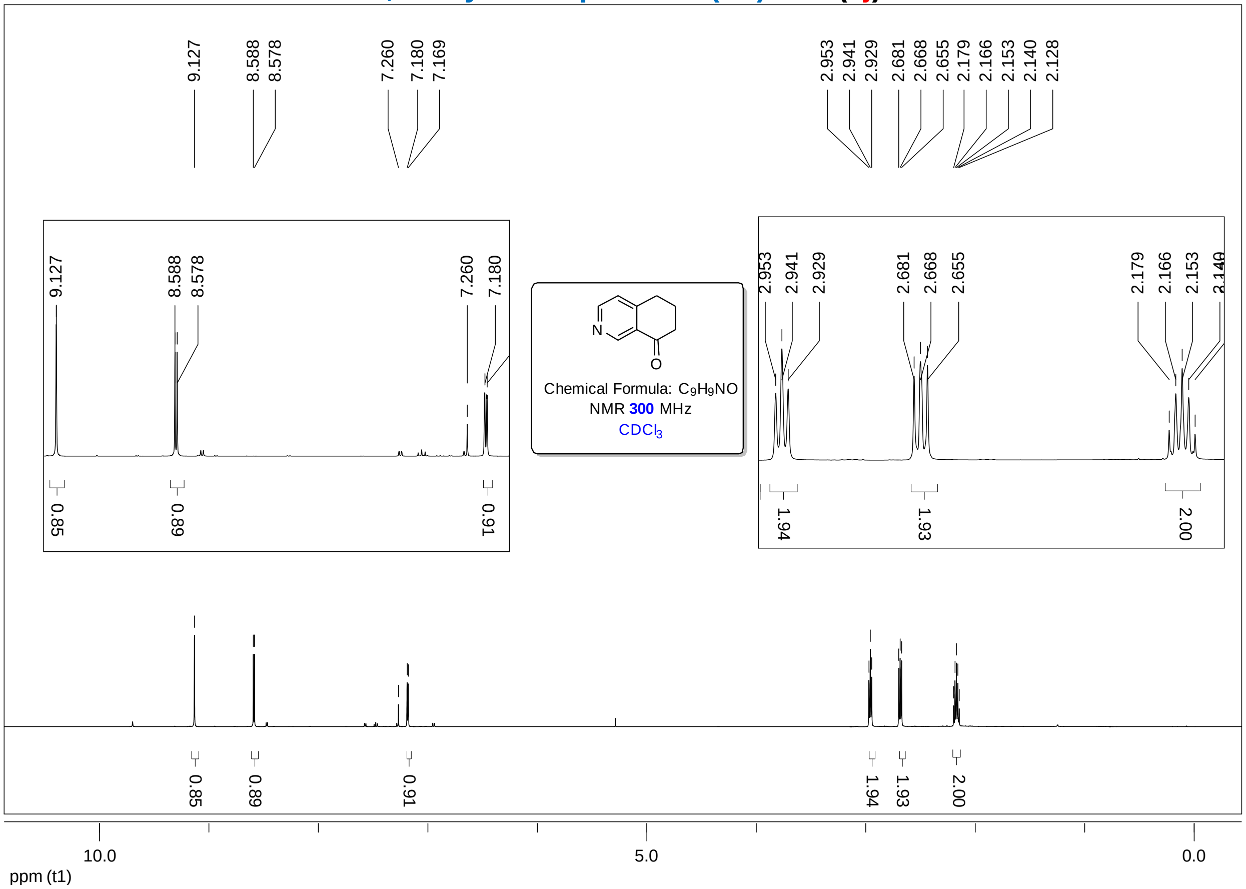

7,8-dihydroisoquinolin-5(6*H*)-one (4j)

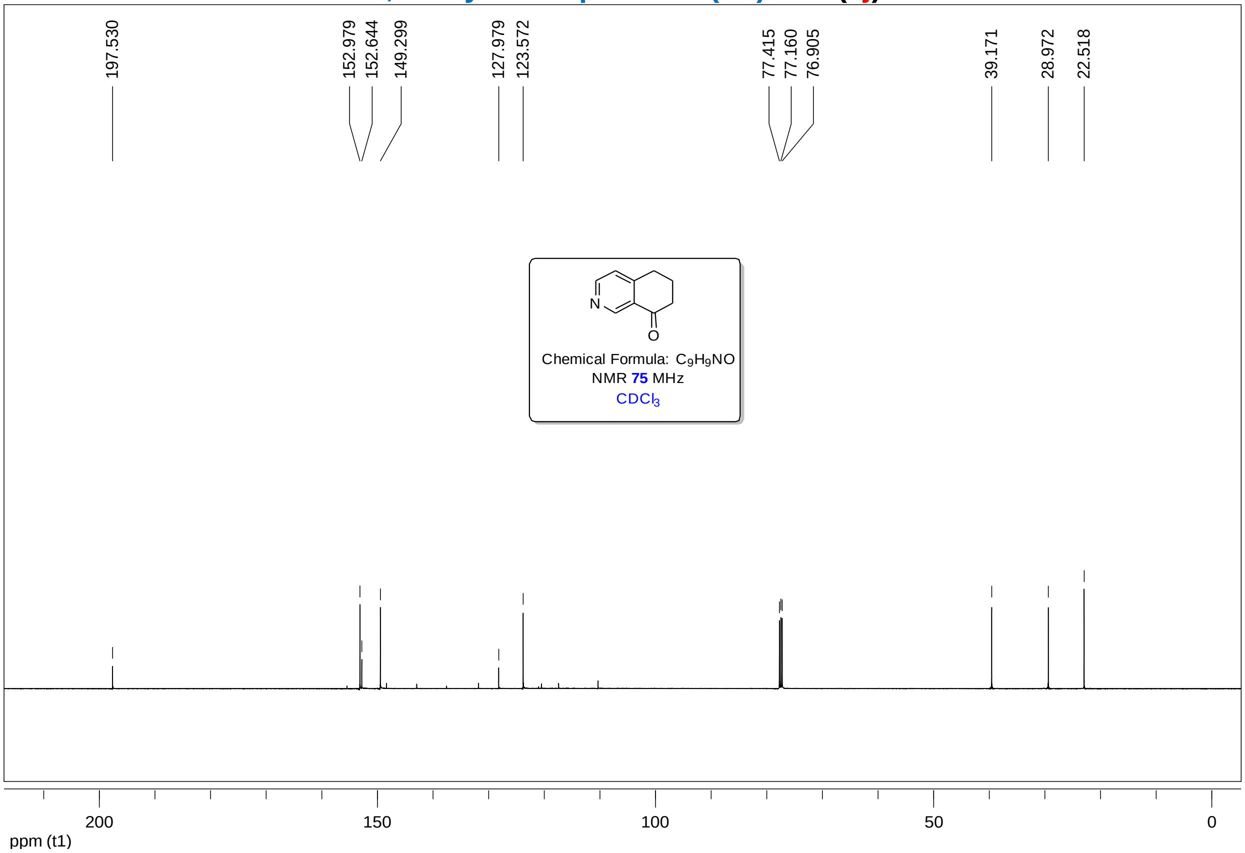

9-Acetyl-1,2,3,9-tetrahydro-4*H*-carbazol-4-one (**4d**)

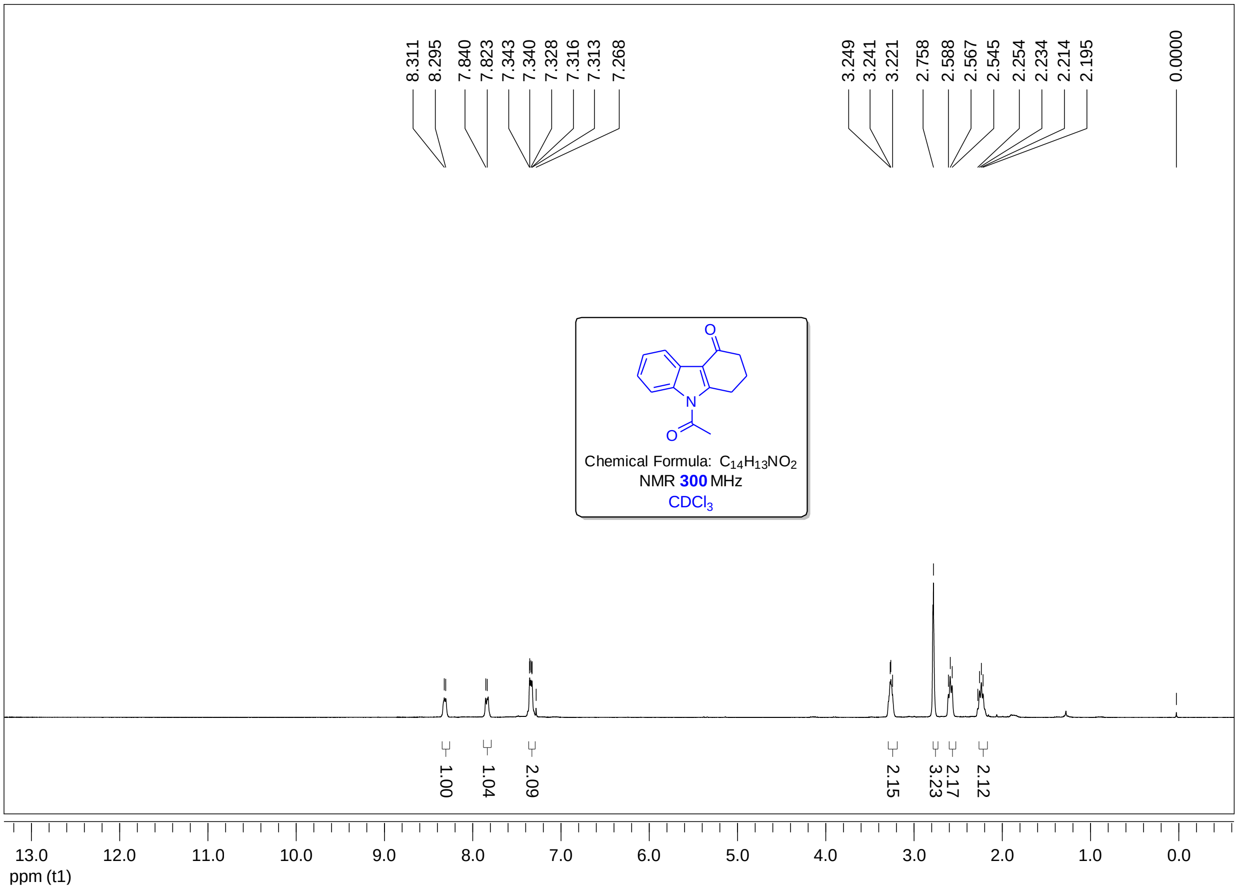

9-Acetyl-1,2,3,9-tetrahydro-4*H*-carbazol-4-one (**4d**)

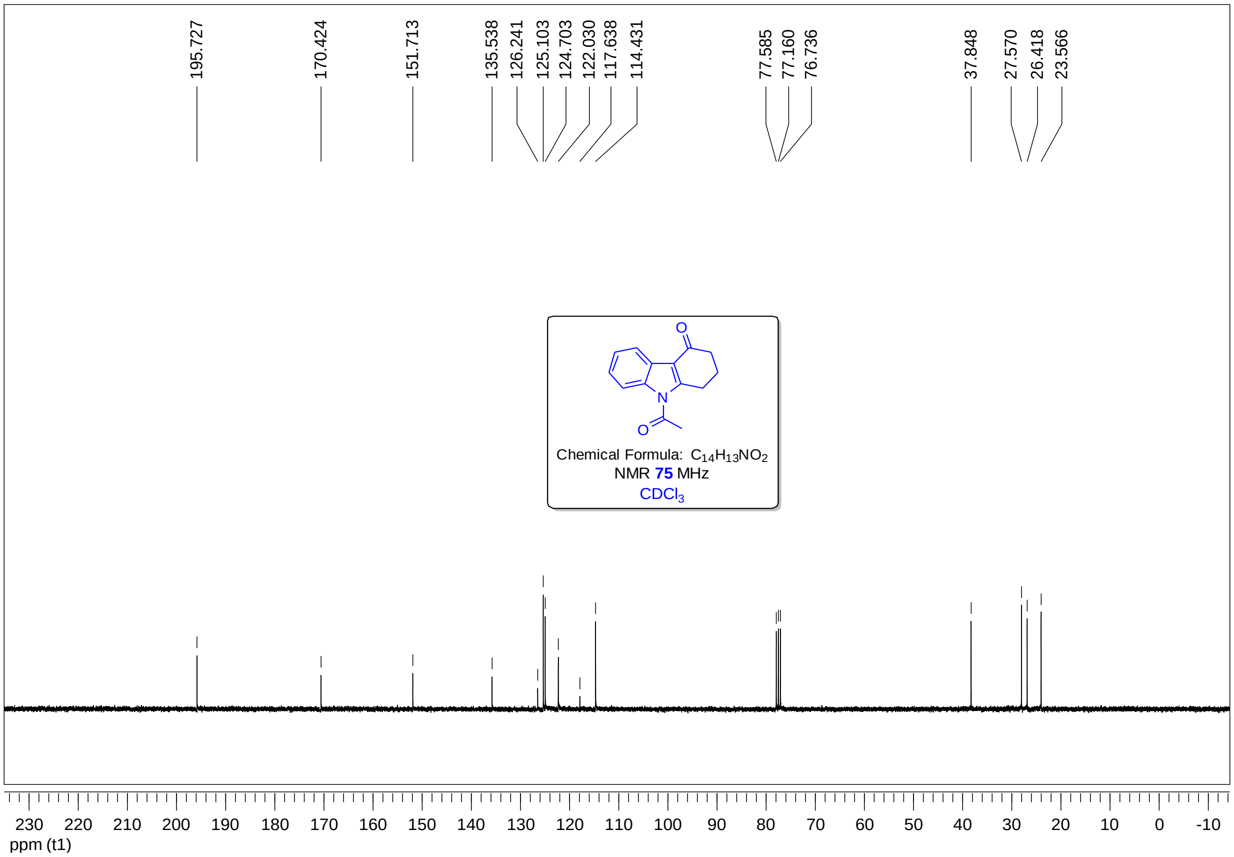

9-Tosyl-1,2,3,9-tetrahydro-4*H*-carbazol-4-one (4e)

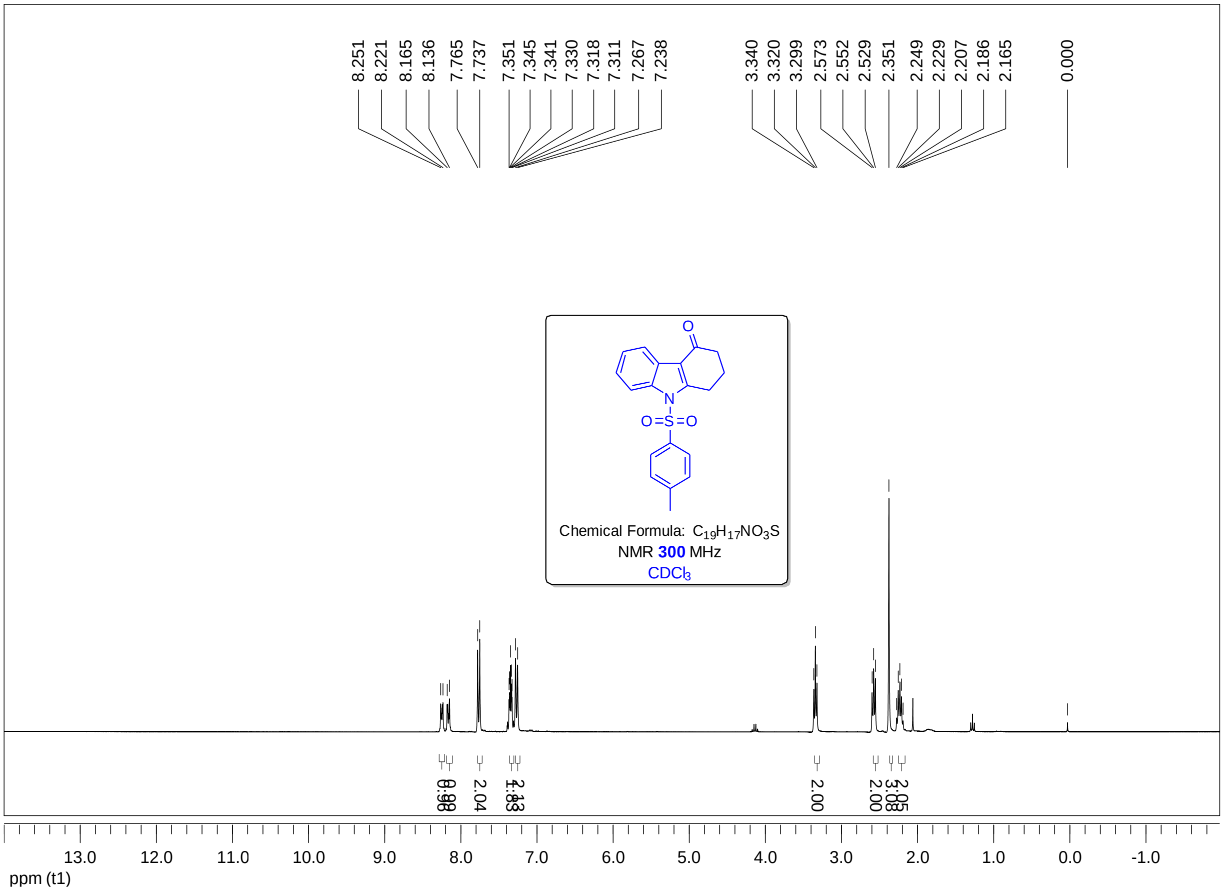

9-Tosyl-1,2,3,9-tetrahydro-4*H*-carbazol-4-one (4e)

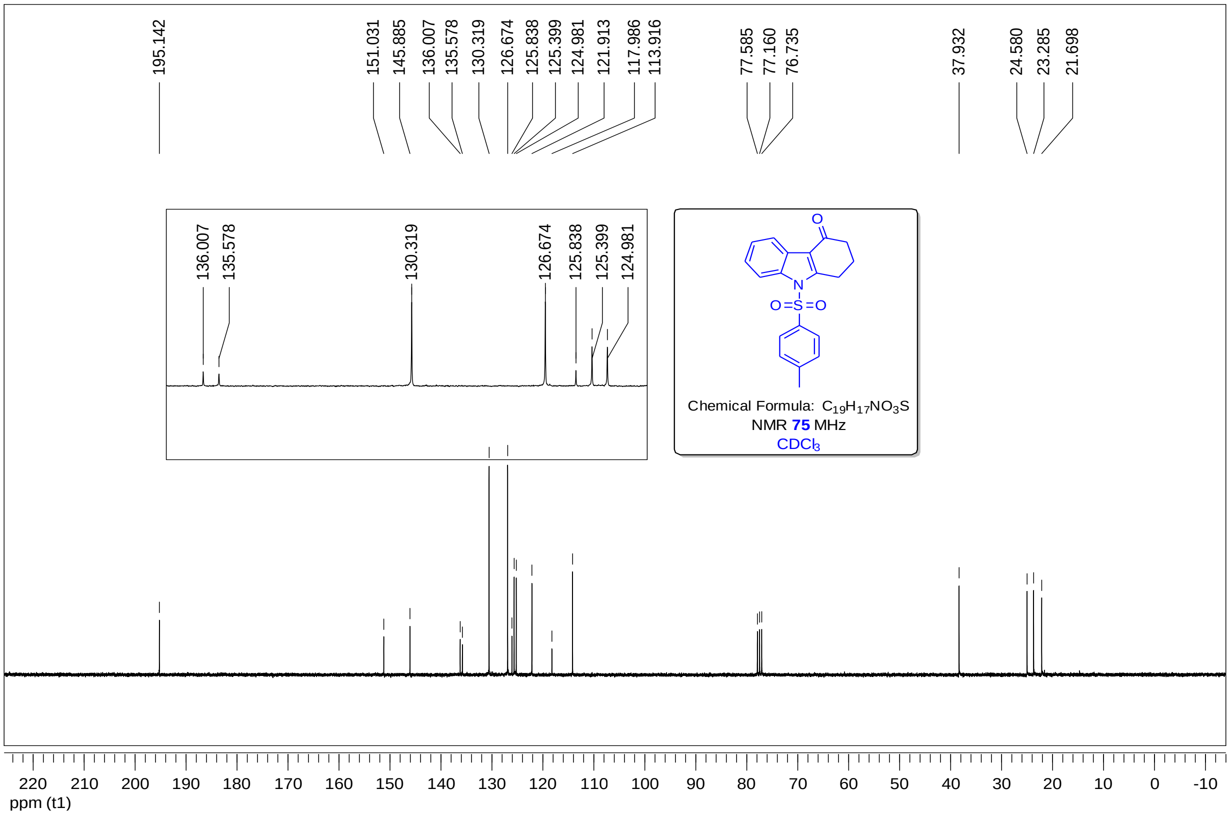

9-Acetyl-2,2-dimethyl-1,2,3,9-tetrahydro-4*H*-carbazol-4-one (4f)

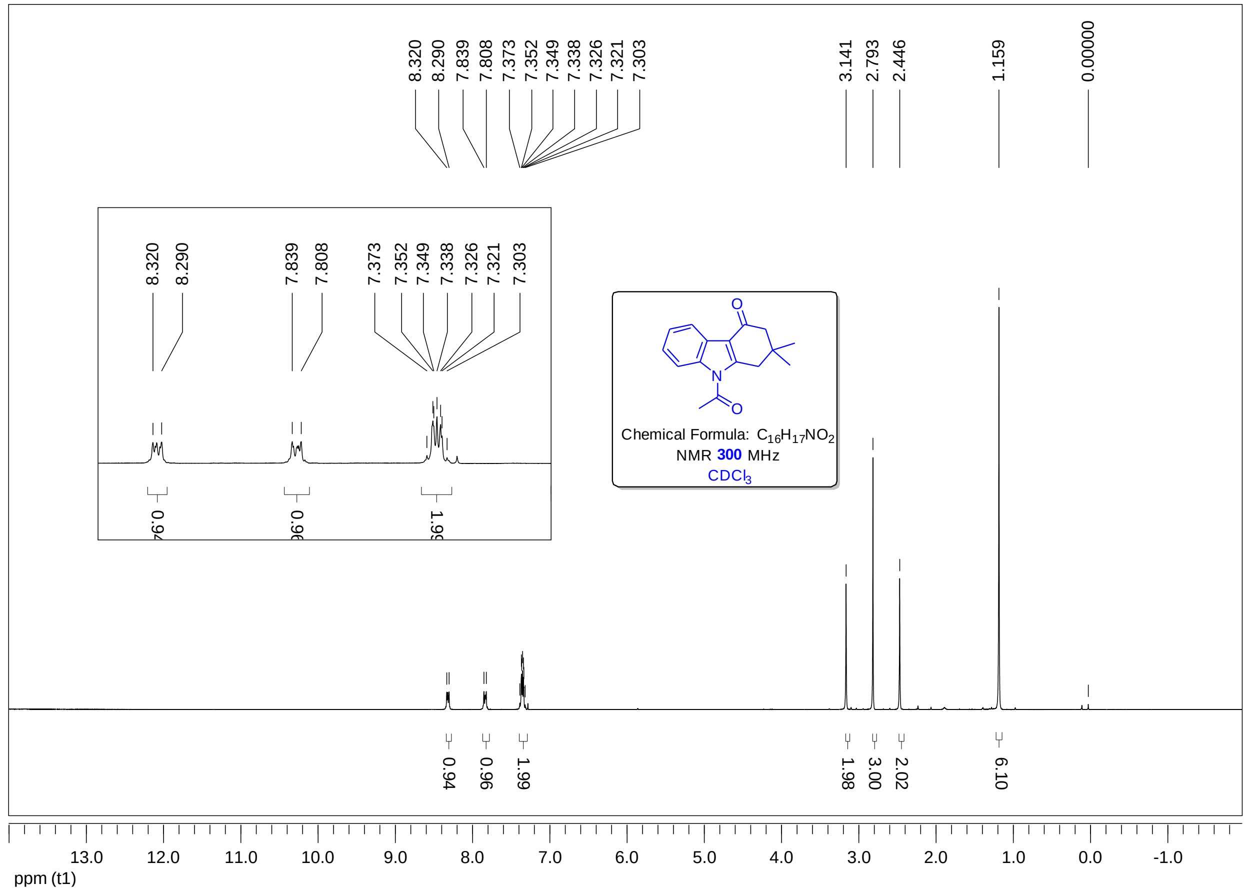

9-Acetyl-2,2-dimethyl-1,2,3,9-tetrahydro-4*H*-carbazol-4-one (4f)

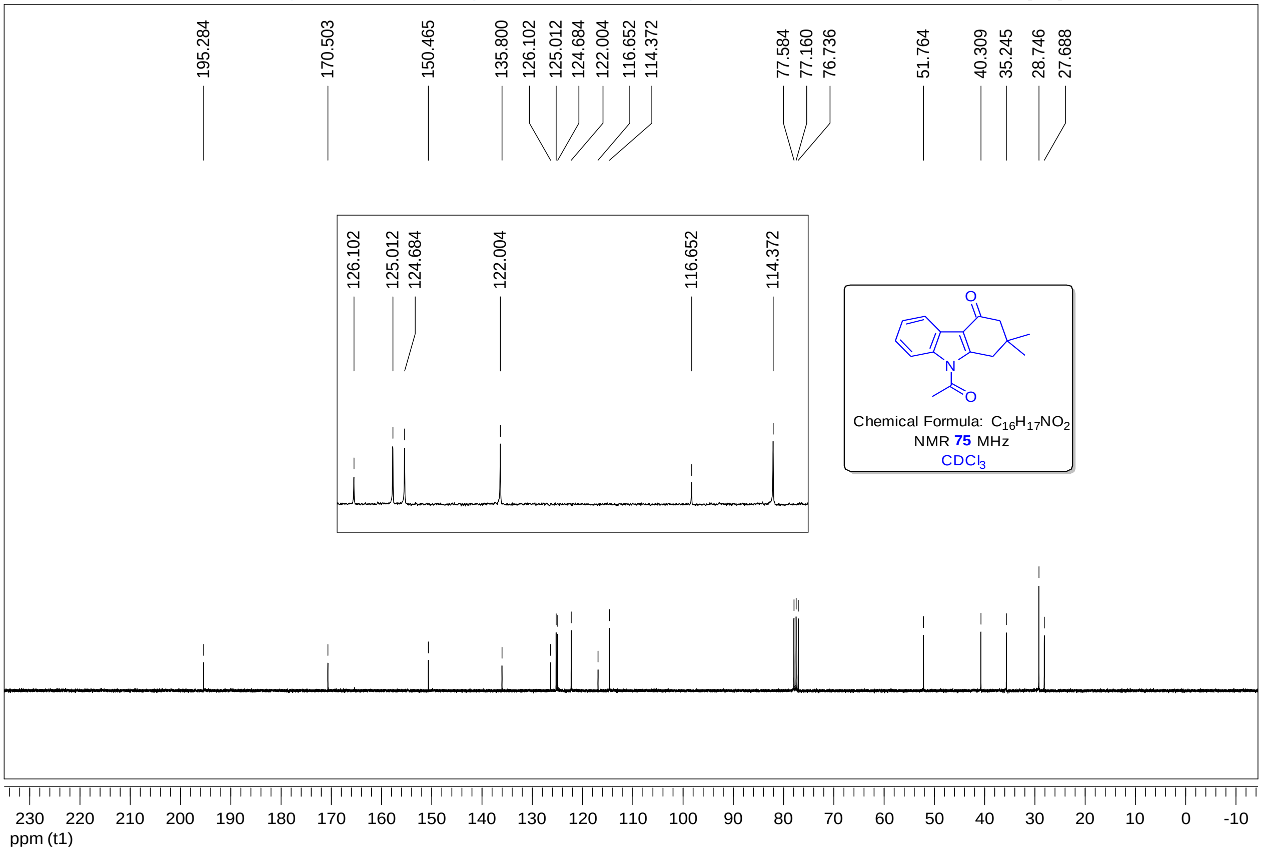

2,2-Dimethyl-9-tosyl-1,2,3,9-tetrahydro-*4H*-carbazol-4-one (4g)

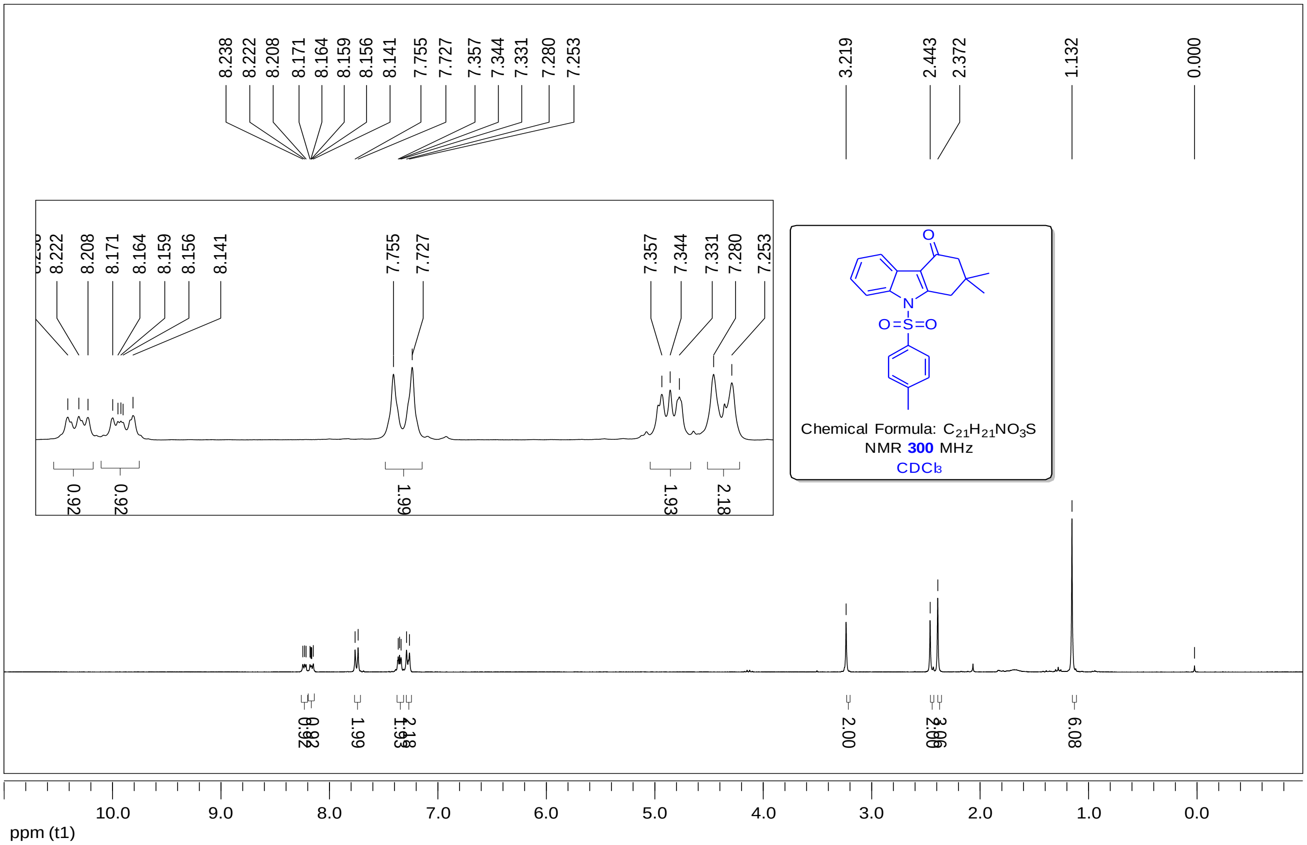

2,2-Dimethyl-9-tosyl-1,2,3,9-tetrahydro-*4H*-carbazol-4-one (4g)

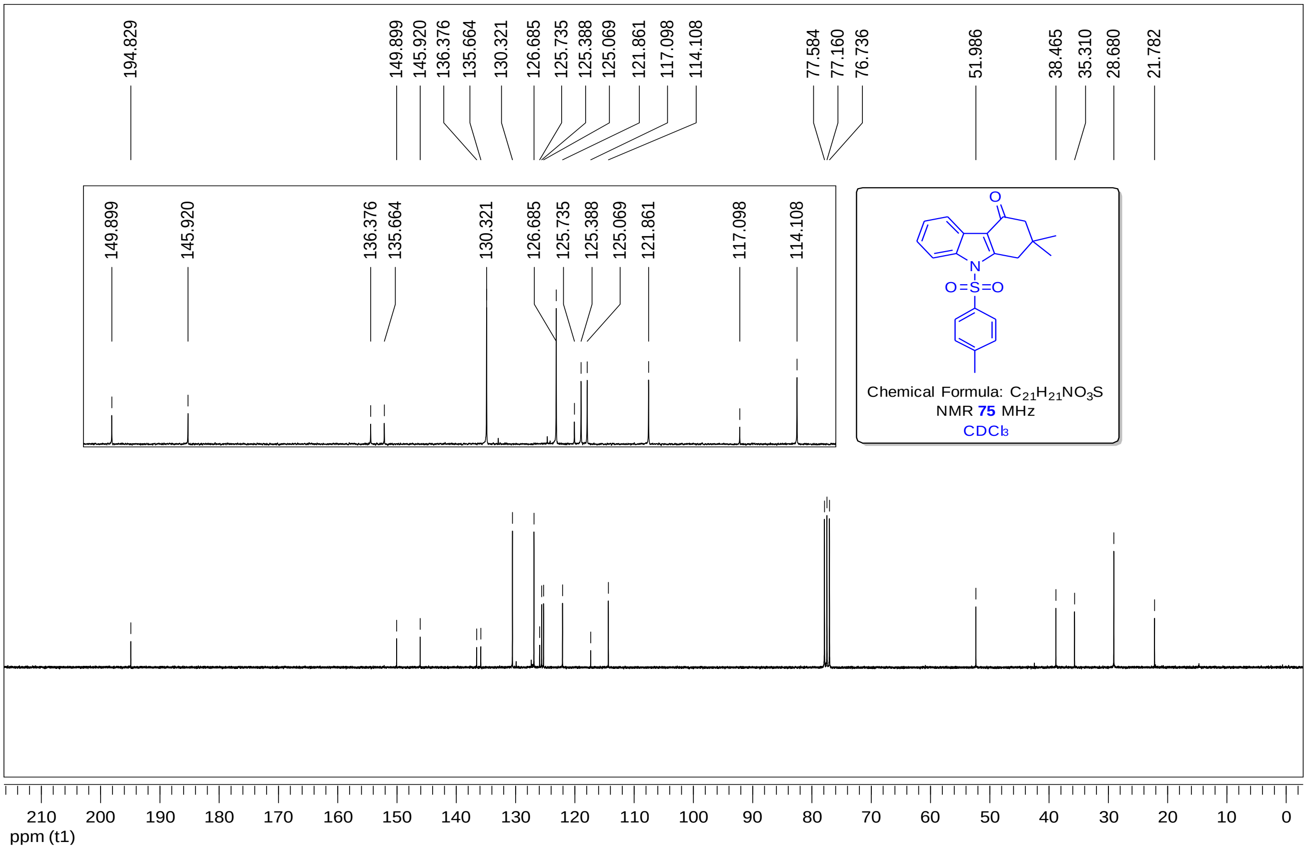

t-Butyl 1-oxo-1,2,3,4-tetrahydro-9*H*-carbazole-9-carboxylate (4h)

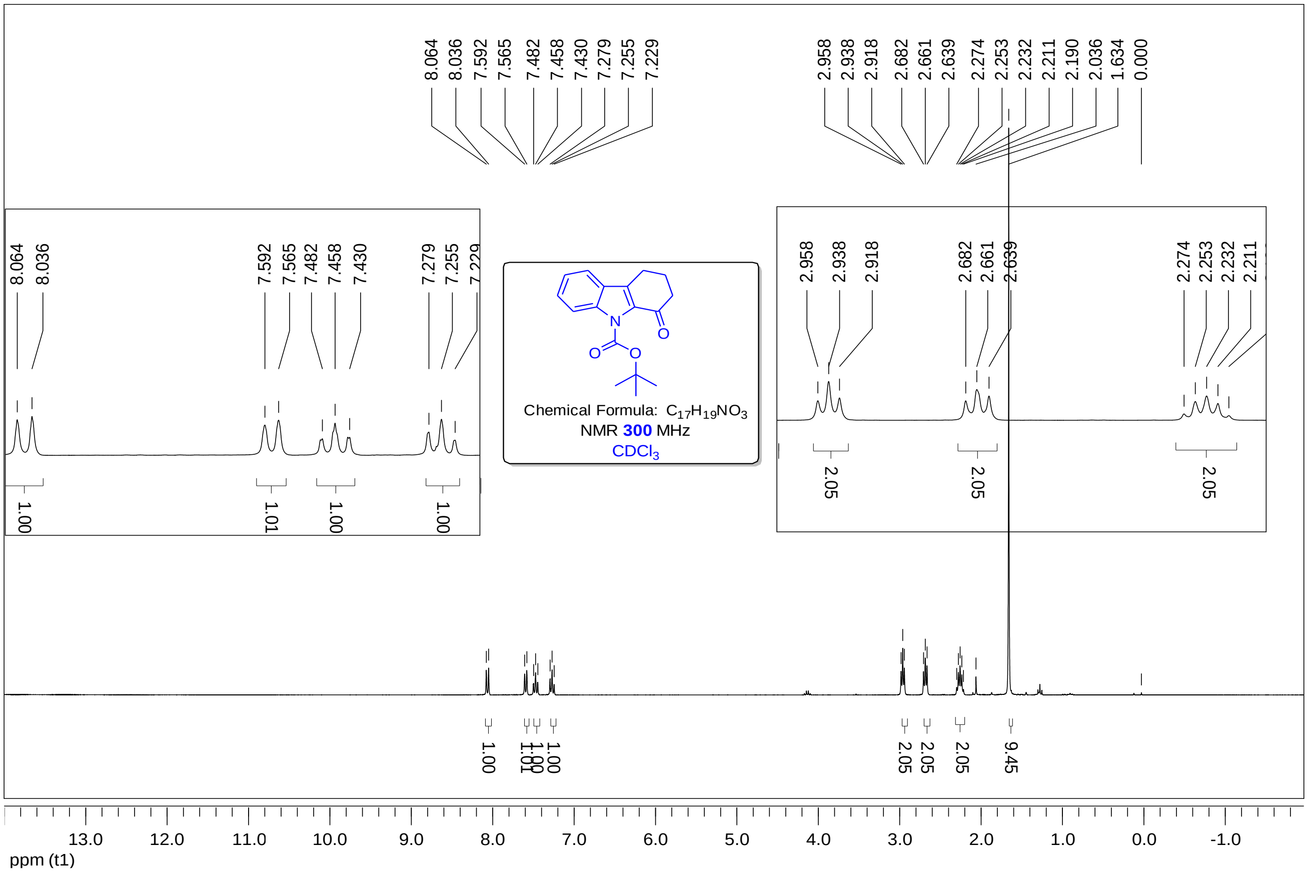

t-Butyl 1-oxo-1,2,3,4-tetrahydro-9*H*-carbazole-9-carboxylate (4h)

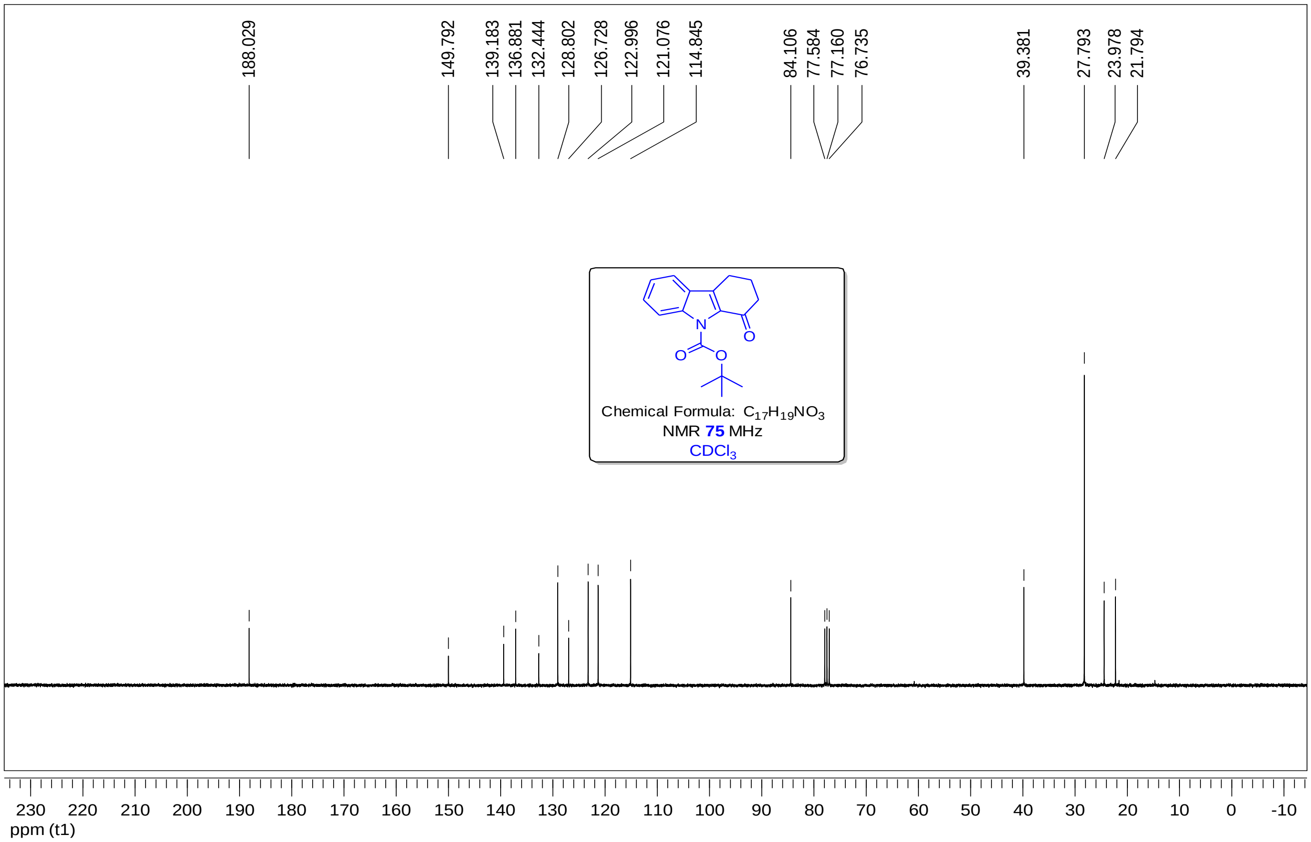

9-(Methylsulfonyl)-2,3,4,9-tetrahydro-1*H*-carbazol-1-one (4i)

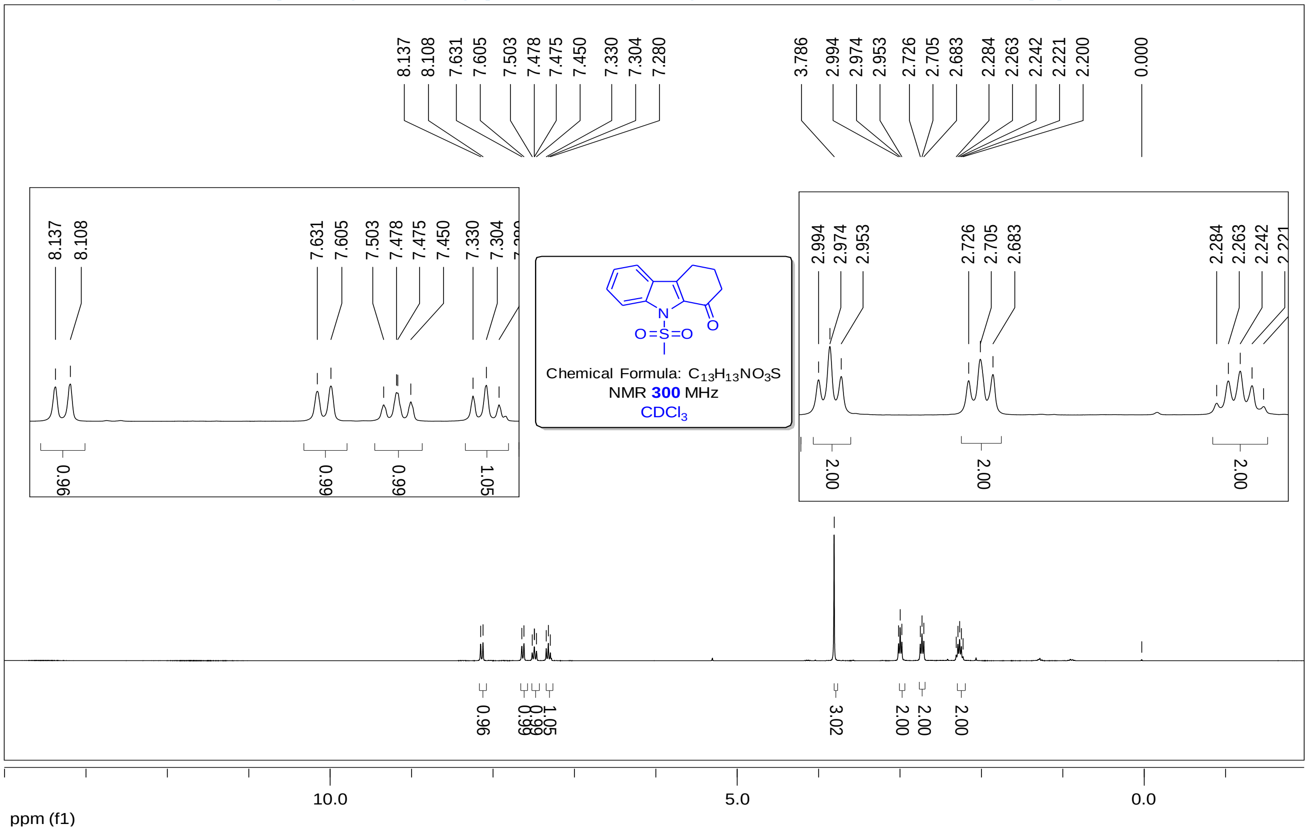

9-(Methylsulfonyl)-2,3,4,9-tetrahydro-1*H*-carbazol-1-one (4i)

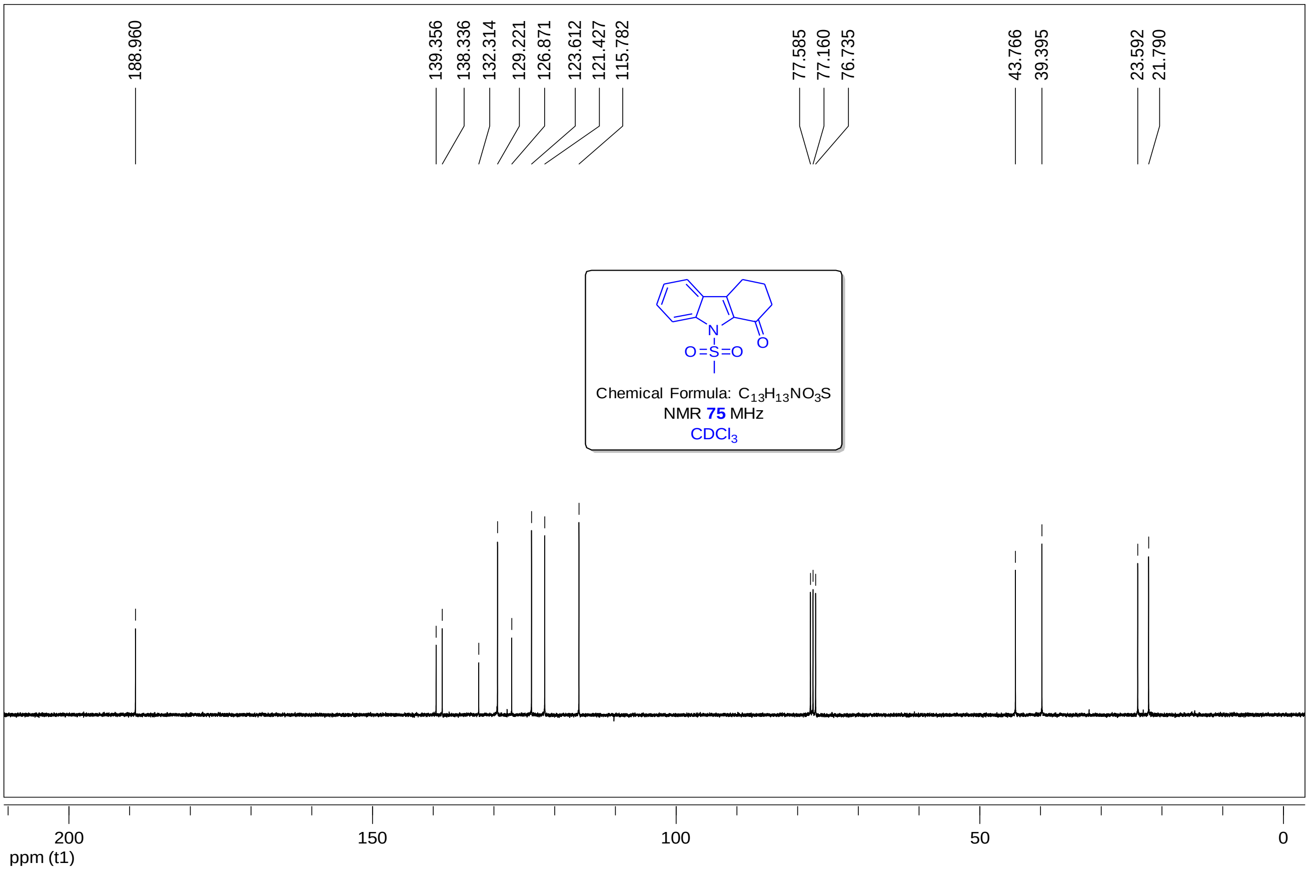

1-(Methylsulfonyl)-1,5,6,7-tetrahydro-4*H*-indol-4-one (4o)

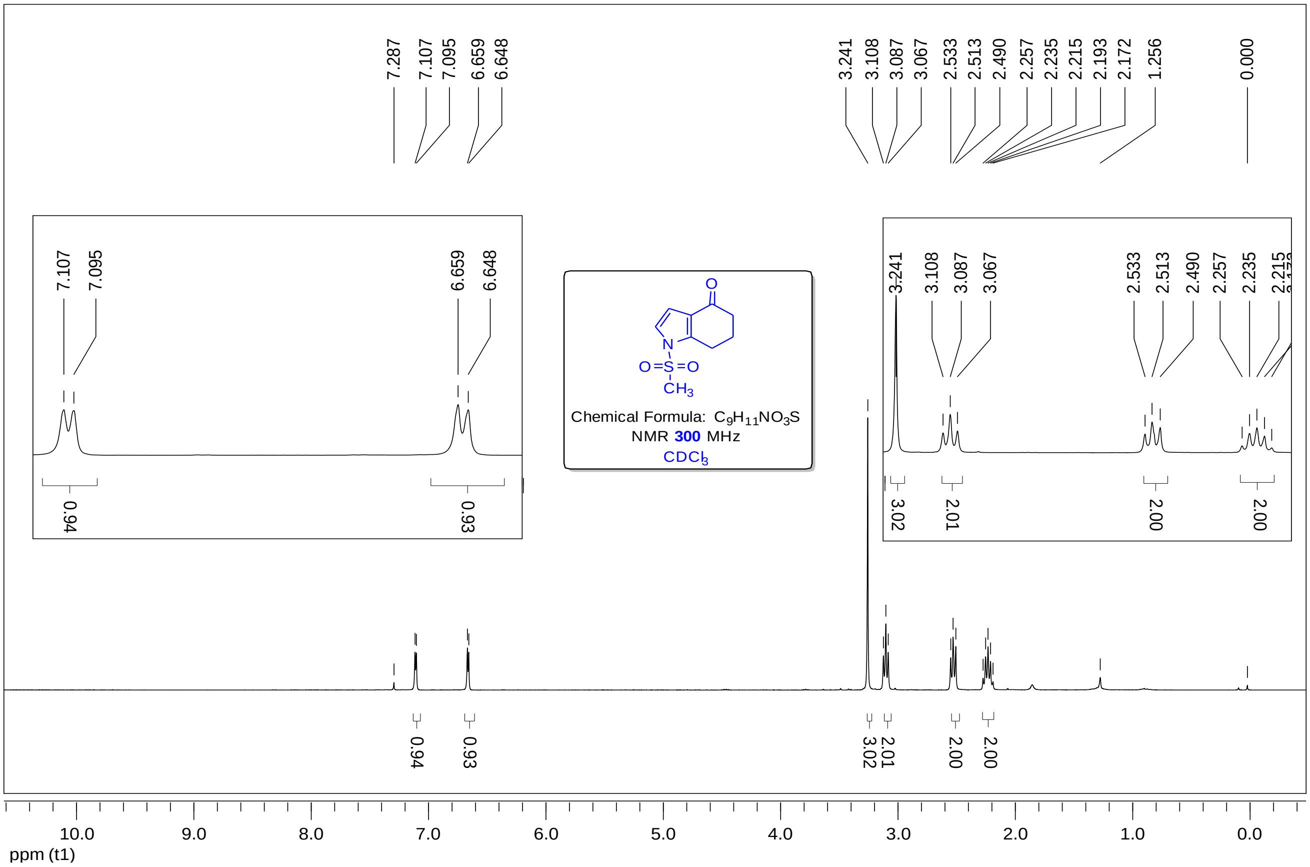

1-(Methylsulfonyl)-1,5,6,7-tetrahydro-4*H*-indol-4-one (4o)

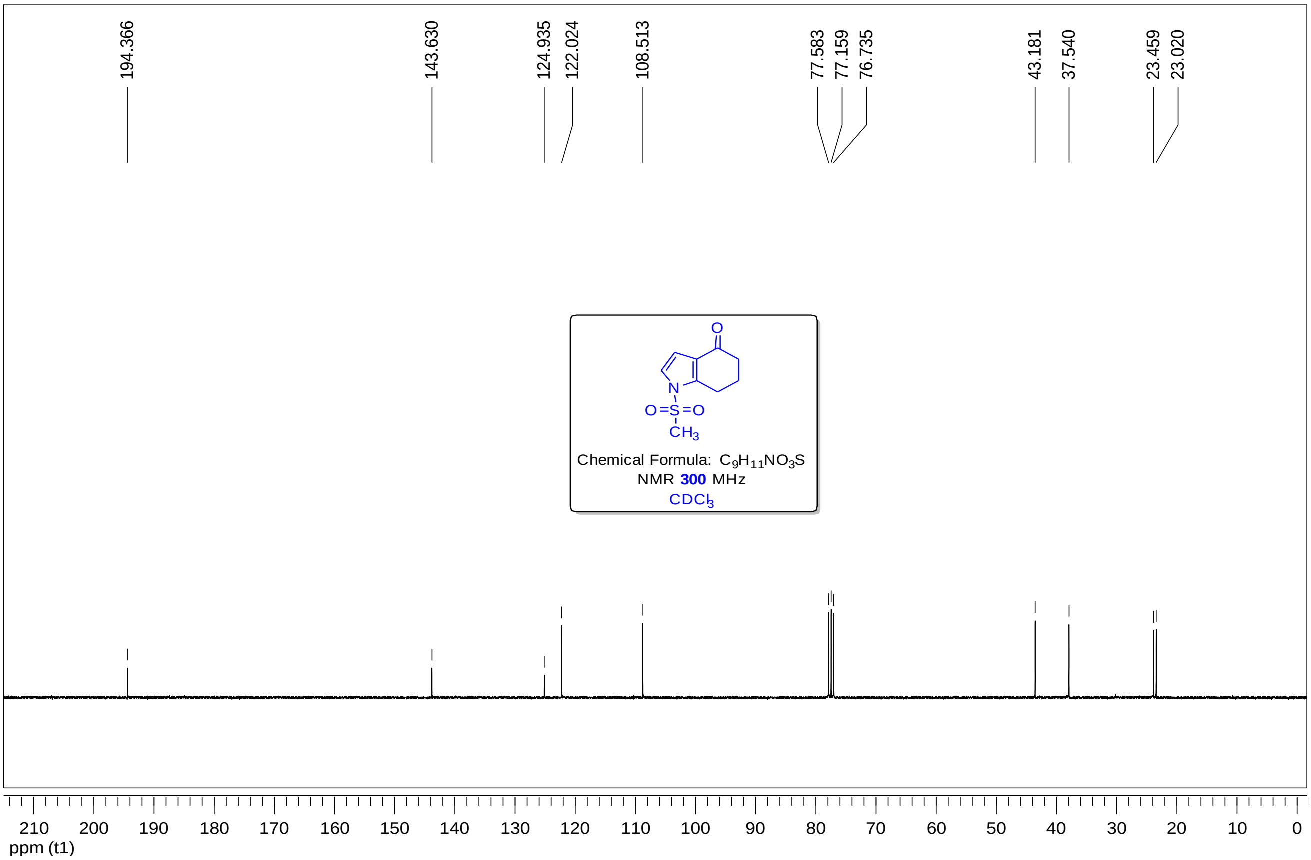

t-Butyl 4-oxo-4,5,6,7-tetrahydro-1*H*-indole-1-carboxylate (4p)

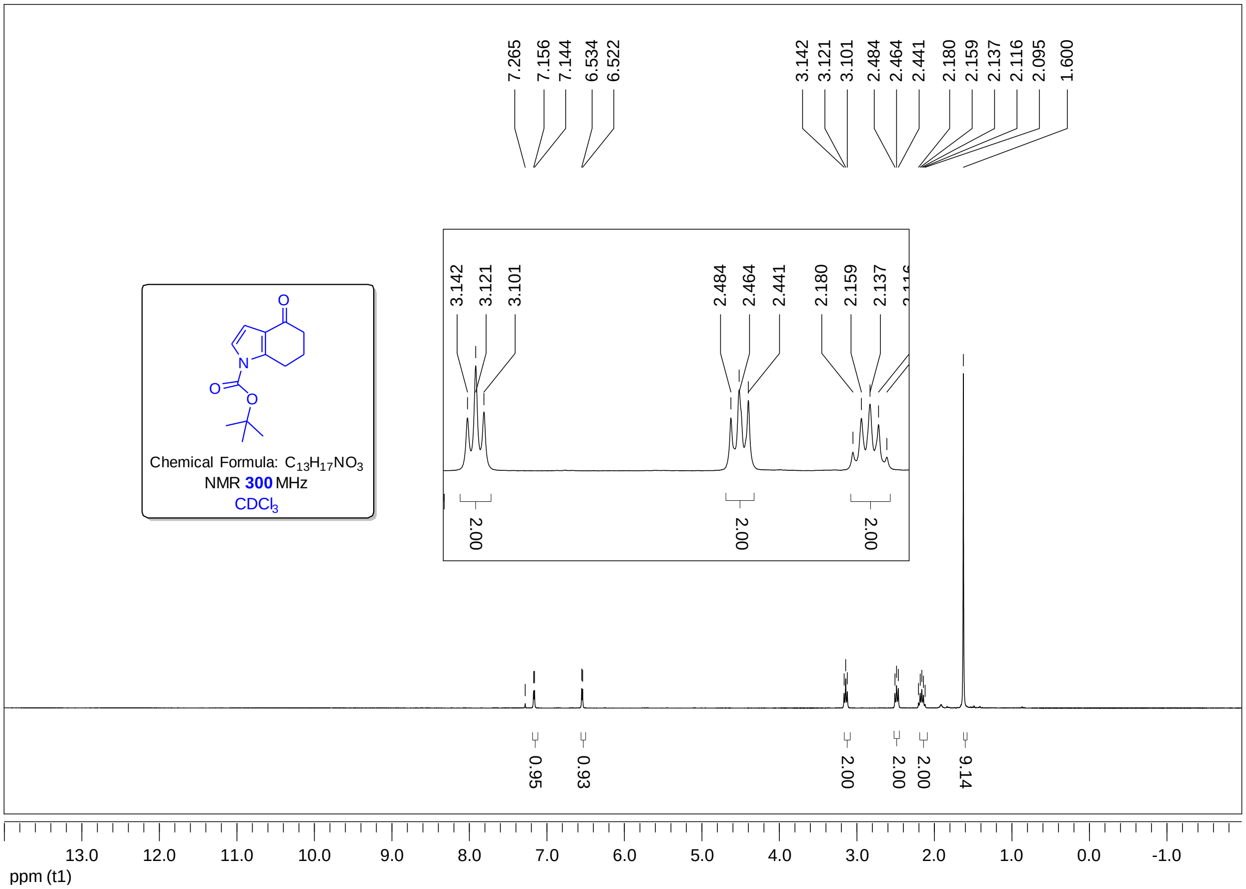

t-Butyl 4-oxo-4,5,6,7-tetrahydro-1*H*-indole-1-carboxylate (4p)

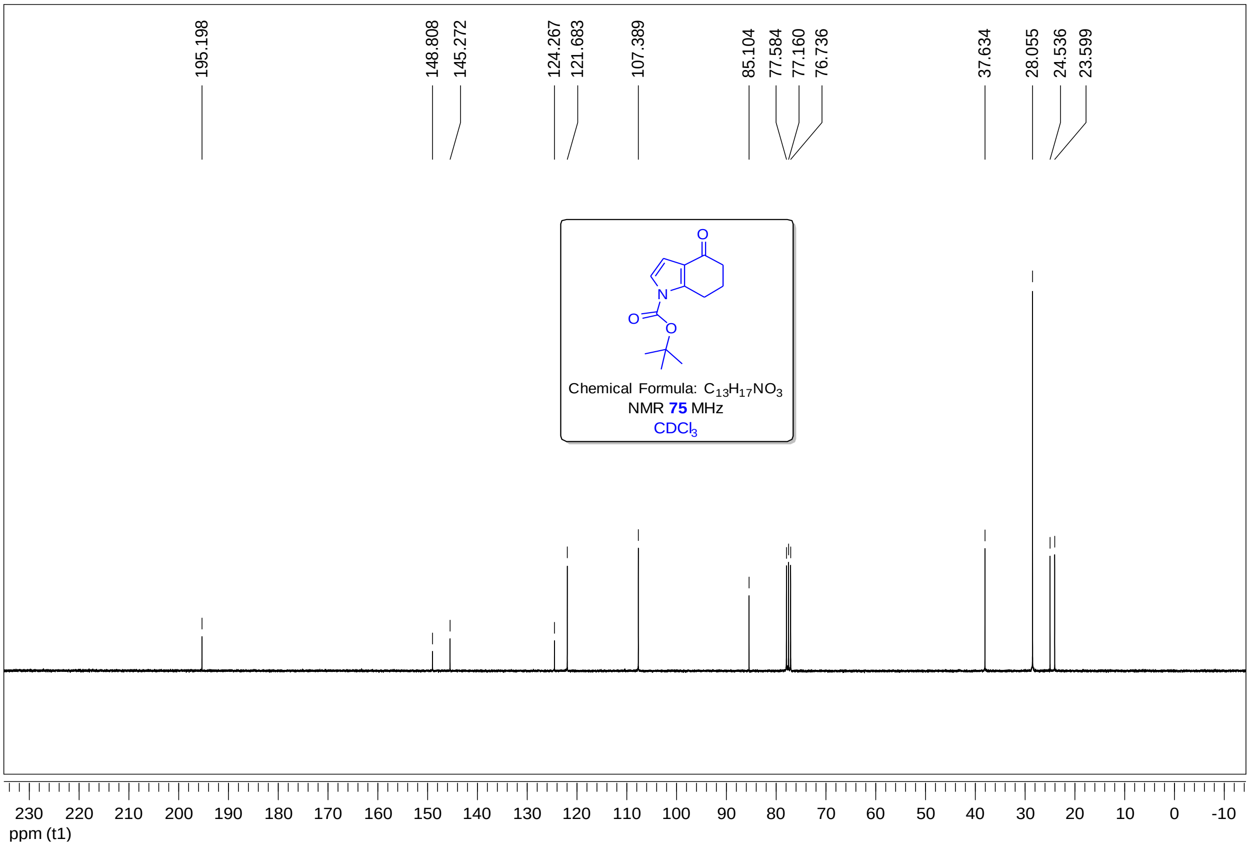

1-(*t*-Butyldimethylsilyl)-1,5,6,7-tetrahydro-4*H*-indol-4-one (4q)

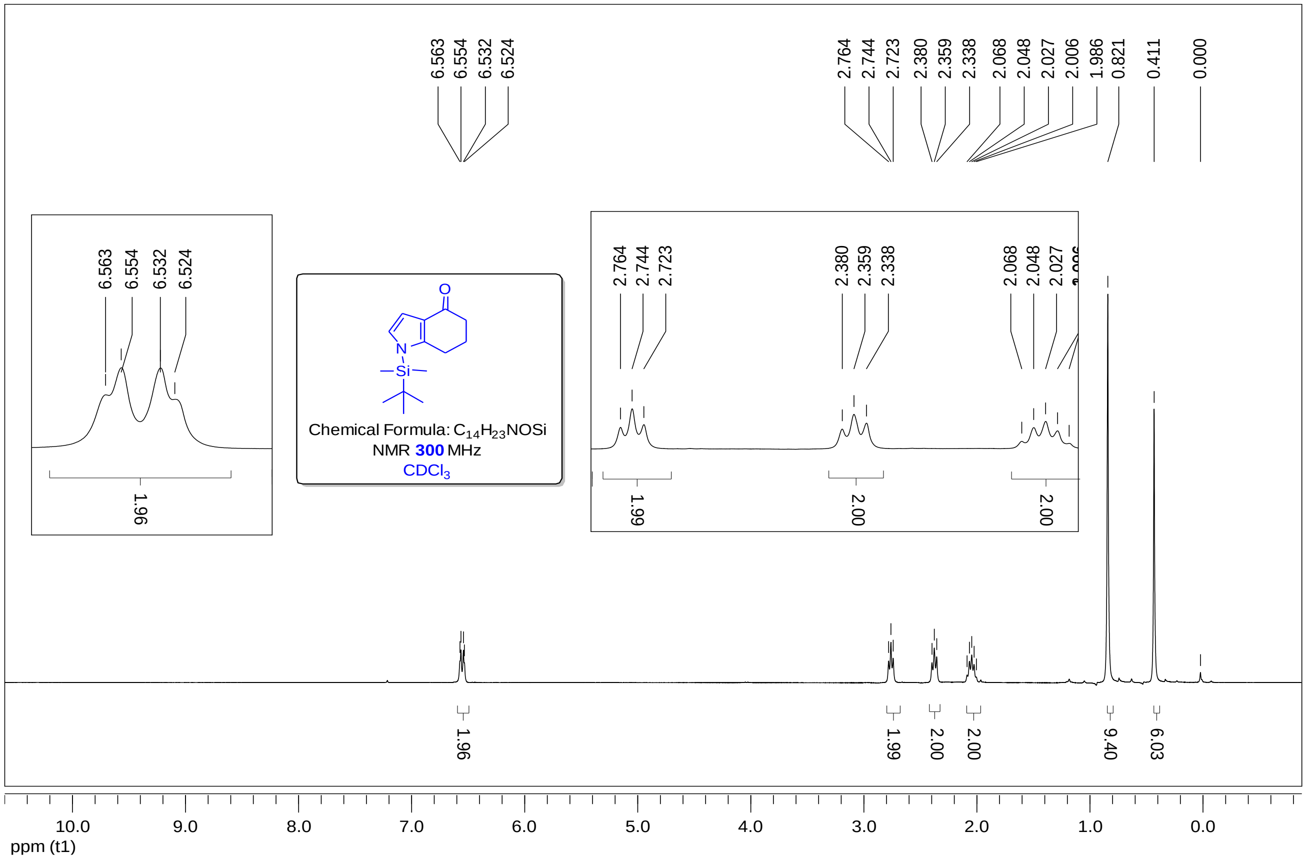

1-(*t*-Butyldimethylsilyl)-1,5,6,7-tetrahydro-4*H*-indol-4-one (4q)

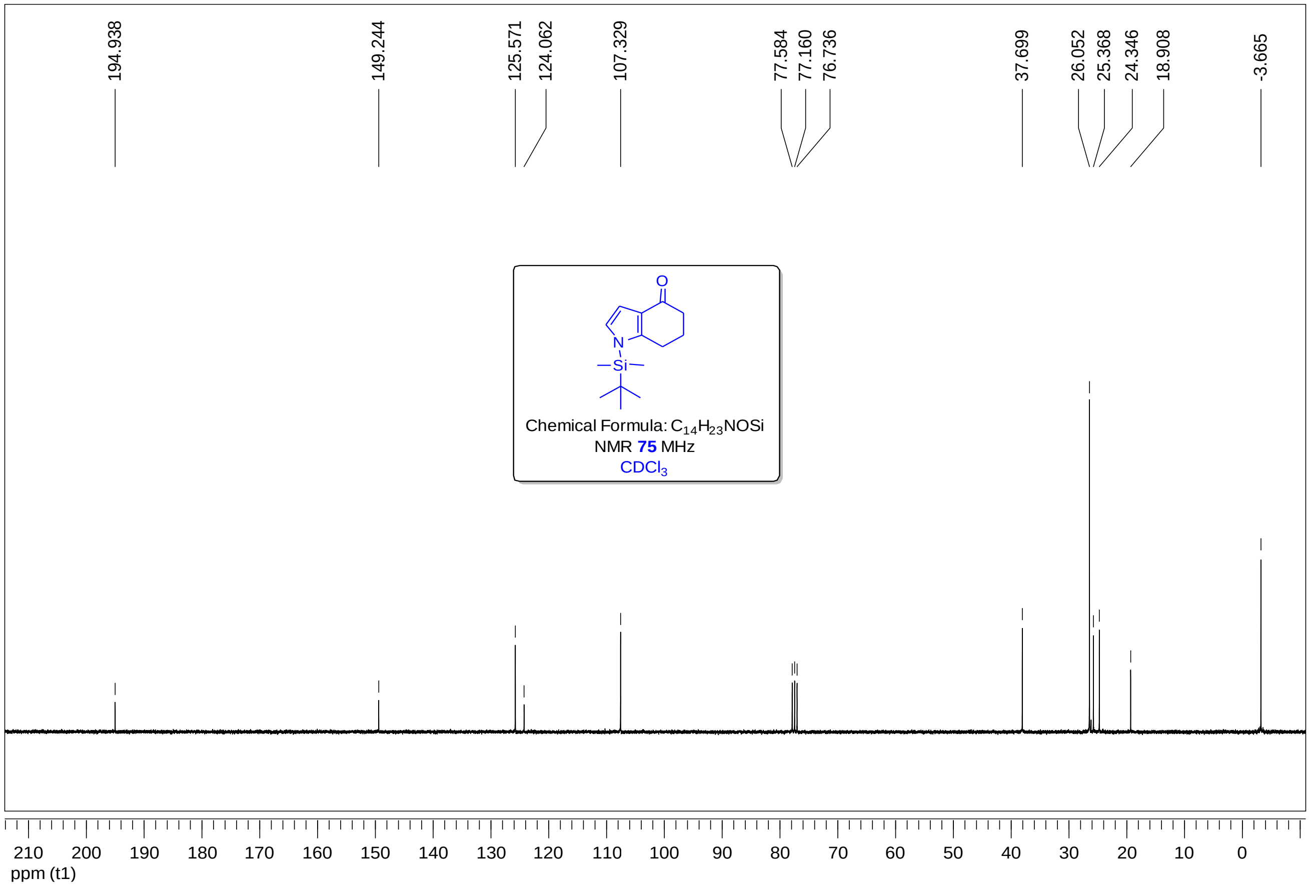

1-Benzyl-1,5,6,7-tetrahydro-4*H*-indol-4-one (4r)

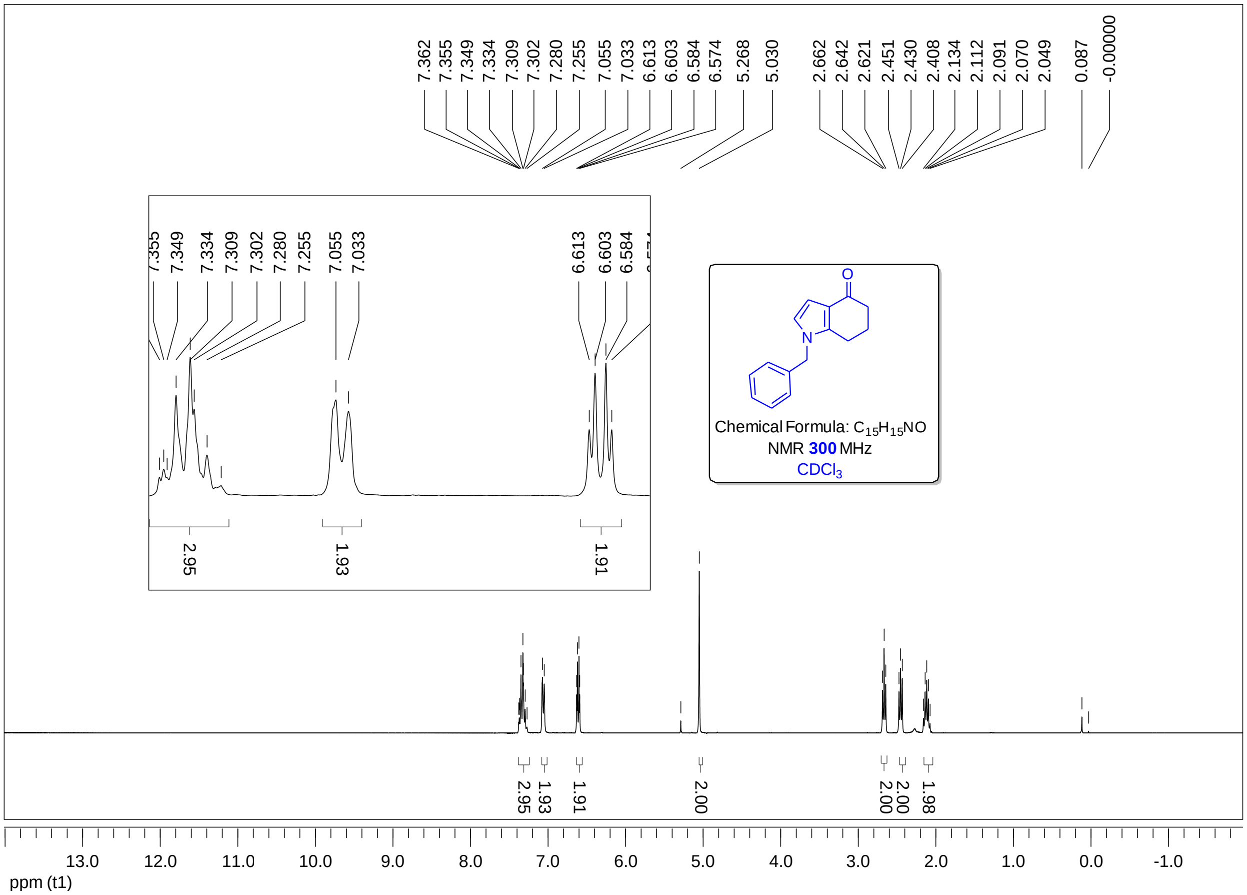

1-Benzyl-1,5,6,7-tetrahydro-4*H*-indol-4-one (4r)

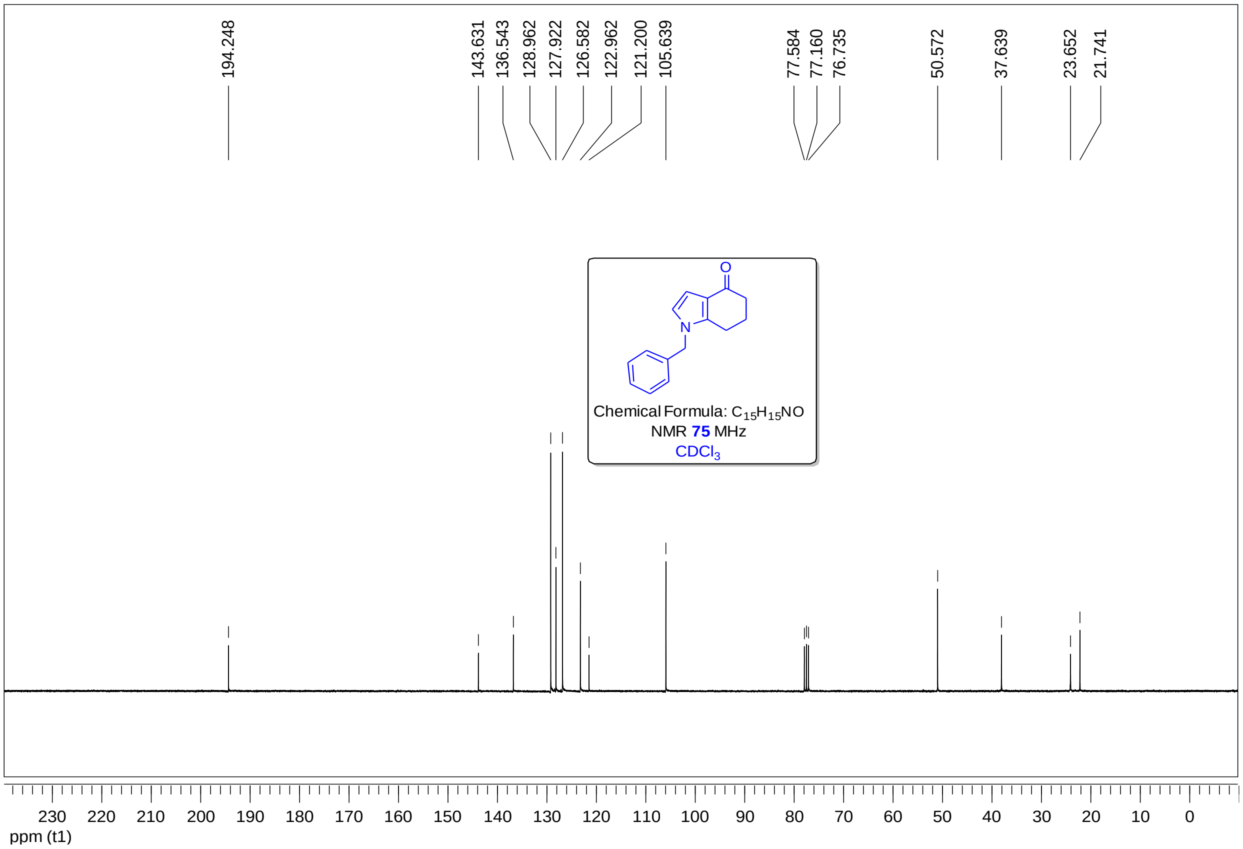

7,8-Tetrahydro-*5H*-cyclohepta[*b*]thiophen-5-one (4aa)

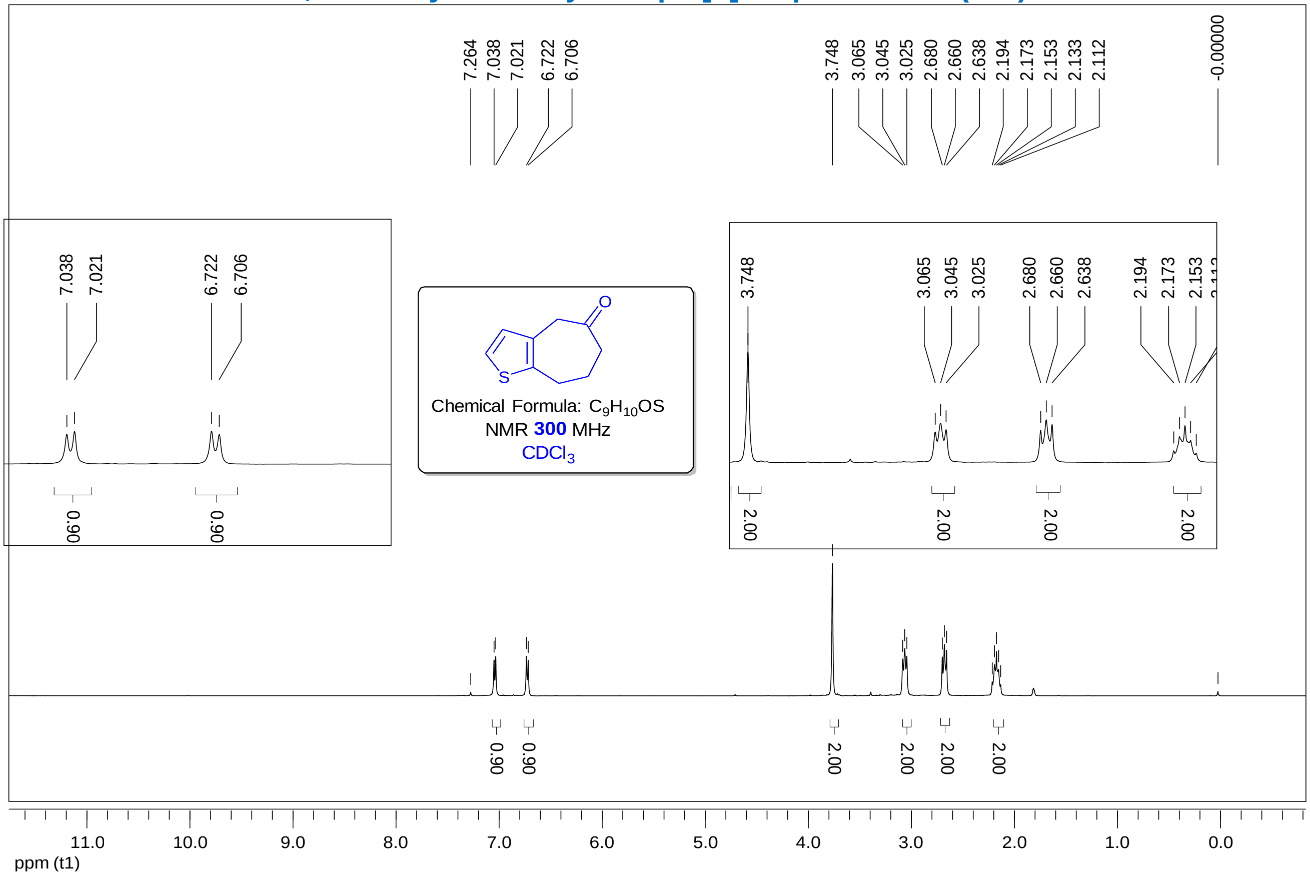

4,6,7,8-Tetrahydro-*5H*-cyclohepta[*b*]thiophen-5-one (4aa)

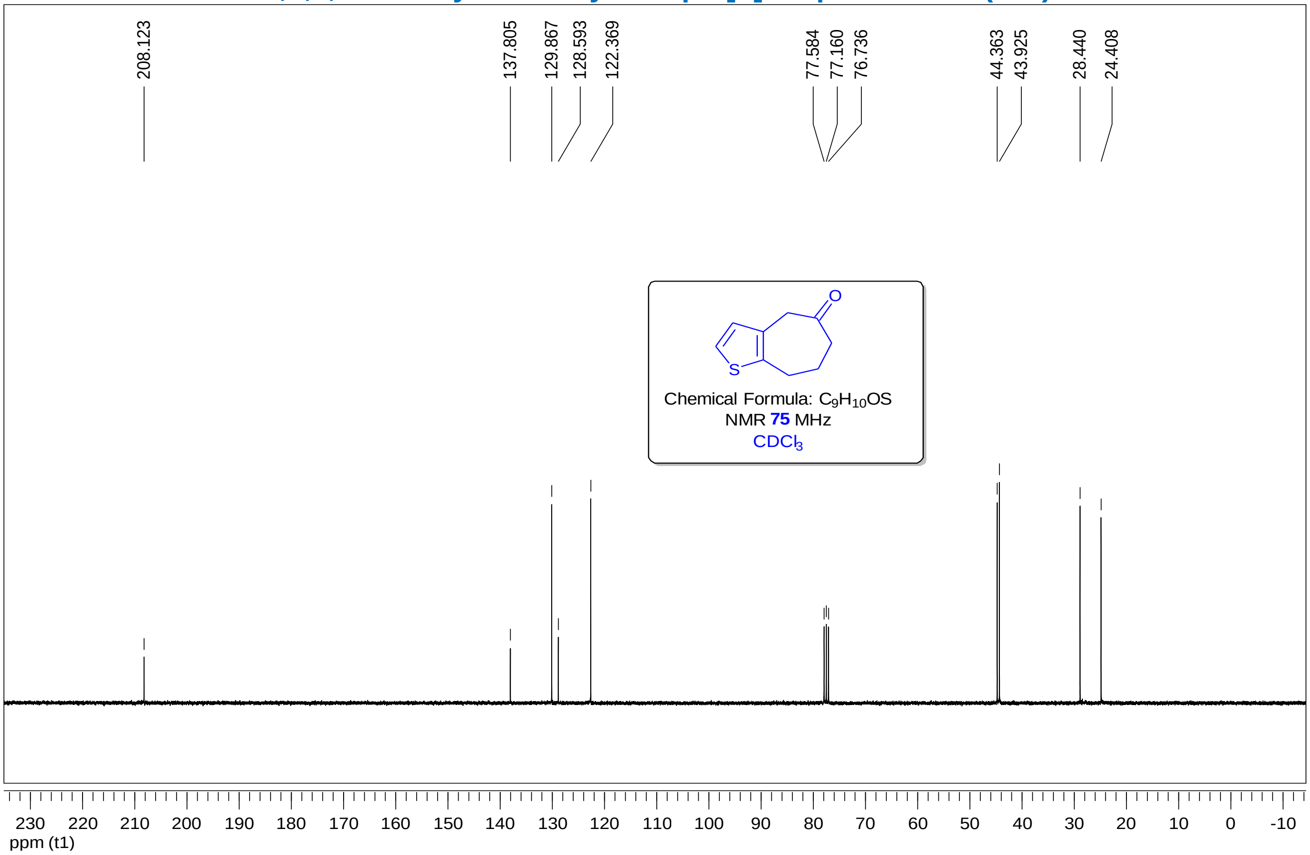

4-Methylene-4,5,6,7-tetrahydrobenzo[*b*]thiophene (**5a**)

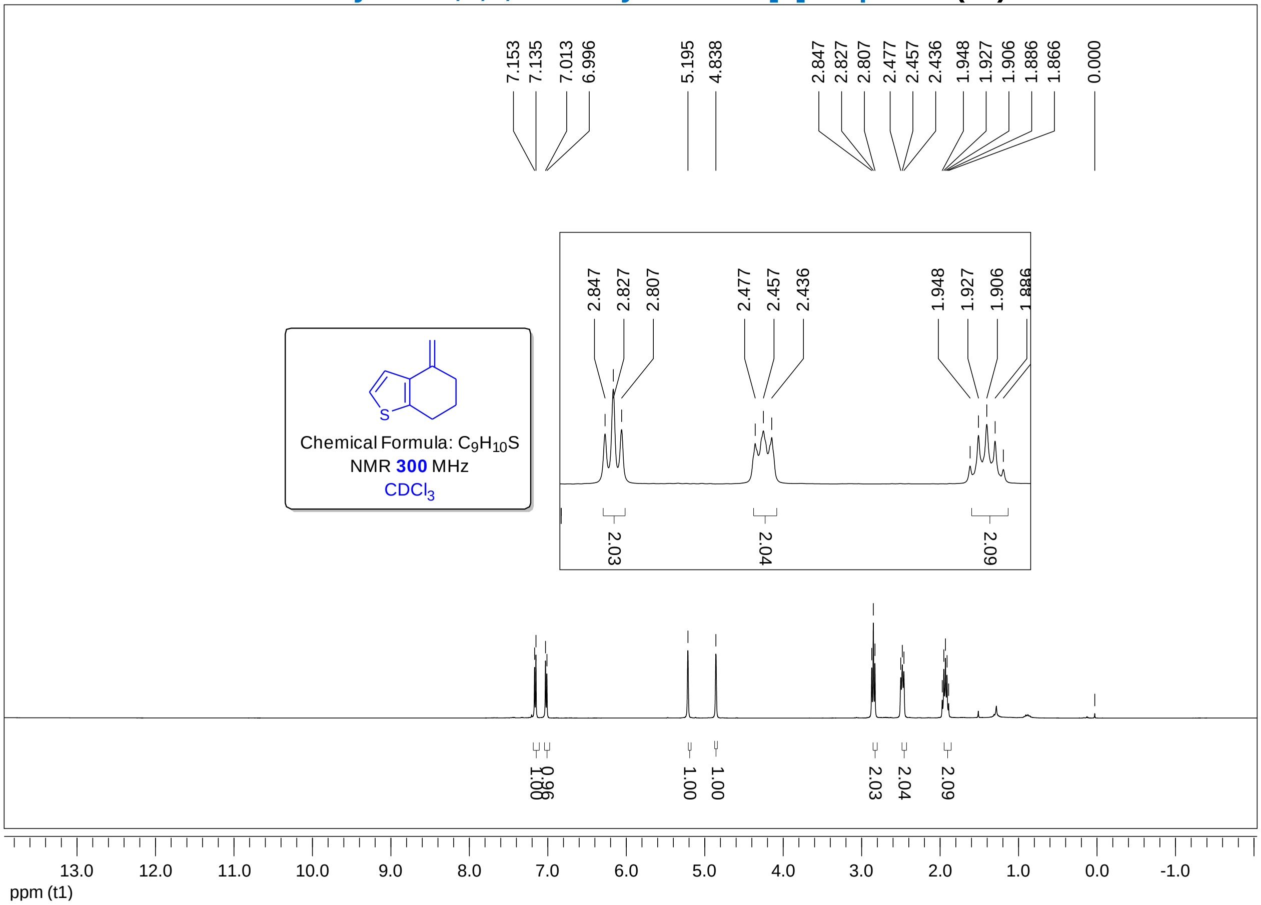

4-Methylene-4,5,6,7-tetrahydrobenzo[*b*]thiophene (**5a**)

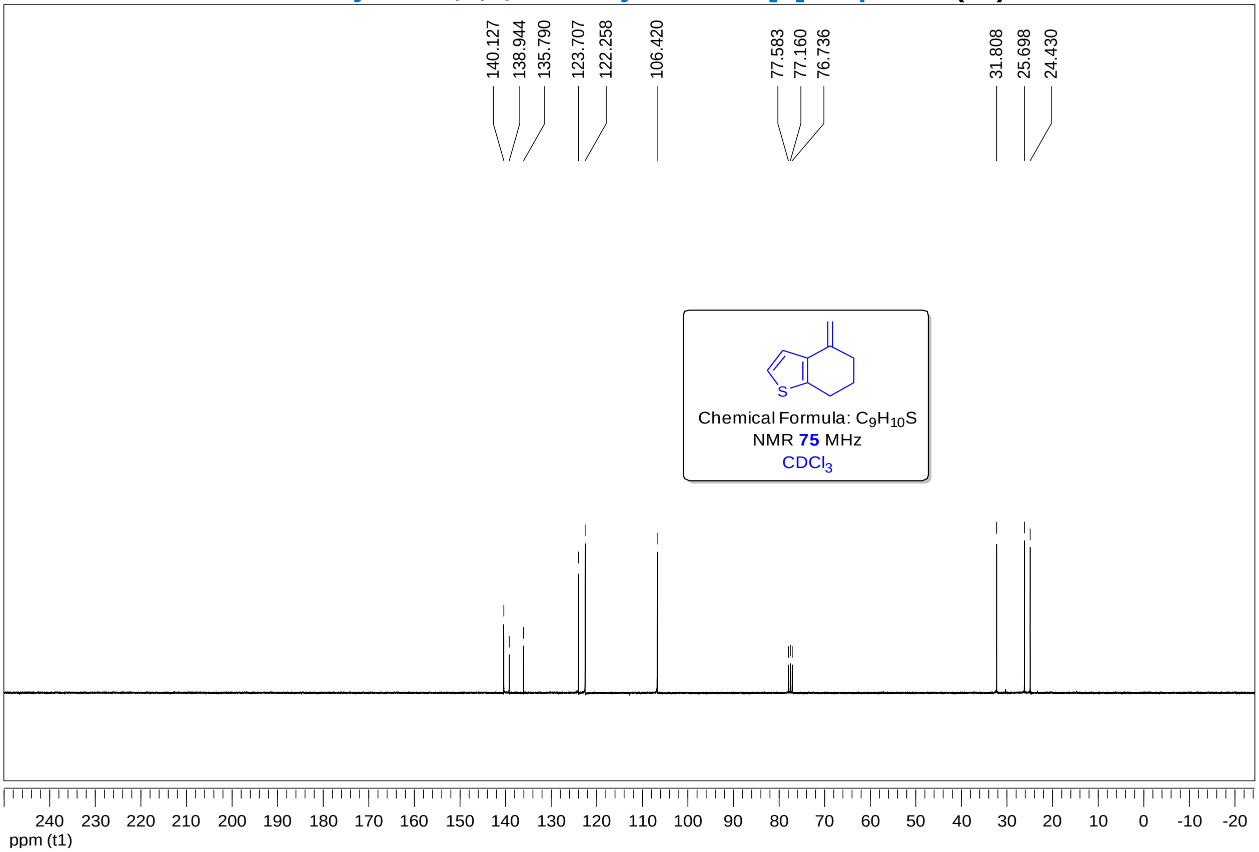

5-Methylene-5,6,7,8-tetrahydro-4*H*-cyclohepta[*b*]thiophene (5aa)

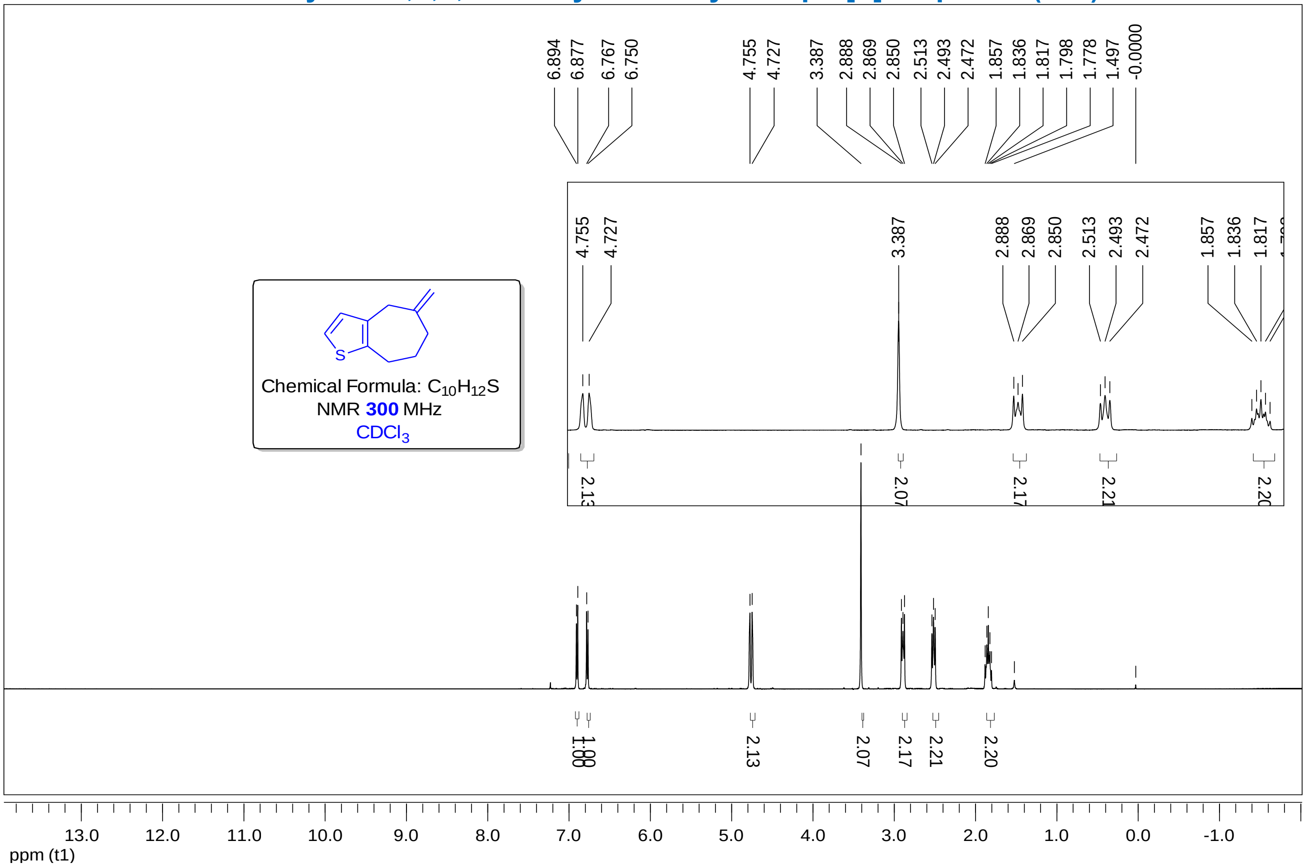

5-Methylene-5,6,7,8-tetrahydro-4*H*-cyclohepta[*b*]thiophene (5aa)

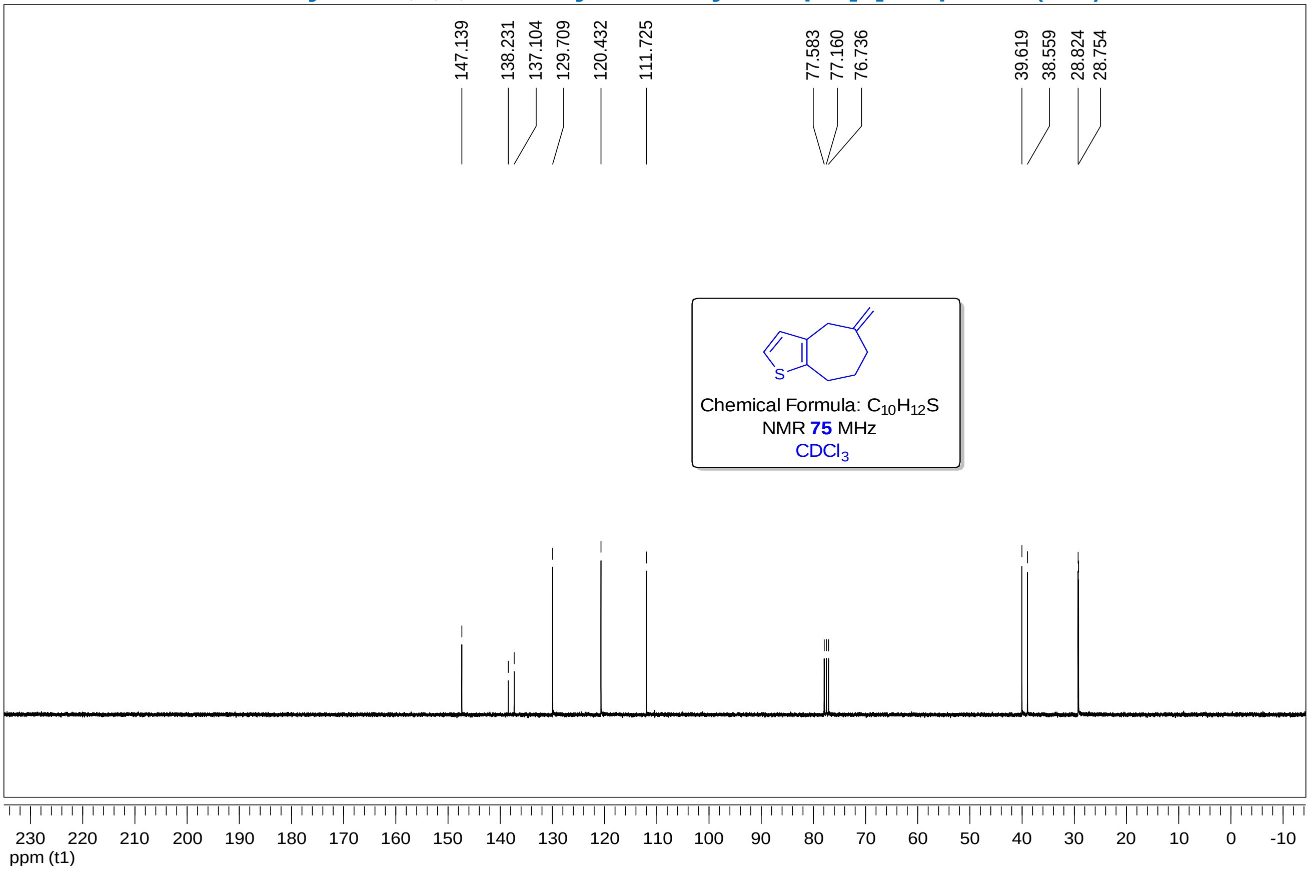

4-Methylene-4,5,6,7-tetrahydrobenzofuran (5b)

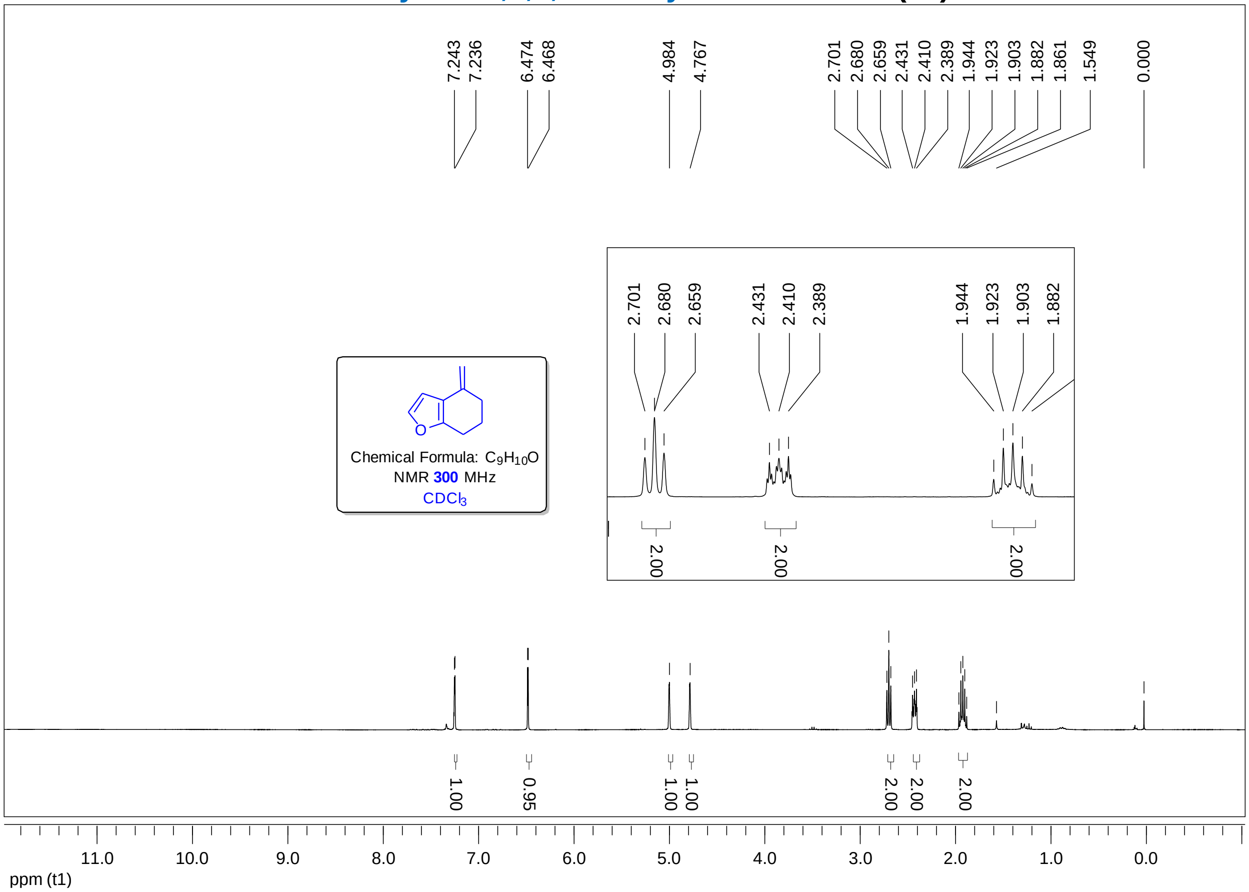

4-Methylene-4,5,6,7-tetrahydrobenzofuran (5b)

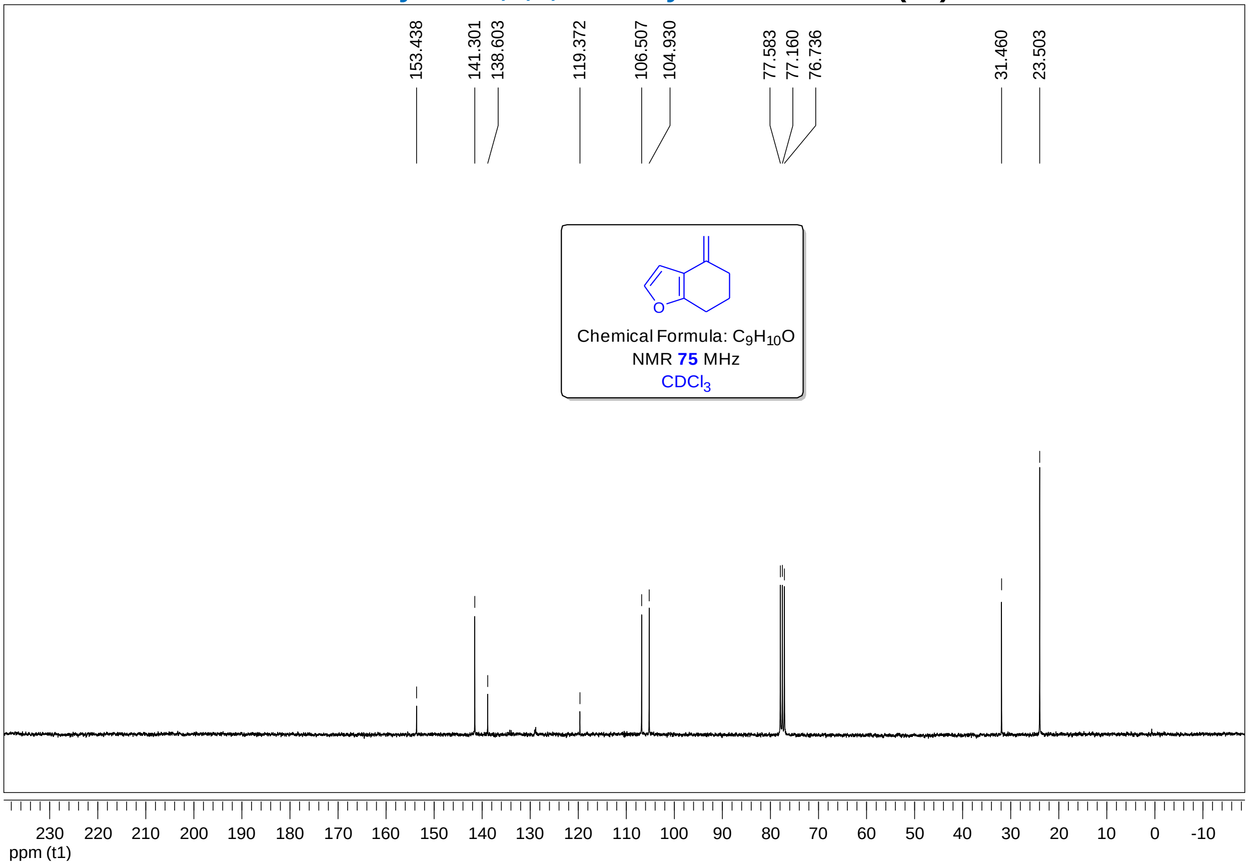

6,6-Dimethyl-4-methylene-4,5,6,7-tetrahydrobenzofuran (5c)

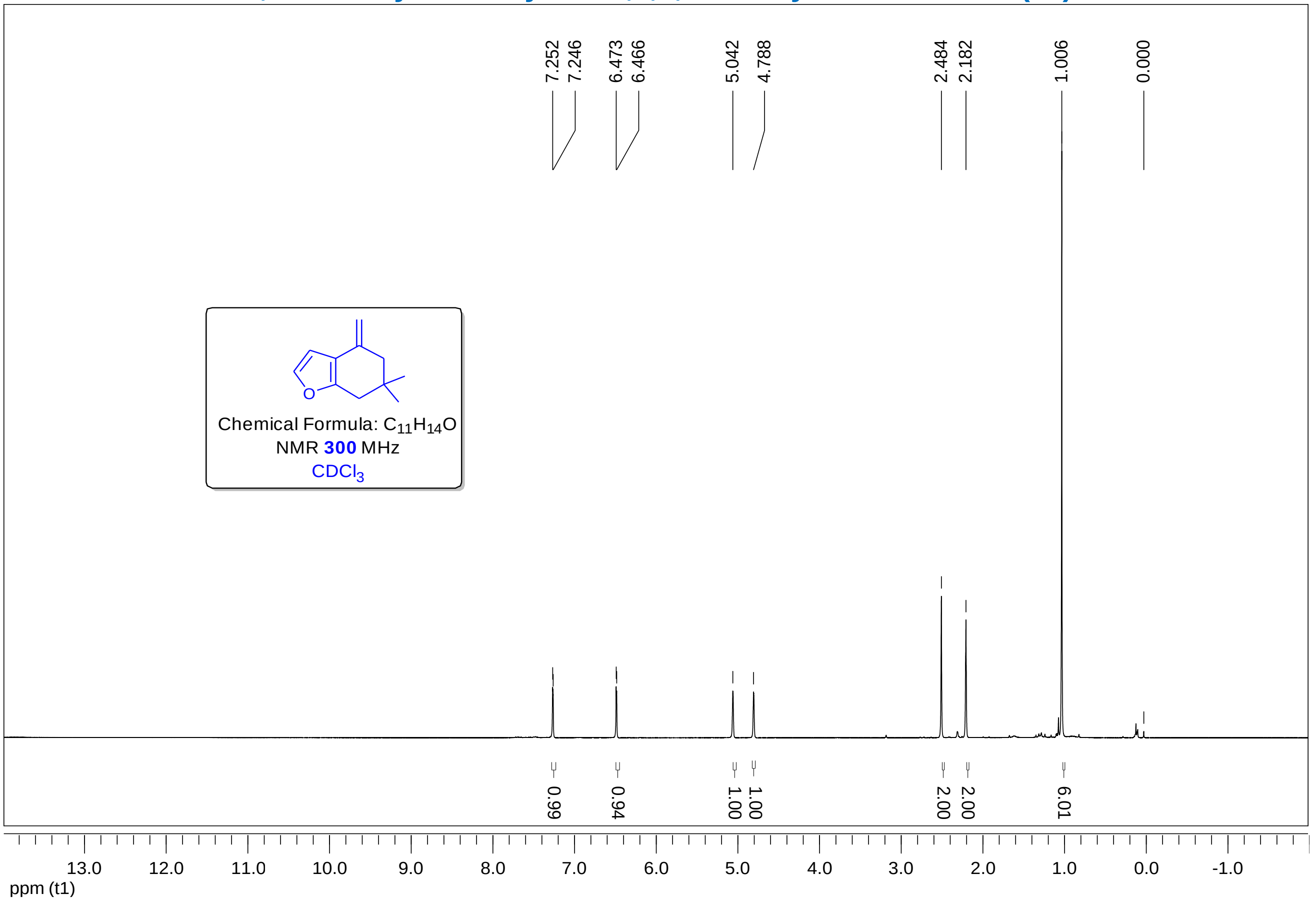

6,6-Dimethyl-4-methylene-4,5,6,7-tetrahydrobenzofuran (5c)

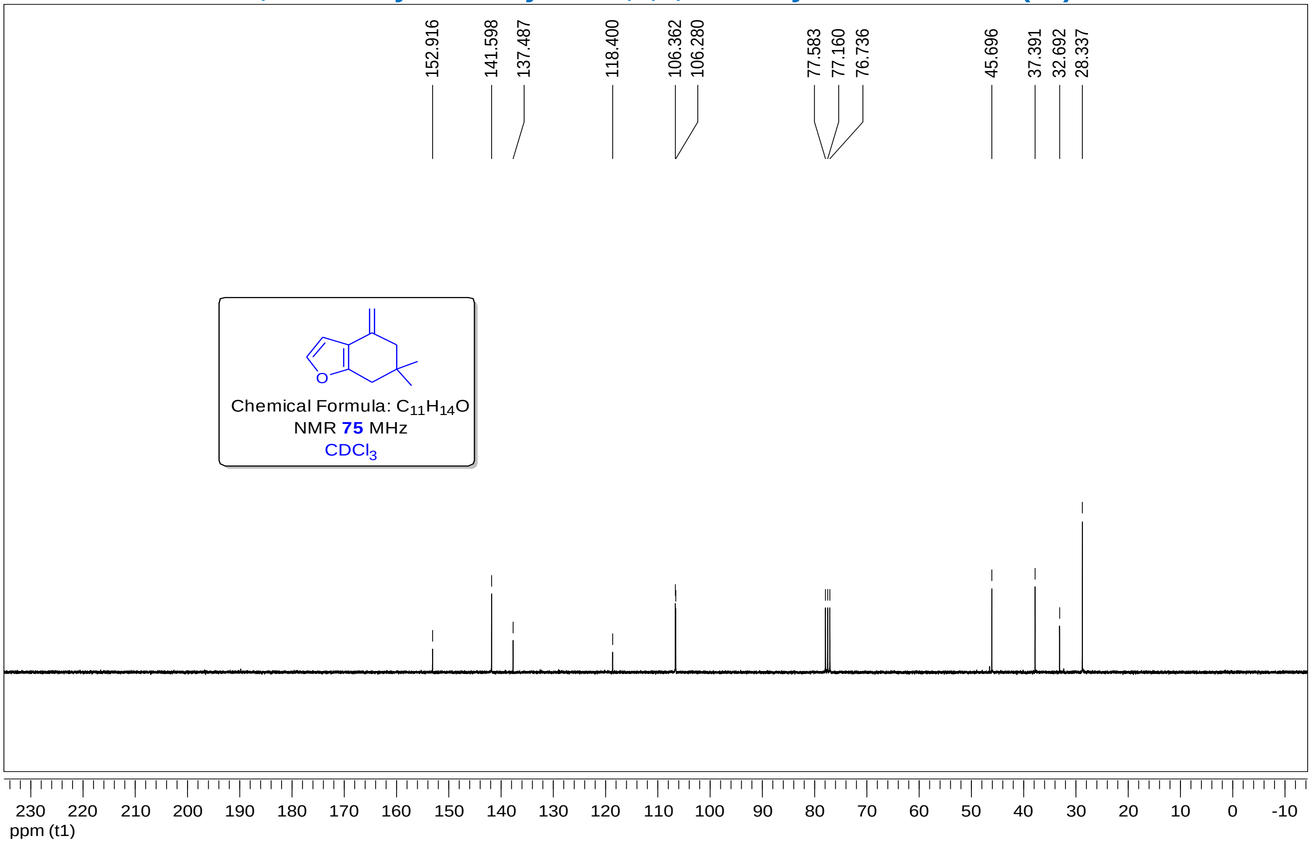

4-Methylene-9-tosyl-2,3,4,9-tetrahydro-1*H*-carbazole (5f)

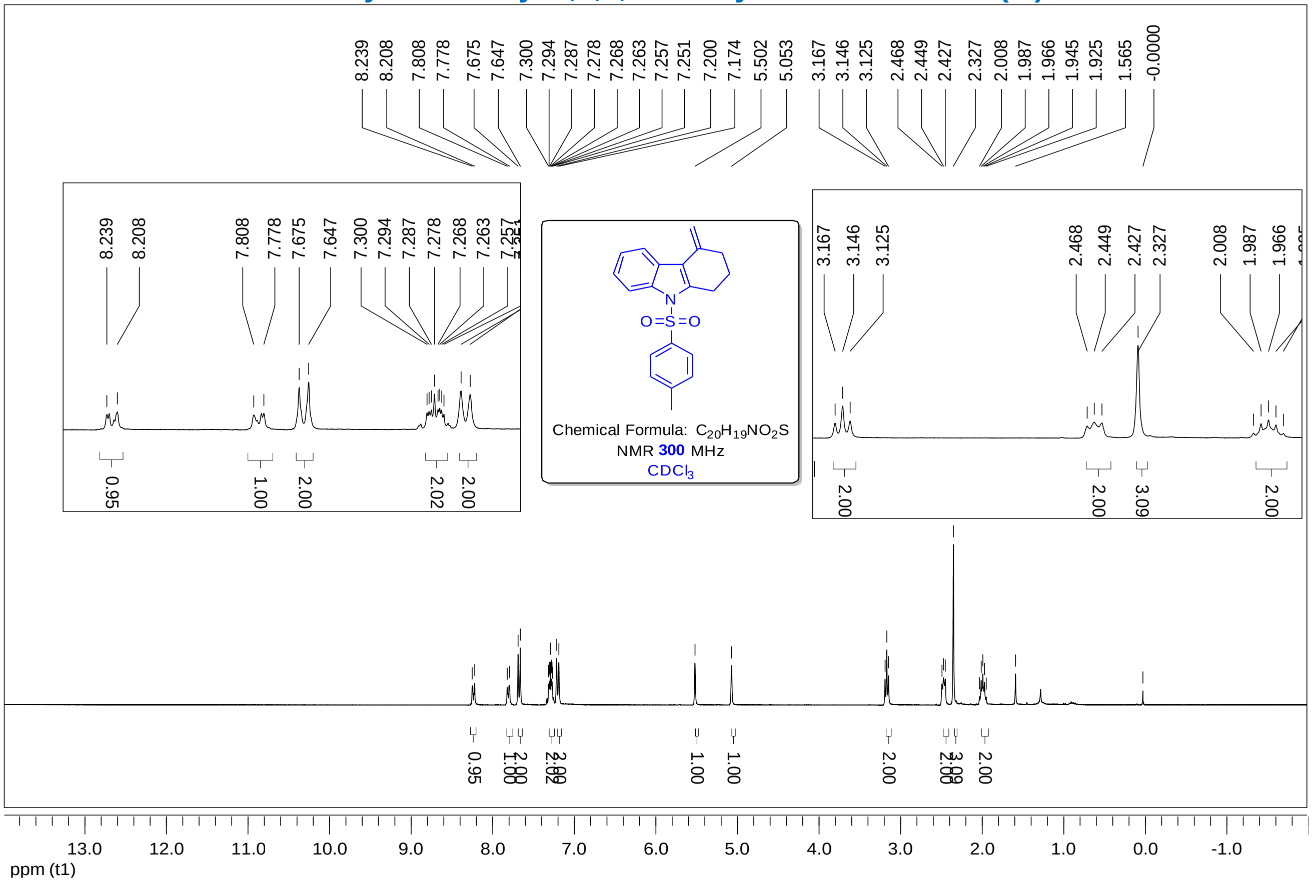

4-Methylene-9-tosyl-2,3,4,9-tetrahydro-1*H*-carbazole (5f)

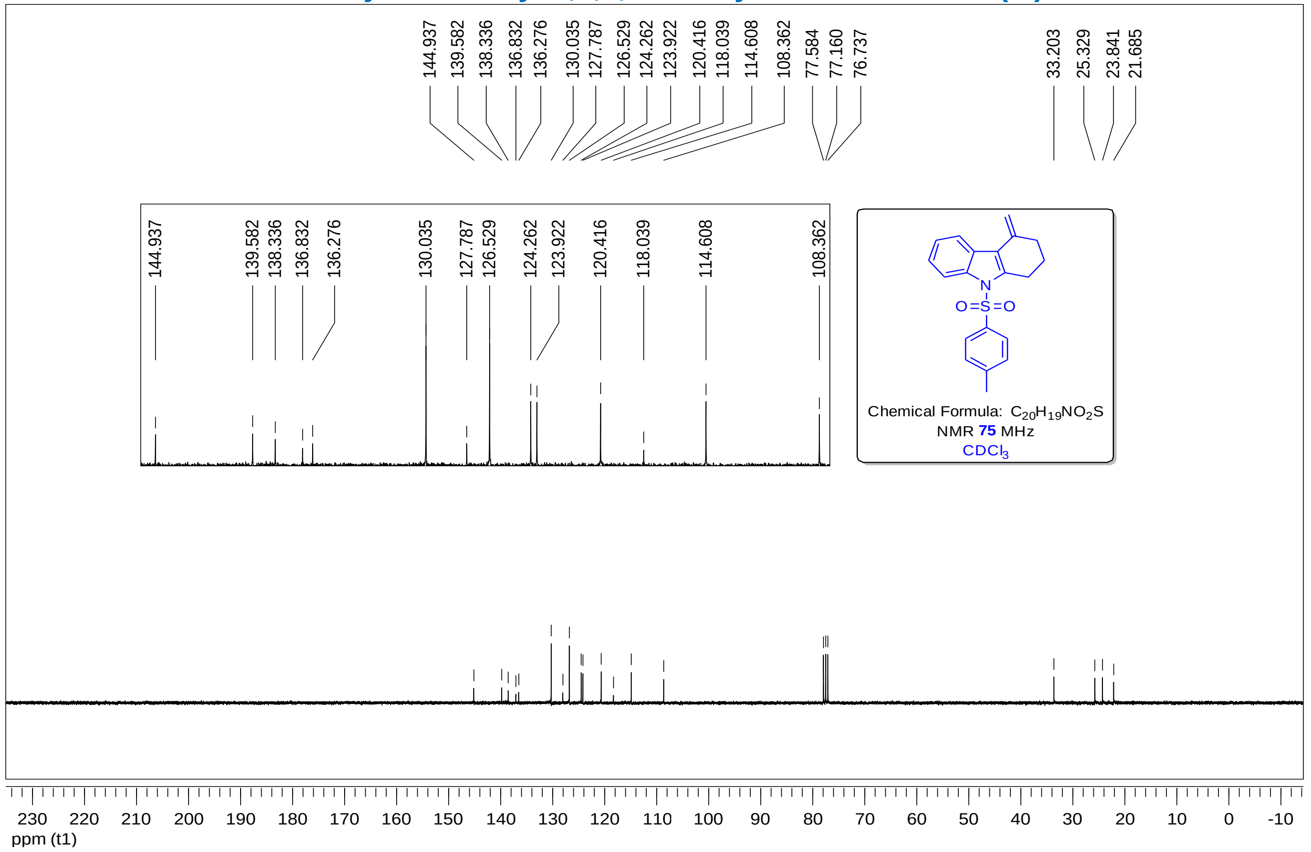

2,2-Dimethyl-4-methylene-9-tosyl-2,3,4,9-tetrahydro-1*H*-carbazole (5g)

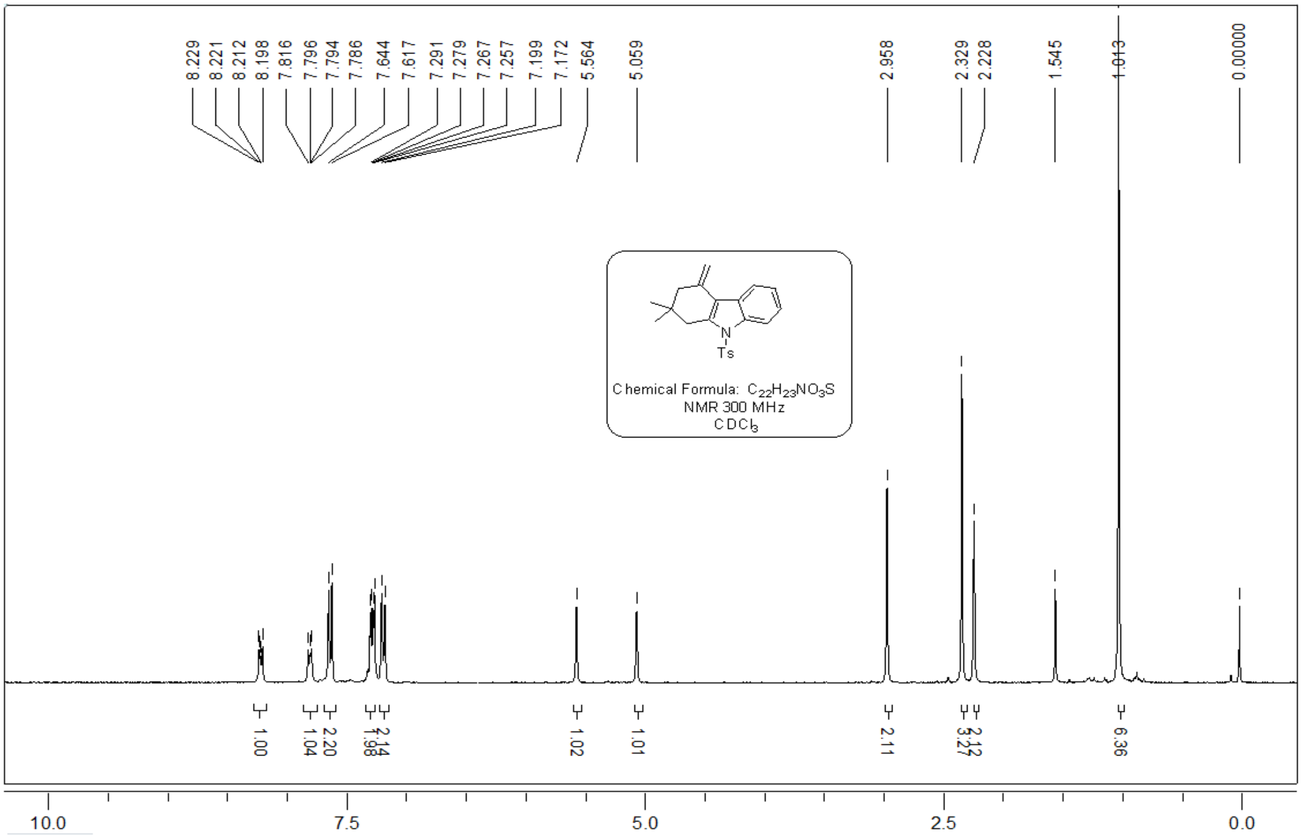

2,2-Dimethyl-4-methylene-9-tosyl-2,3,4,9-tetrahydro-1*H*-carbazole (5g)

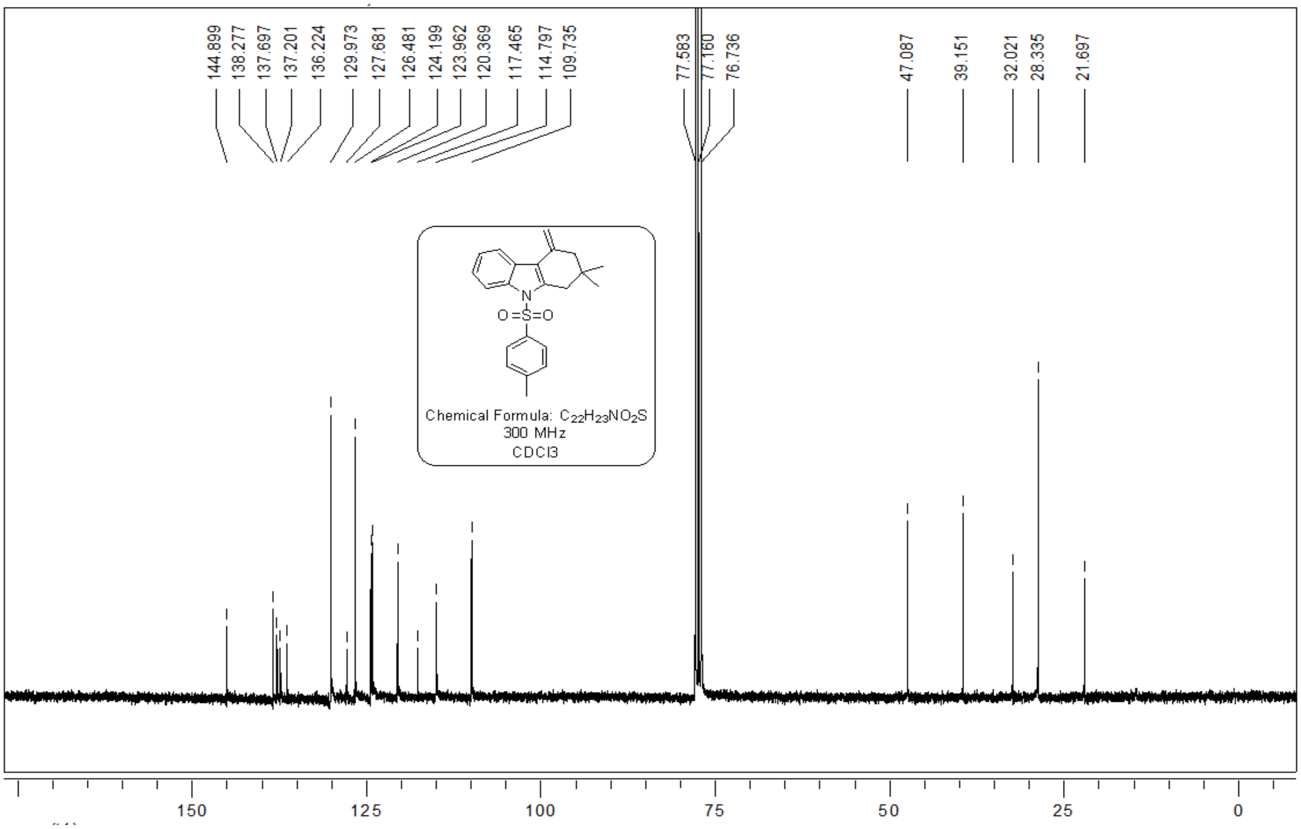

t-Butyl 1-methylene-1,2,3,4-tetrahydro-9*H*-carbazole-9-carboxylate (5h)

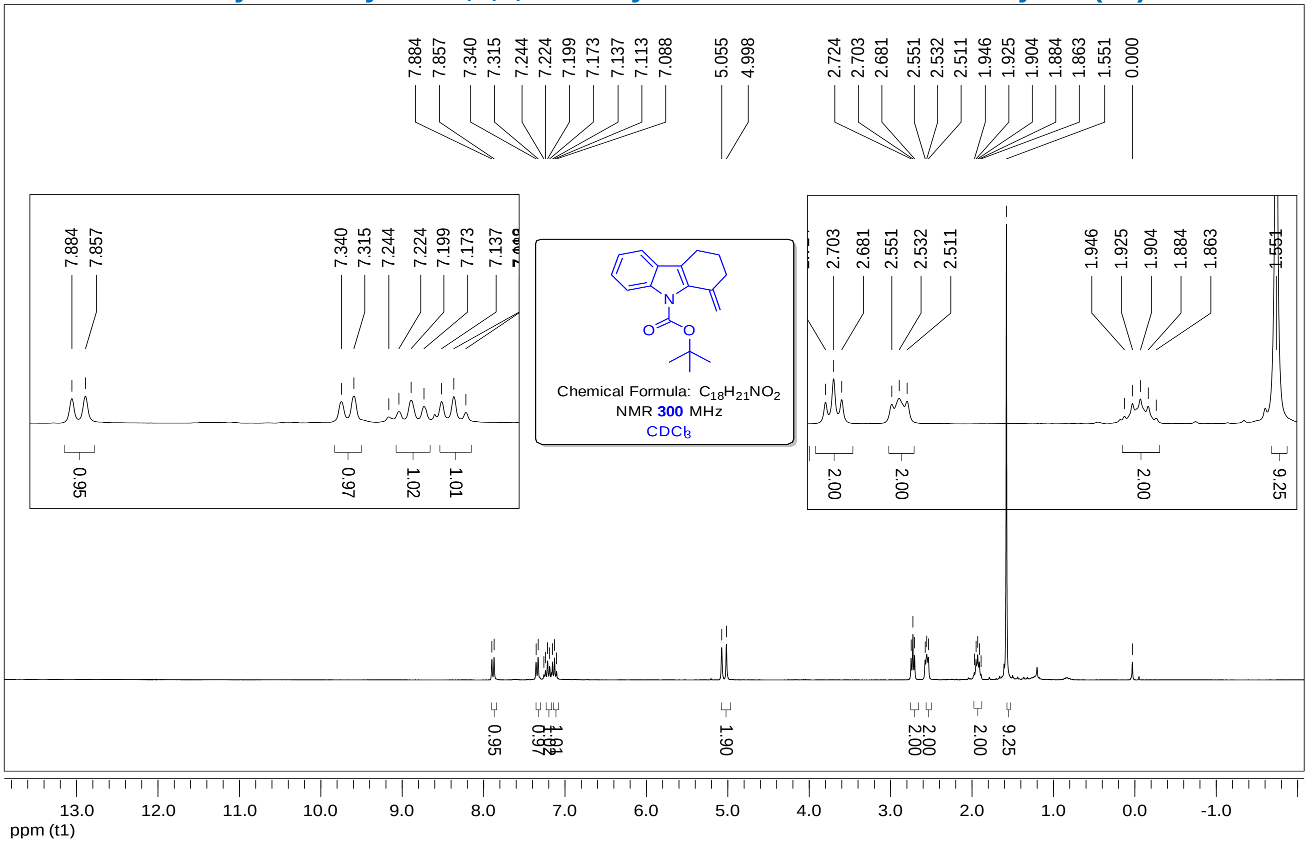

t-Butyl 1-methylene-1,2,3,4-tetrahydro-9*H*-carbazole-9-carboxylate (5h)

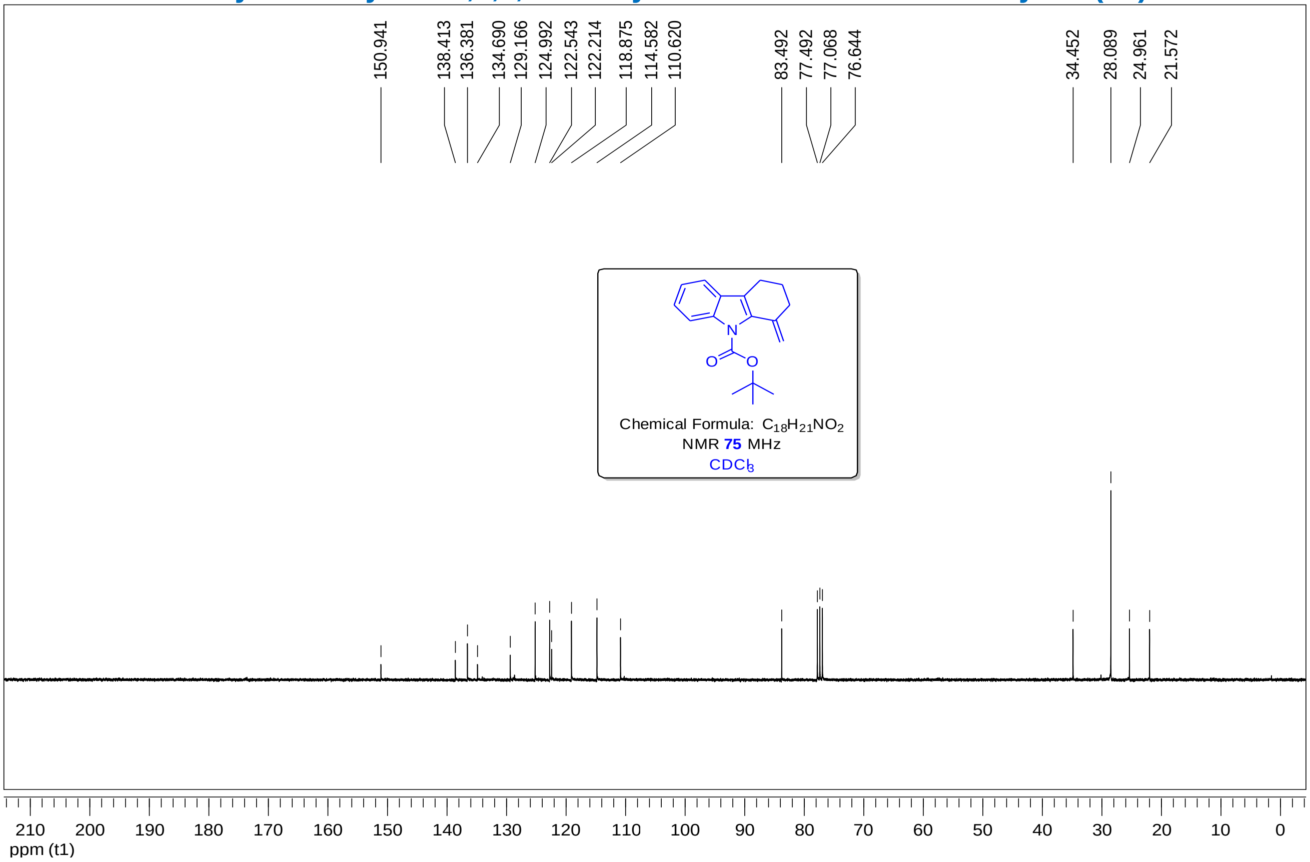

1-Methylene-2,3,4,9-tetrahydro-1*H*-carbazole (5i)

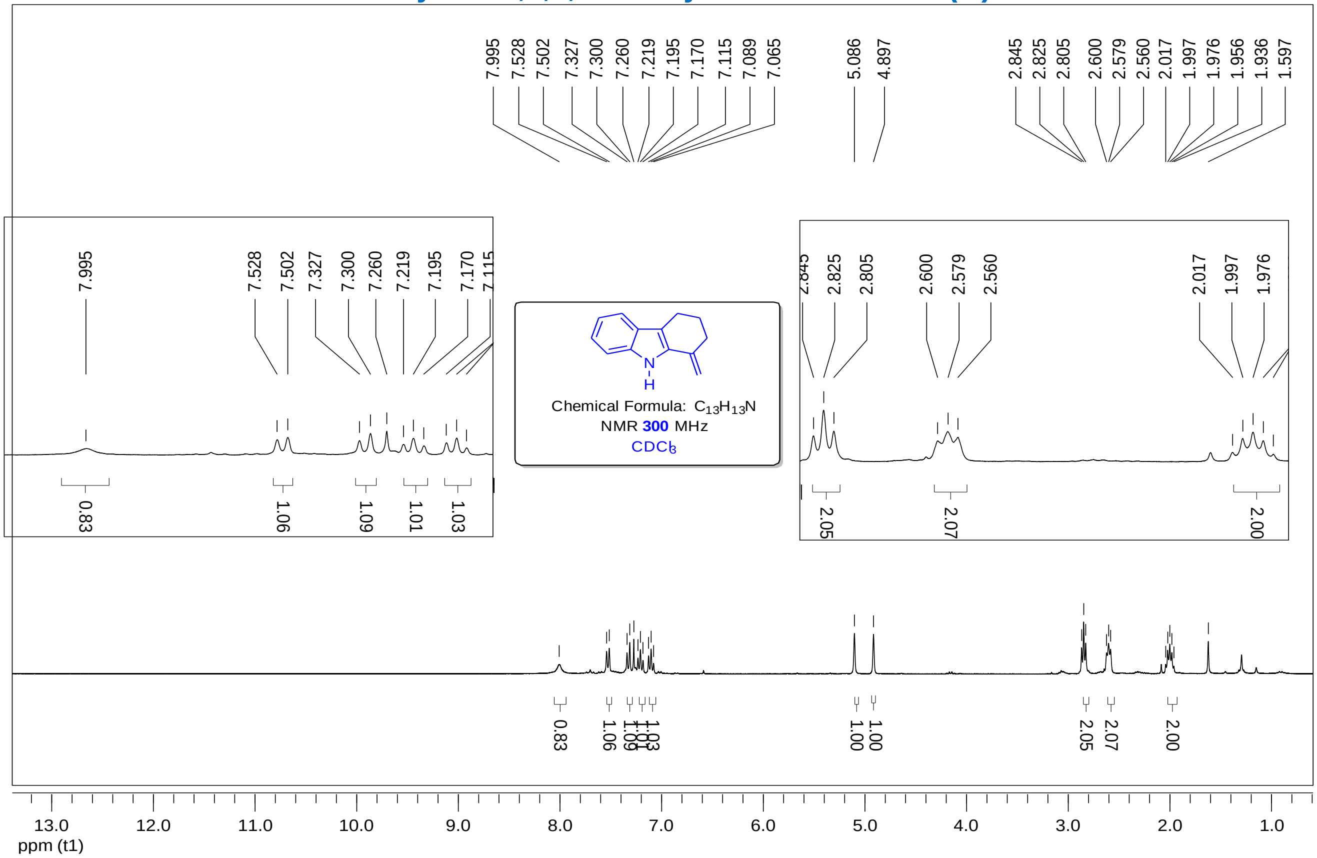

1-Methylene-2,3,4,9-tetrahydro-1*H*-carbazole (5i)

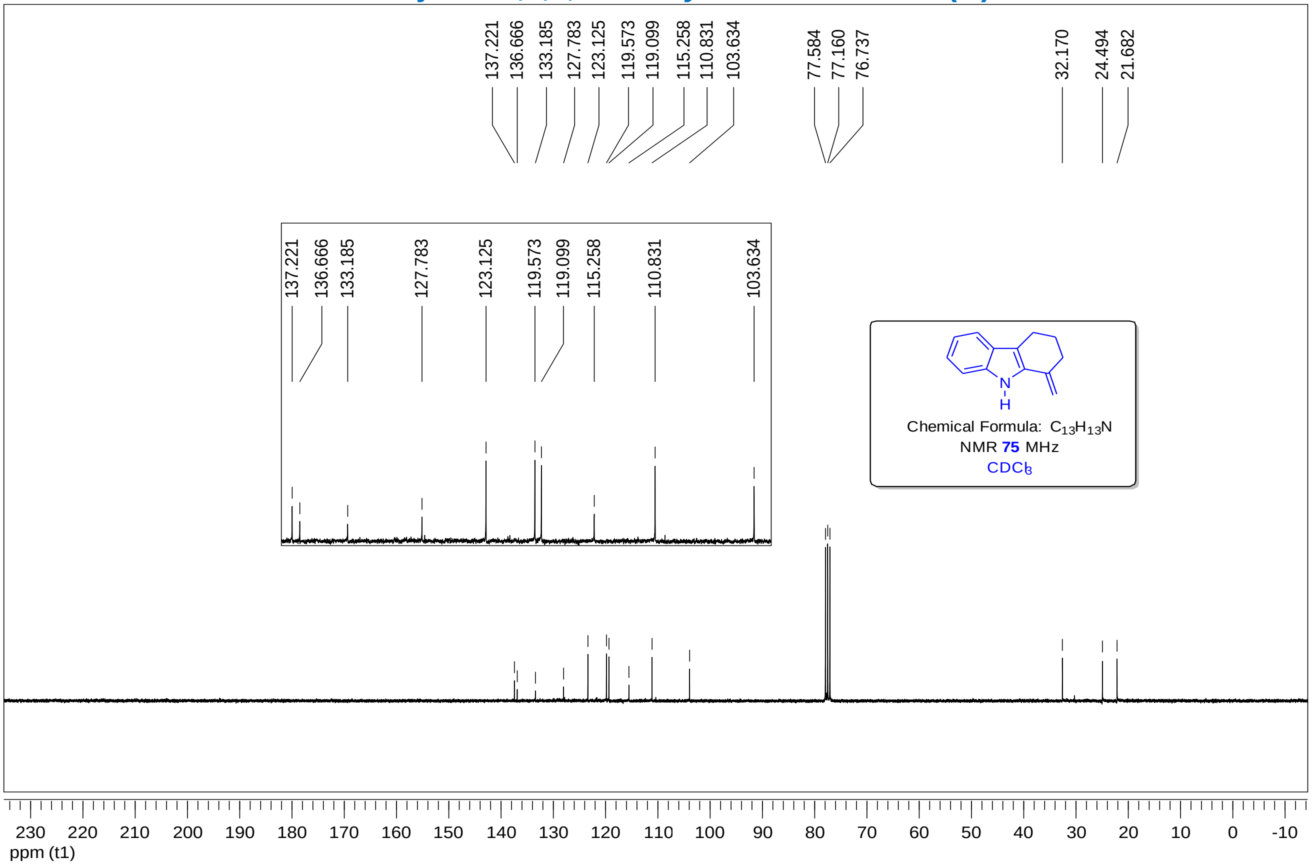

5-Methylene-5,6,7,8-tetrahydroisoquinoline (5j)

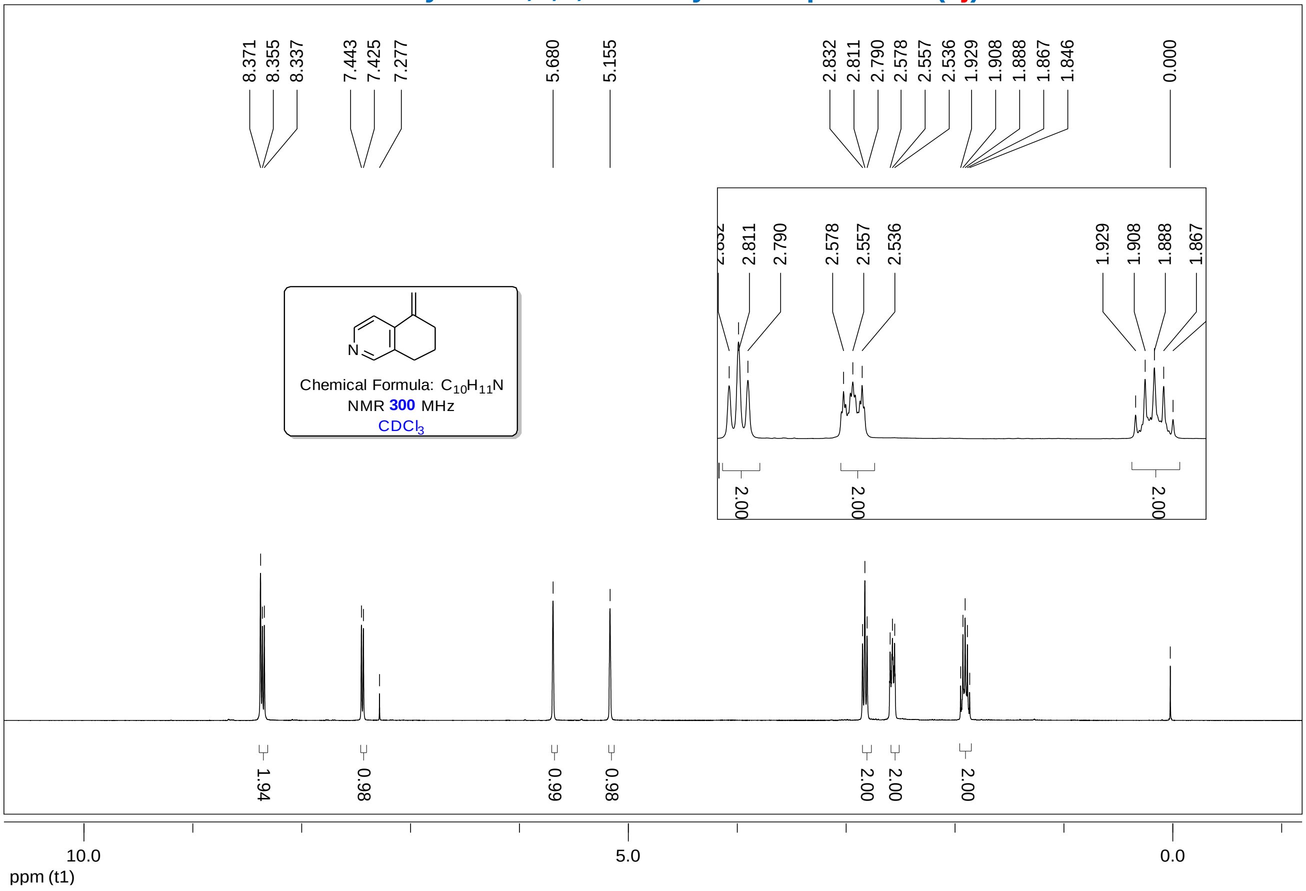

5-Methylene-5,6,7,8-tetrahydroisoquinoline (5j)

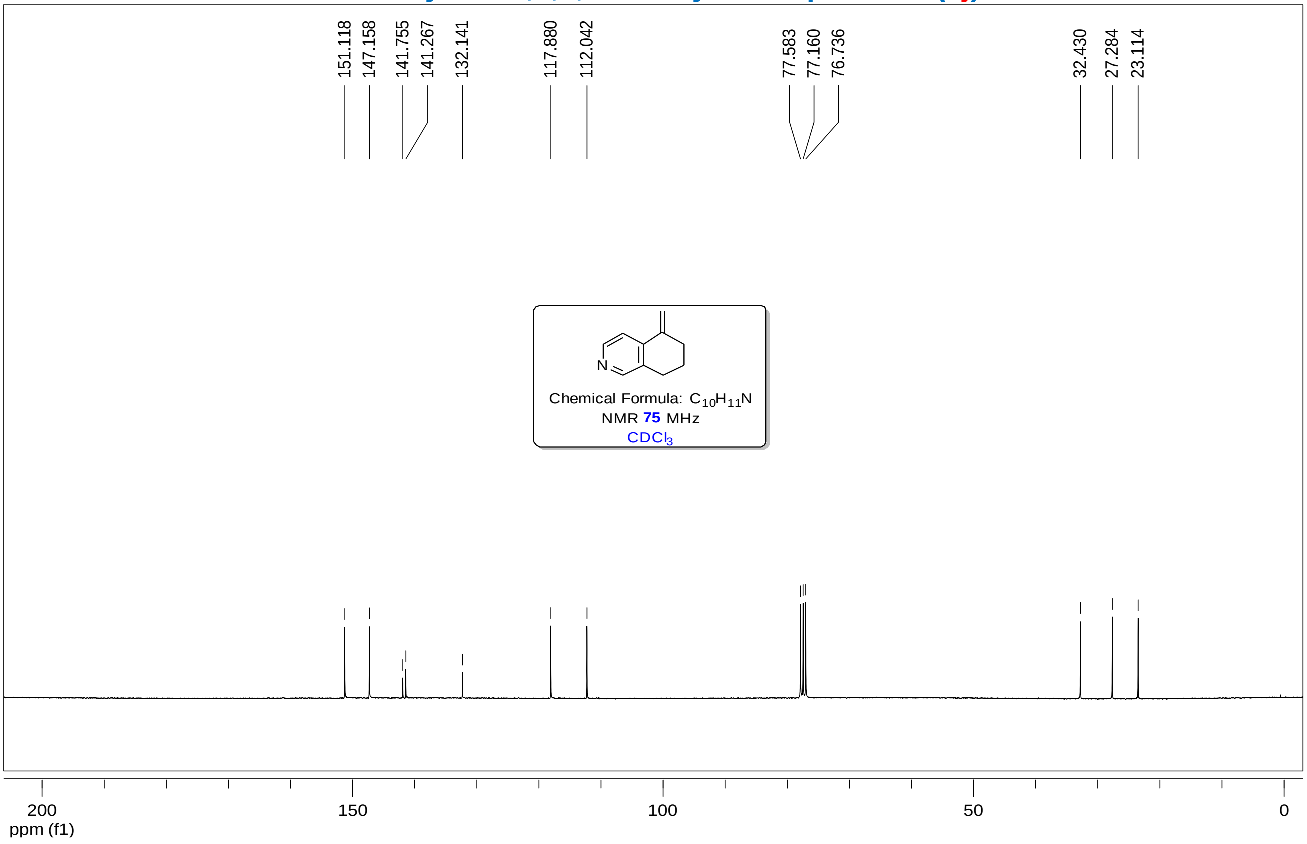

8-Methylene-5,6,7,8-tetrahydroisoquinoline (5k).

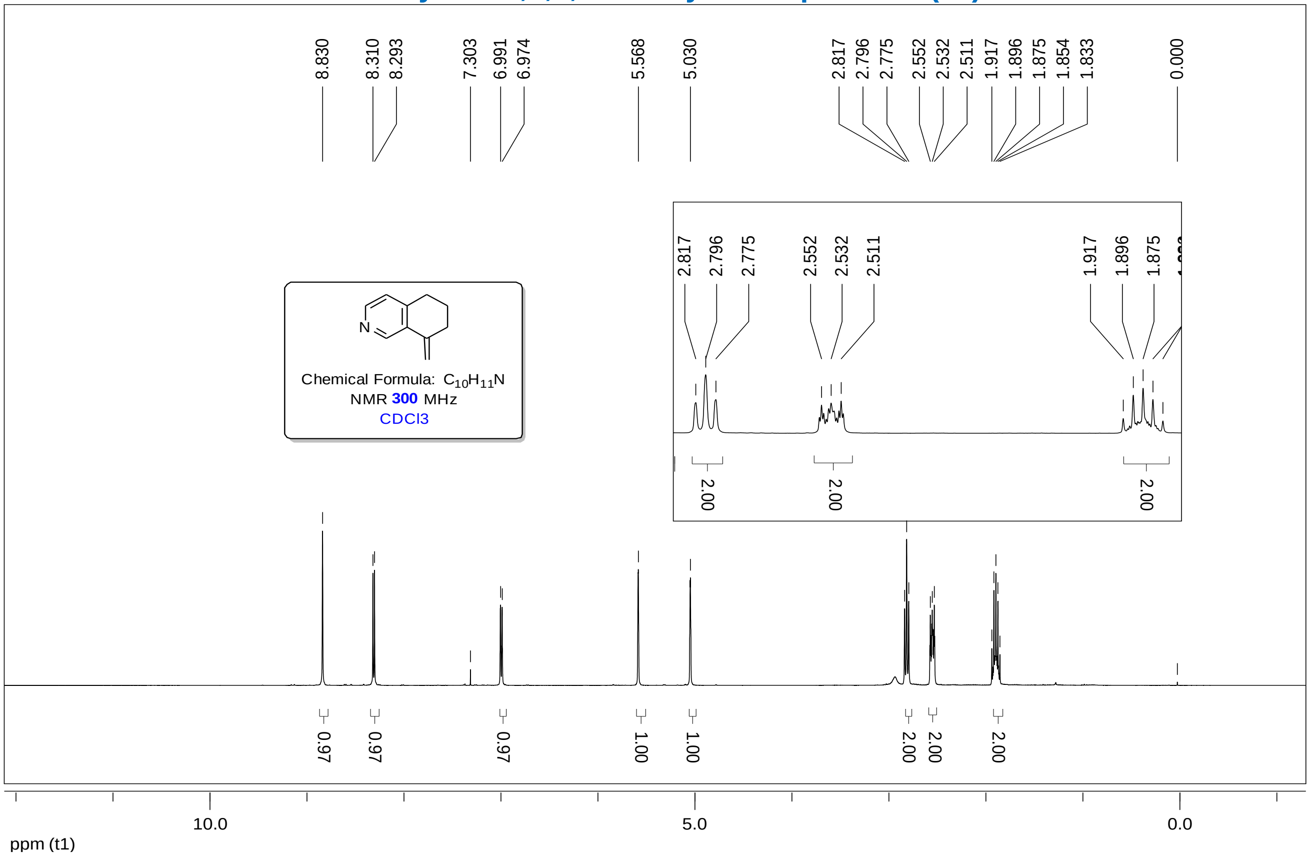

8-Methylene-5,6,7,8-tetrahydroisoquinoline (5k).

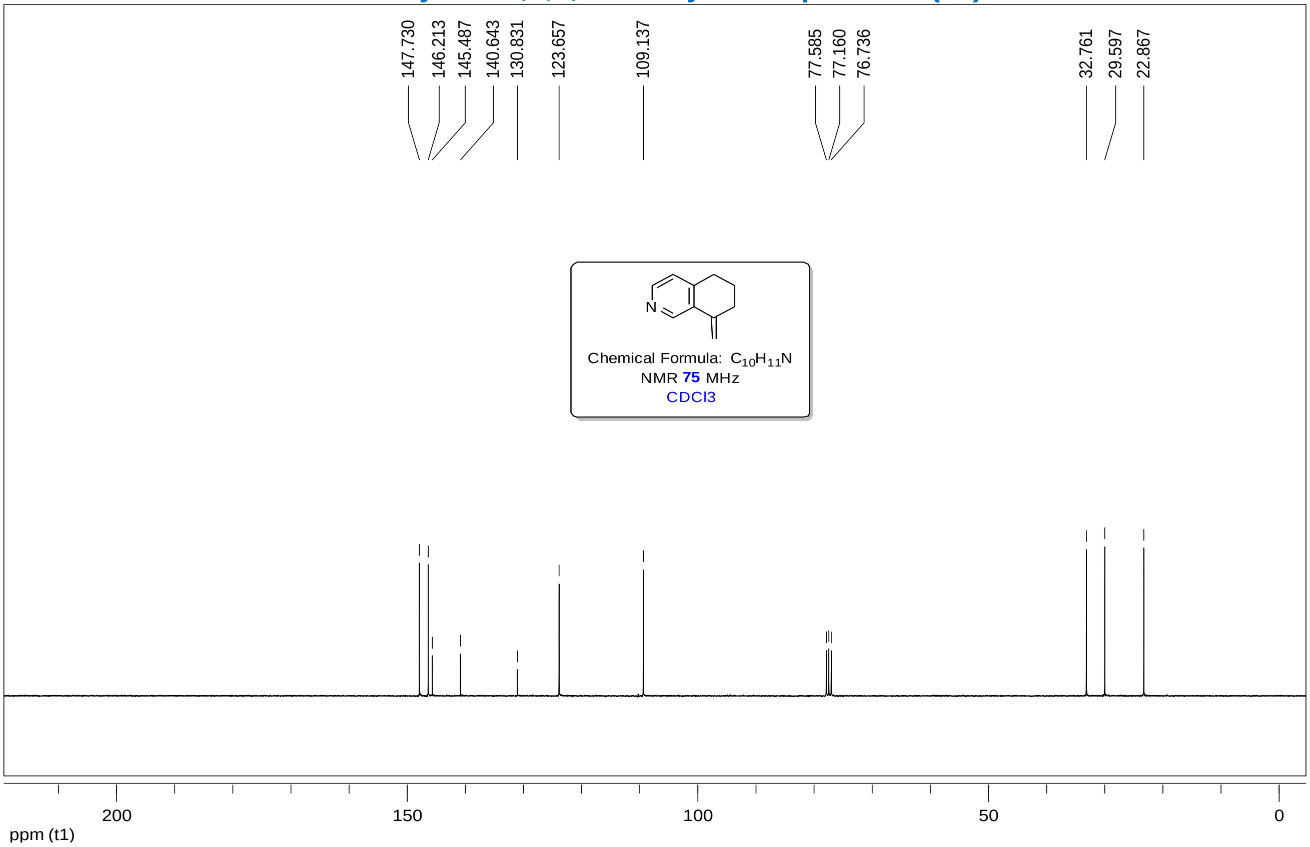

4-Methylenethiochroman (5l)

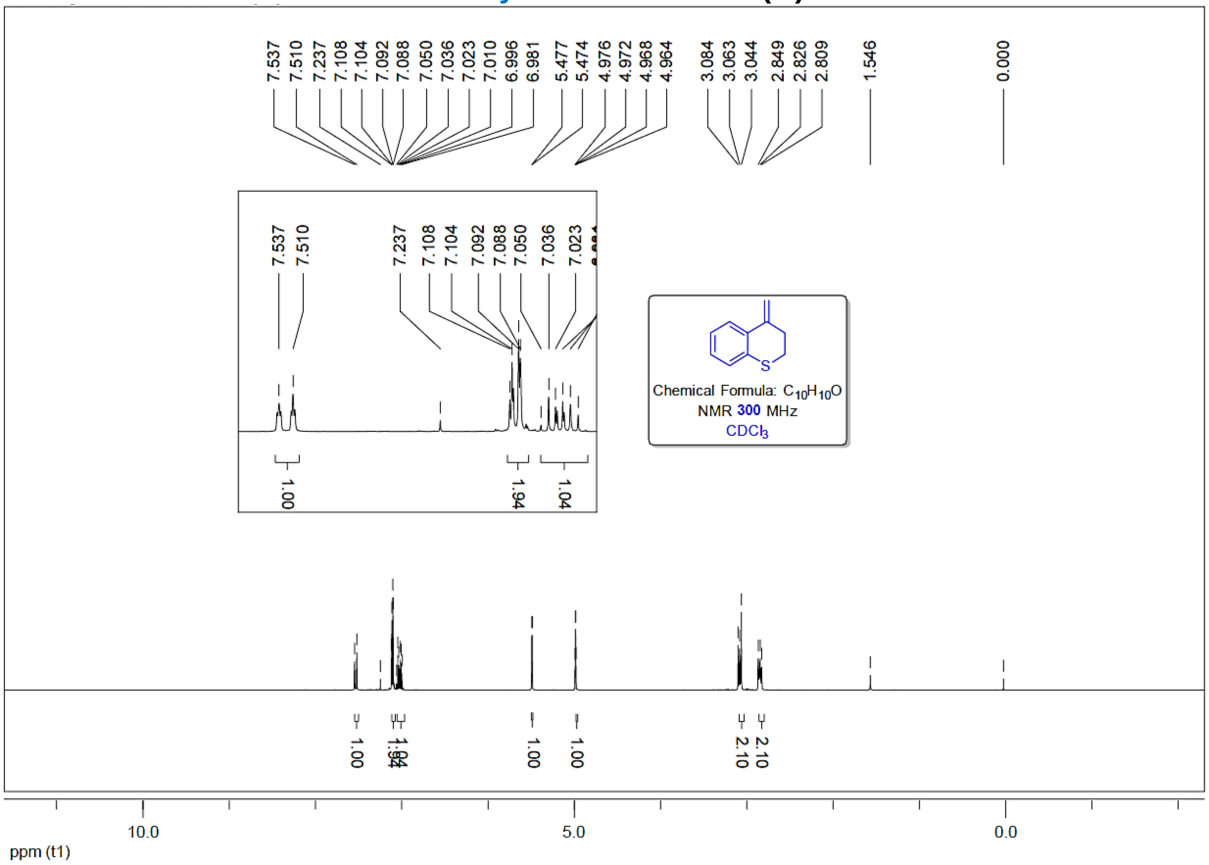

4-Methylenethiochroman (5l)

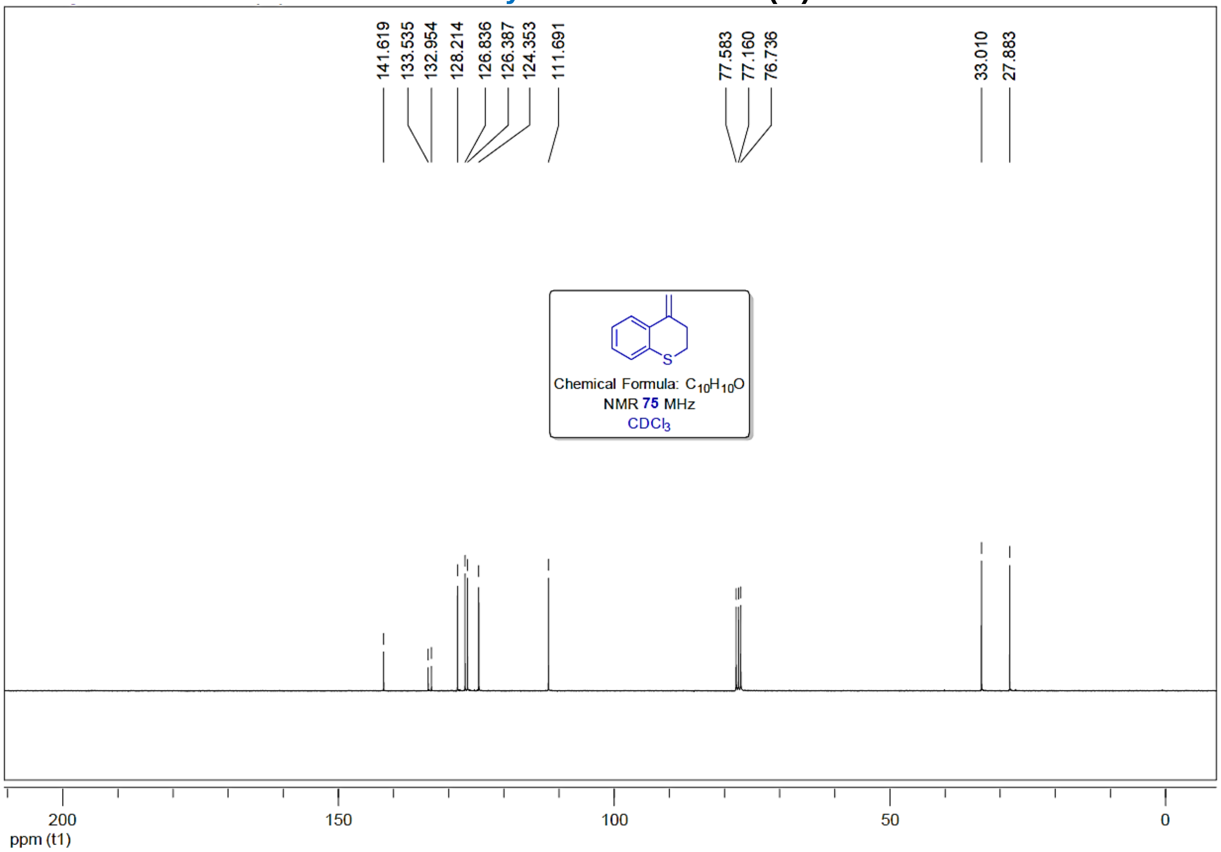

6-Fluoro-4-methylenechroman (5m).

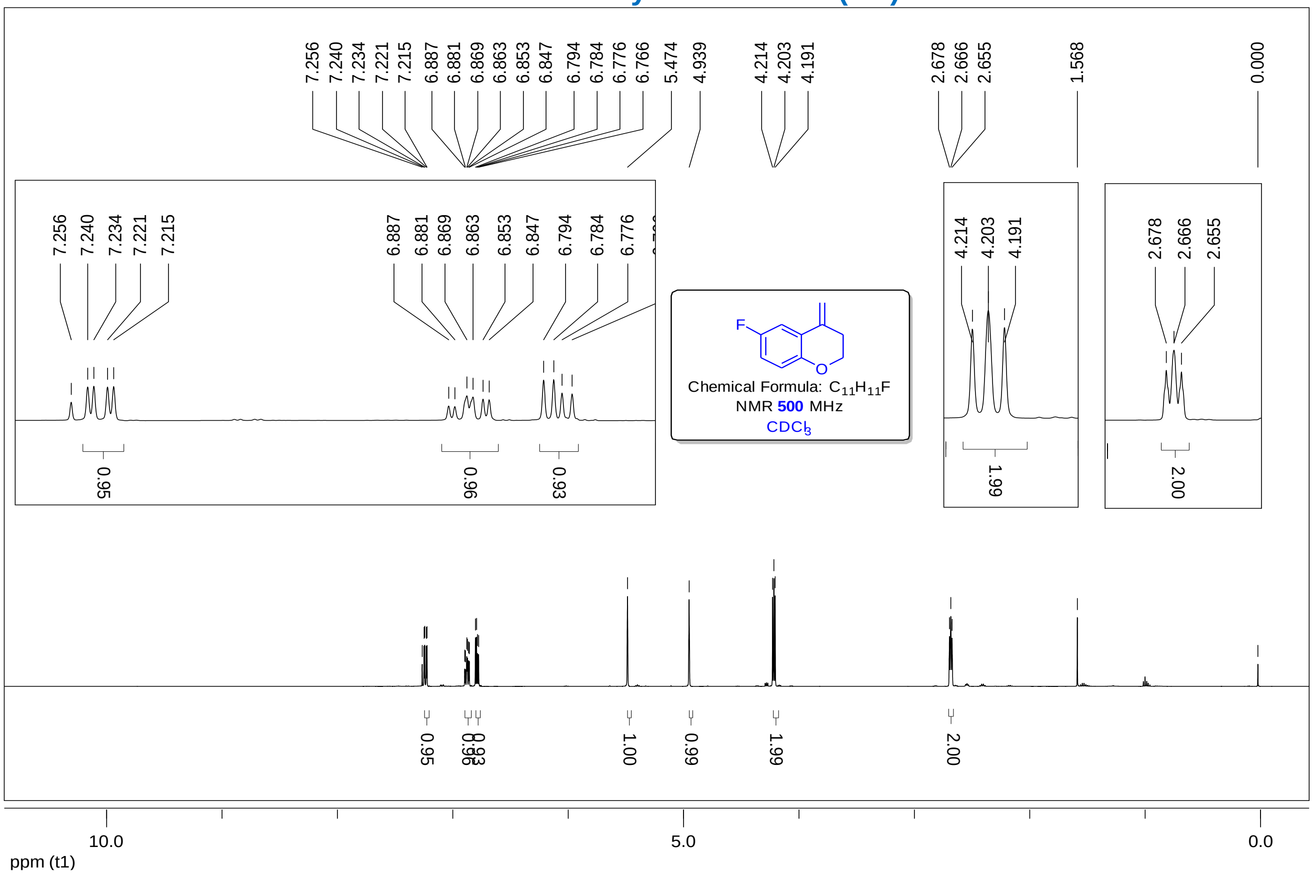

6-Fluoro-4-methylenechroman (5m).

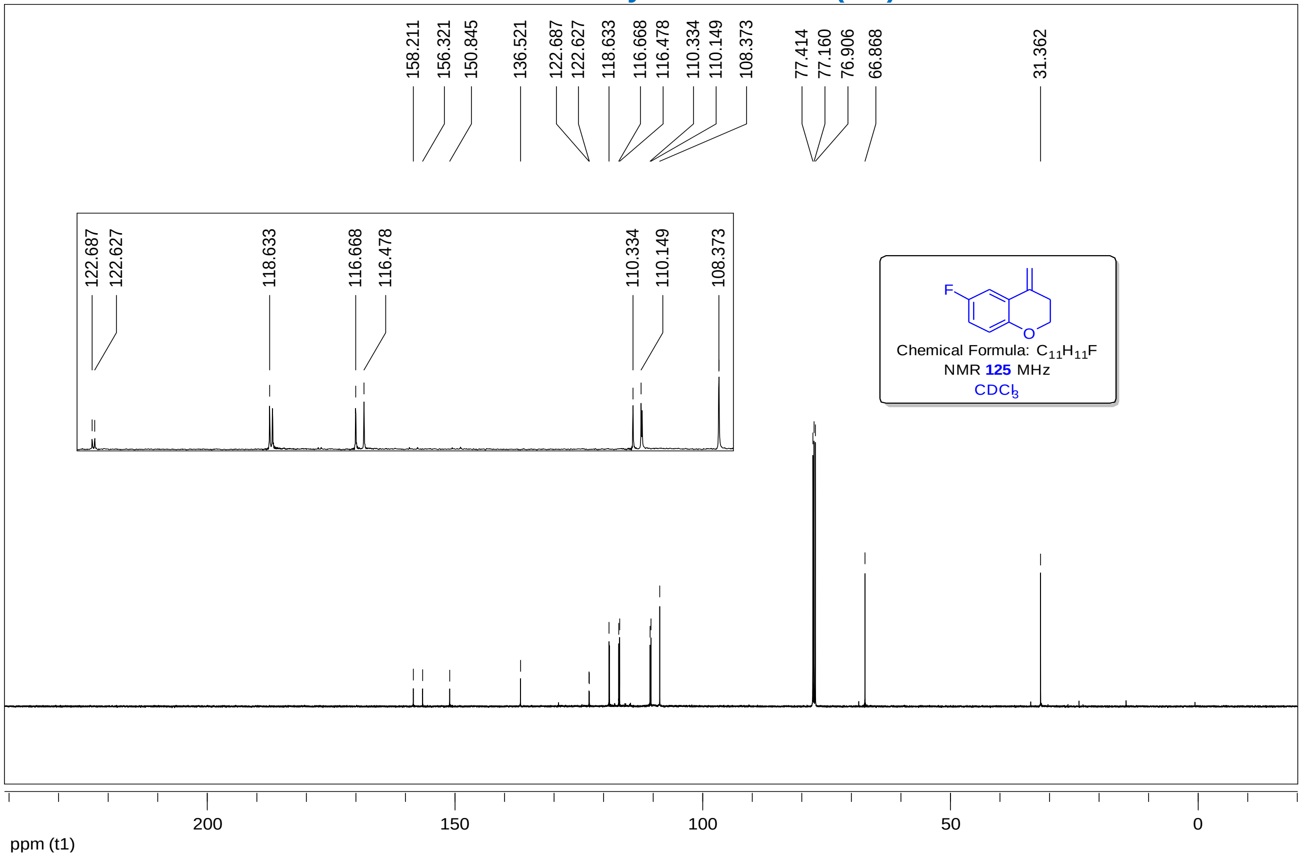

7-Methoxy-2-phenyl-2,3-dihydrobenzo[b]oxepin-4(5*H*)-one (6n)

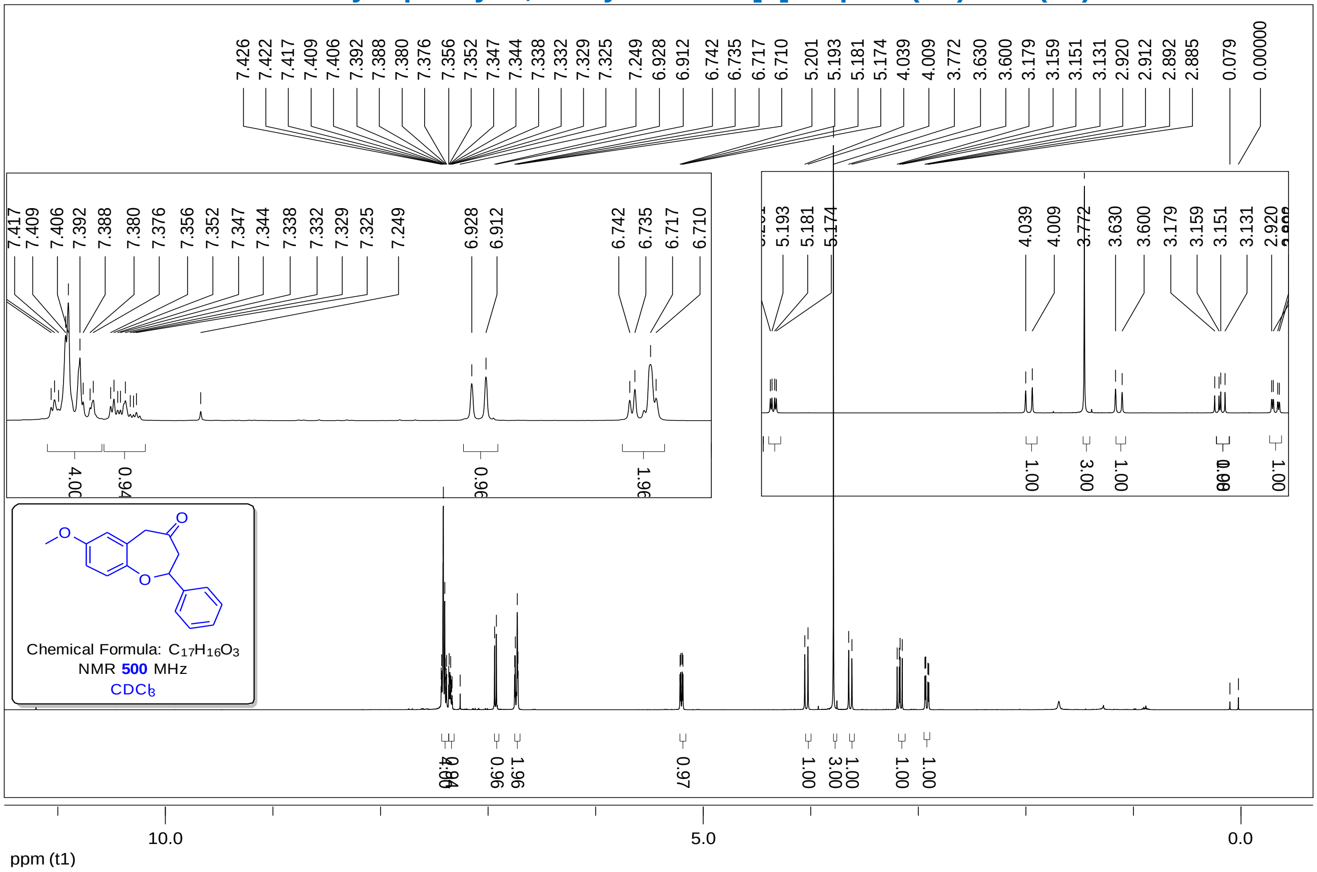

7-Methoxy-2-phenyl-2,3-dihydrobenzo[b]oxepin-4(5*H*)-one (6n)

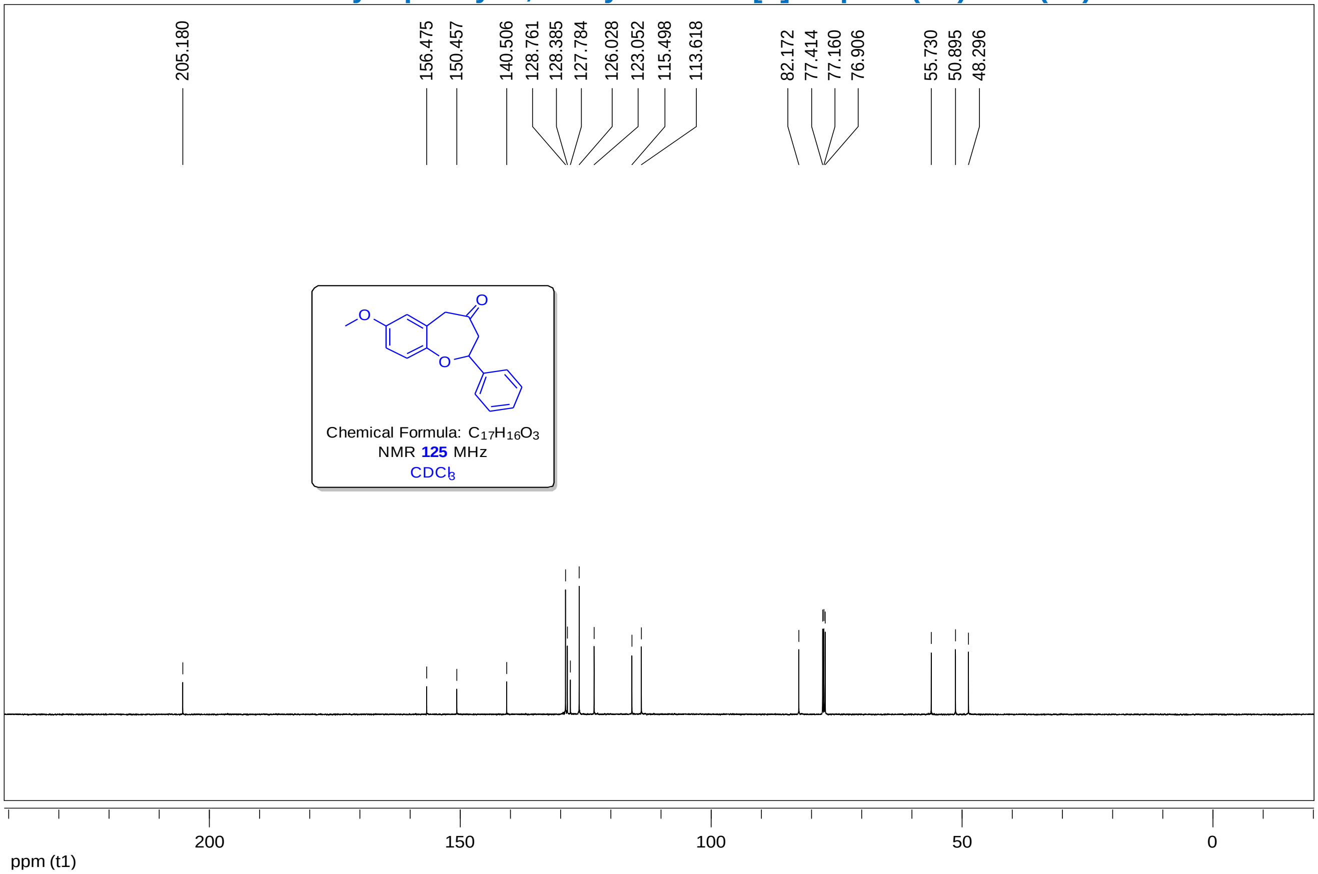

4-Methylene-1-(methylsulfonyl)-4,5,6,7-tetrahydro-1*H*-indole (5o)

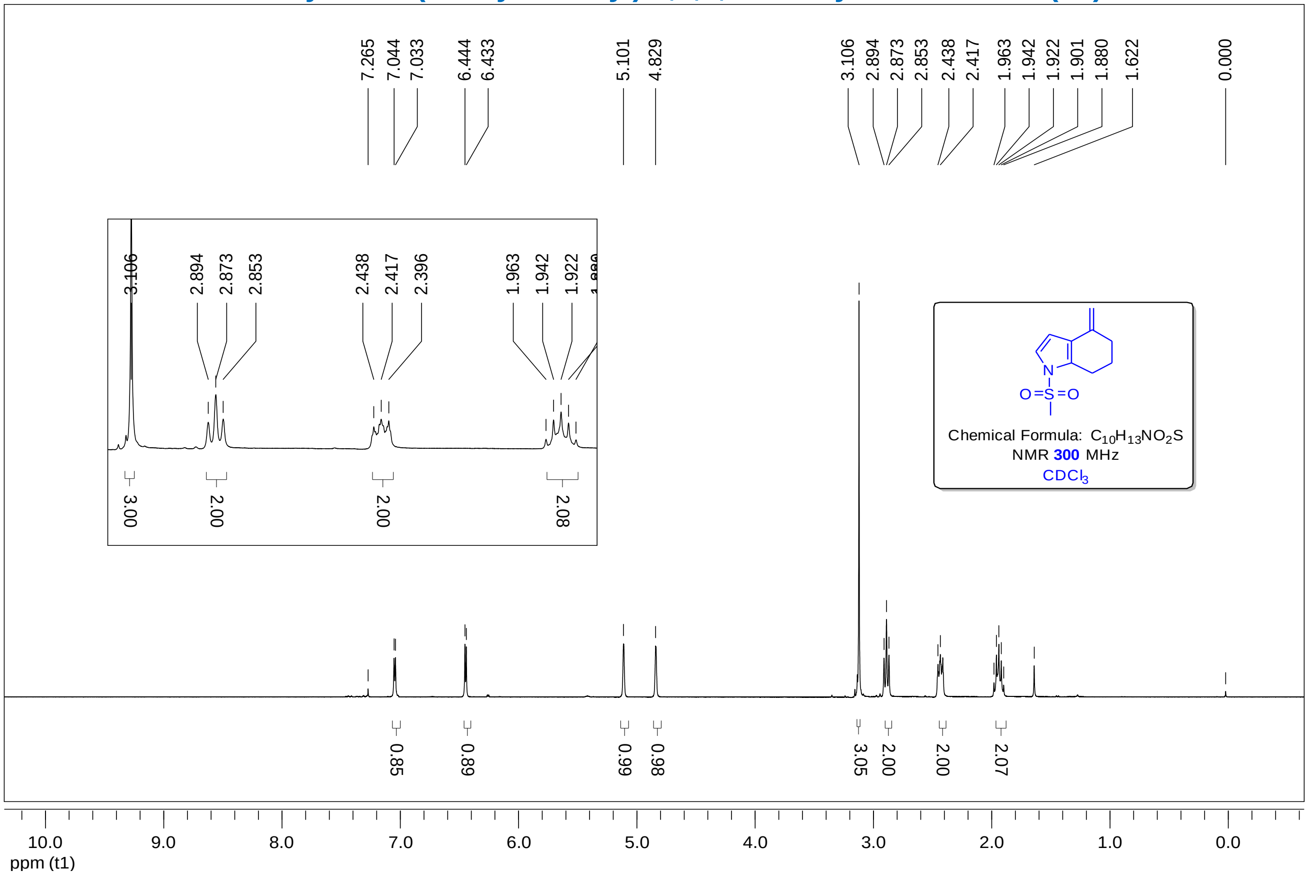

4-Methylene-1-(methylsulfonyl)-4,5,6,7-tetrahydro-1*H*-indole (5o)

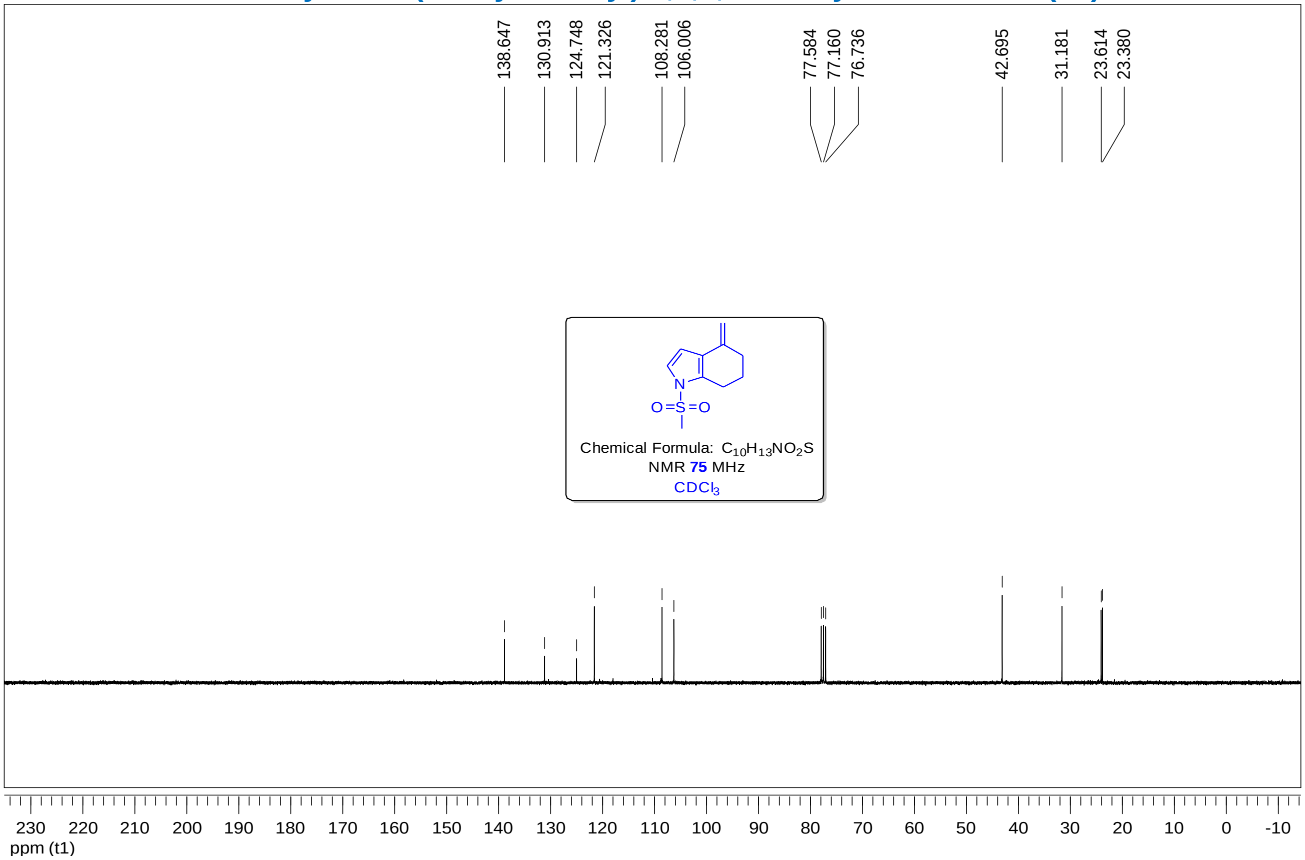

t-Butyl 4-methylene-4,5,6,7-tetrahydro-1*H*-indole-1-carboxylate (5p)

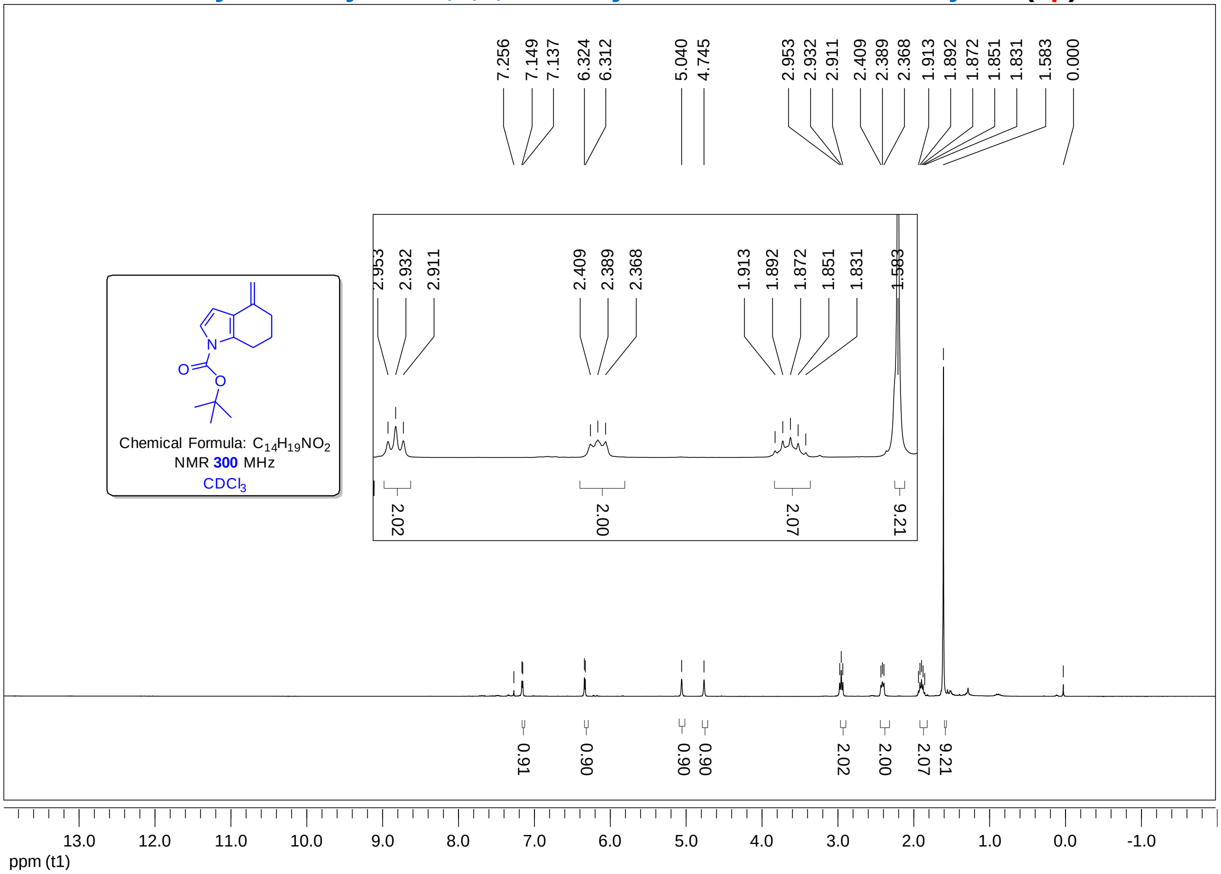

t-Butyl 4-methylene-4,5,6,7-tetrahydro-1*H*-indole-1-carboxylate (5p)

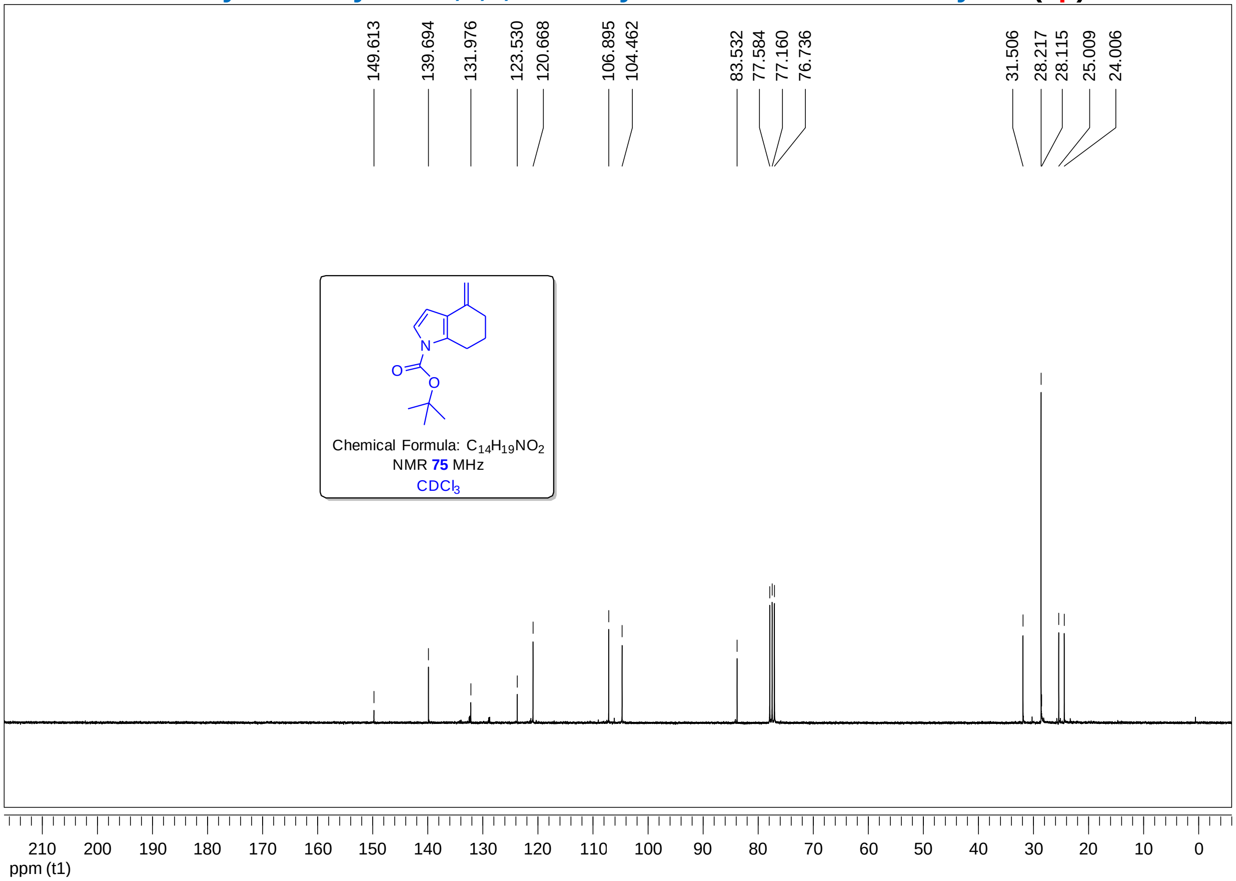

4-Methylenethiochroman (5l)

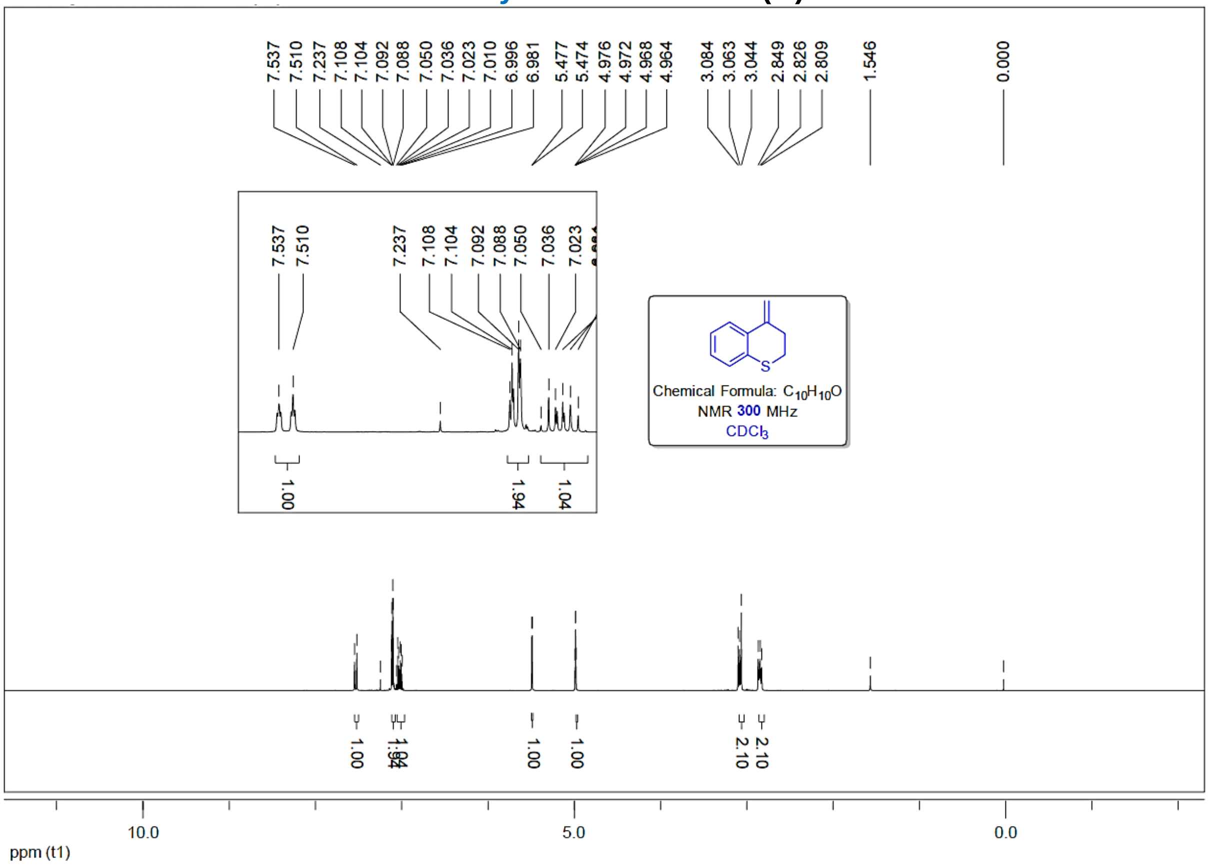

4-Methylenethiochroman (5l)

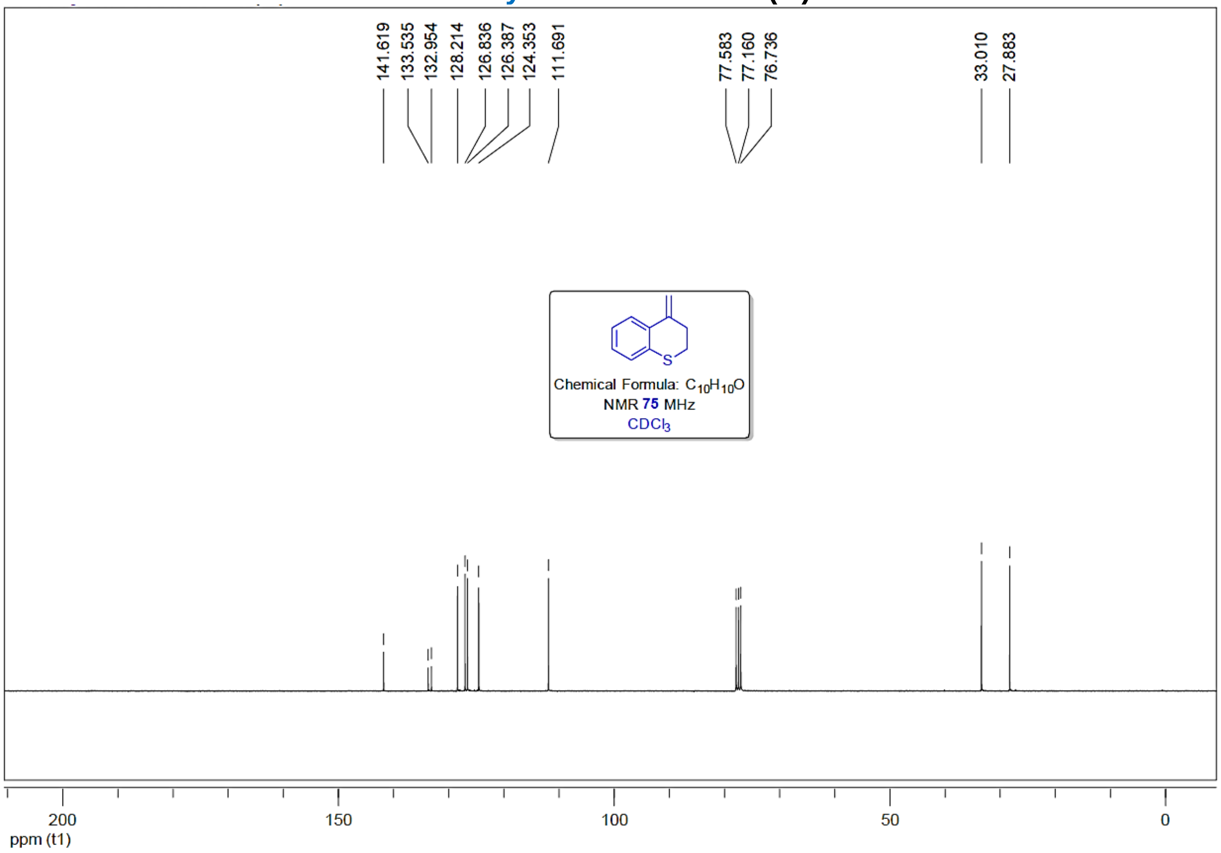

References

1. Lee, Y.R.; Morehead, A.T. A New Route for the Synthesis of Furanoflavone and Furanochalcone Natural Products. *Tetrahedron* **1995**, *51*, 4909–4922.
2. Arai, M.; Miyauchi, Y.; Miyahara, T.; Ishikawa, T.; Saito, S. Synthesis of 4-Acetoxyindoles and Related Derivatives by Means of Air Oxidation of 4-Oxo-4,5,6,7-Tetrahydroindoles Obtained from Nitroalkenes and Cyclohexane-1,3-Diones. *Synlett* **2008**, *2009*, 122–126.
3. Kadam, S.A.; Haav, K.; Toom, L.; Haljasorg, T.; Leito, I. NMR Method for Simultaneous Host-Guest Binding Constant Measurement. *J. Org. Chem.* **2014**, *79*, 2501–2513.
4. de Candia, M.; Zaetta, G.; Denora, N.; Tricarico, D.; Majellaro, M.; Cellamare, S.; Altomare, C.D. New Azepino[4,3-b]Indole Derivatives as Nanomolar Selective Inhibitors of Human Butyrylcholinesterase Showing Protective Effects against NMDA-Induced Neurotoxicity. *Eur. J. Med. Chem.* **2017**, *125*, 288–298.
5. Davies, H.M.L.; Manning, J.R. C-H Activation as a Strategic Reaction: Enantioselective Synthesis of 4-Substituted Indoles. *J. Am. Chem. Soc.* **2006**, *128*, 1060-1061.
6. Zuo, Y.; He, X.; Ning, Y.; Wu, Y.; Shang, Y. Rh(III)-Catalyzed C–H Activation/Intramolecular Cyclization: Access to N -Acyl-2,3-Dihydro-1 H -Carbazol-4(9 H)-Ones from Cyclic 2-Diazo-1,3-Diketones and N -Arylamides. *ACS Omega* **2017**, *2*, 8507–8516.
7. Cho, H., Iwama, Y., Sugimoto, K., Mori, S. and Tokuyama, H. Regioselective Synthesis of Heterocycles Containing Nitrogen Neighboring an Aromatic Ring by Reductive Ring Expansion Using Diisobutylaluminum Hydride and Studies. *J. Org. Chem* **2010**, *75*, 627–636.
8. Bardakos, V.; Sucrow, W. Enhydrazine, 21: Lactame Aus 1,2,3,9-Tetrahydro-4H-Carbazol-4-Onen. *Chem. Ber.* **1978**, *111*, 853–859.
9. Montalban, A.G.; Baum, S.M.; Cowell, J.; McKillop, A. Formation of N-Substituted 4-and 7-Oxo-4, 5, 6, 7-Tetrahydroindoles Revisited: A Mechanistic Interpretation and Conversion into 4-and 7-Oxoindoles. *Tetrahedron Lett.* **2012**, *53*, 4276–4279.
10. Laurila, M.L., Magnus, N.A. and Staszak, M.A. Green N-Methylation of Electron Deficient Pyrroles with Dimethylcarbonate. *Org. Process Res. Dev.* **2009**, *13*, 1199–1201.
11. Lee, I.-S.H. Synthesis of N-Aryl-4,5,6,7-Tetrahydroindoles. *Korean Chem. Soc.* **2012**, *33*, 341–343.
12. McEachern, E.J.; Yang, W.; Chen, G.; Skerlj, R.T.; Bridger, G.J. Convenient Synthesis of 5, 6, 7, 8-Tetrahydroquinolin-8-Ylamine and 6, 7-Dihydro-5 H-Quinolin-8-One. *Synth. Commun.* **2003**, *33*, 3497–3502.
13. Gartshore, C.J. and Lupton, D.W. Studies on the Enantioselective Synthesis of Carbazolones as Intermediates in Aspidosperma and Kopsia Alkaloid Synthesis. *Aust. J. Chem.* **2013**, *66*, 882–890.
14. Piras, L.; Ghiron, C.; Minetto, G.; Taddei, M. Microwave-Assisted Synthesis of Tetrahydroindoles. *Tetrahedron Lett.* **2008**, *49*, 459–462.
15. Khan, A.; Silva Jr, L.F.; Rabnawaz, M. Iodine (III)- Promoted Ring Expansion Reactions: A Metal- Free Approach toward Seven- Membered Heterocyclic Rings. *Asian J. Org. Chem.* **2021**, *10*, 2549–2552.
